# TCR/CD3-based synthetic antigen receptors (TCC) convey superior antigen sensitivity combined with high fidelity of activation

**DOI:** 10.1126/sciadv.adj4632

**Published:** 2024-09-04

**Authors:** Vanessa Mühlgrabner, Timo Peters, Rubí M.-H. Velasco Cárdenas, Benjamin Salzer, Janett Göhring, Angelika Plach, Maria Höhrhan, Iago Doel Perez, Vasco Dos Reis Goncalves, Jesús Siller Farfán, Manfred Lehner, Hannes Stockinger, Wolfgang W. Schamel, Kilian Schober, Dirk H. Busch, Michael Hudecek, Omer Dushek, Susana Minguet, René Platzer, Johannes B. Huppa

**Affiliations:** ^1^ Medical University of Vienna, Center for Pathophysiology, Infectiology and Immunology, Institute for Hygiene and Applied Immunology, Vienna, Austria.; ^2^ Department of Immunology, Faculty of Biology, University of Freiburg, Germany.; ^3^ Center for Biological Signaling Studies (BIOSS), University of Freiburg, Germany.; ^4^ Center for Integrative Biological Signaling Studies (CIBSS), University of Freiburg, Germany.; ^5^ St. Anna Children’s Cancer Research Institute (CCRI), 1090, Vienna, Austria.; ^6^ Christian Doppler Laboratory for Next Generation CAR T Cells, 1090, Vienna, Austria.; ^7^ Medizinische Klinik und Poliklinik II, Universitätsklinikum Würzburg, Würzburg, Germany.; ^8^ Sir William Dunn School of Pathology, University of Oxford, South Parks Road, Oxford, UK.; ^9^ Mikrobiologisches Institut–Klinische Mikrobiologie, Immunologie und Hygiene, Universitätsklinikum Erlangen, Friedrich-Alexander-Universität Erlangen-Nürnberg, Erlangen, Germany.; ^10^ Institute for Medical Microbiology, Immunology and Hygiene, Technical University of Munich, Munich, Germany.

## Abstract

Low antigen sensitivity and a gradual loss of effector functions limit the clinical applicability of chimeric antigen receptor (CAR)–modified T cells and call for alternative antigen receptor designs for effective T cell–based cancer immunotherapy. Here, we applied advanced microscopy to demonstrate that TCR/CD3-based synthetic constructs (TCC) outperform second-generation CAR formats with regard to conveyed antigen sensitivities by up to a thousandfold. TCC-based antigen recognition occurred without adverse nonspecific signaling, which is typically observed in CAR–T cells, and did not depend—unlike sensitized peptide/MHC detection by conventional T cells—on CD4 or CD8 coreceptor engagement. TCC-endowed signaling properties may prove critical when targeting antigens in low abundance and aiming for a durable anticancer response.

## Teaser

TCC conveyed enhanced antigen sensitivity and fidelity of activation when compared to chimeric antigen receptors.

## INTRODUCTION

Redirecting T cell specificity toward tumor-associated antigens (TAAs) via chimeric antigen receptors (CARs) has proven an effective treatment strategy for patients with relapsed and refractory acute lymphoblastic leukemia, non-Hodgkin lymphoma, or multiple myeloma (*1*–*5*). While long-term progression-free survival does frequently occur following CAR–T cell therapy (CAR T), relapsing disease is also common among treated patients, limiting overall effectiveness (*4*, *6*, *7*). The main two mechanisms underlying cancer reemergence after CAR T concern tumor-intrinsic antigen escape (*2*, *6*, *8*–*10*) as caused by down-regulation or by complete loss of TAA expression, as well as long-term CAR T cell exhaustion (*11*, *12*) resulting from aggravated tonic CAR-mediated signaling.

Being equipped with a synthetic antigen receptor, CAR T cells require 1000 or more TAAs for activation (*13*). This contrasts the remarkable capacity of conventional T cells to detect via their naturally evolved T cell antigen receptors (TCRs) the presence of even a single antigenic peptide-loaded major histocompatibility complex (pMHC), which are vastly outnumbered by structurally similar yet nonstimulatory bystander pMHC (*14*–*16*).

The biophysical and cell biological mechanisms underlying sensitized T cell antigen recognition are not well understood, especially in view of the rather transient nature of stimulatory TCR-pMHC interactions. The latter typically feature lifetimes in the order of seconds and micromolar rather than antibody-like nanomolar affinities. The persistent gap in understanding is in large part caused by the complexities inherent to TCR triggering and proximal signaling: Unlike receptor tyrosine kinases, the TCR is devoid of intrinsic kinase activity (*17*) and lacks—unlike CARs—cytoplasmic signaling domains. It is instead noncovalently associated with the invariant chains of the CD3 complex, namely, a CD3γε and a CD3δε heterodimer as well as a disulfide-linked CD3ζ homodimer (*18*–*20*), which contain, among other intracellular cytoplasmic motifs, so-termed immunoreceptor tyrosine-based activation motifs (ITAMs). The latter become tyrosine-phosphorylated upon canonical signaling by the TCR-proximal kinase Lck, which is also associated with the cytoplasmic tails of CD4 or CD8 coreceptors (*21*). This may explain why simultaneous extracellular coreceptor engagement with a nonpolymorphic region within the same MHC is often critical for sensitized antigen detection via TCR/CD3 (*15*, *16*, *22*). In a pivotal subsequent step, zeta chain–associated protein kinase 70 (ZAP70) binds via its two SH2 domains to phosphorylated ITAMs (pITAMs). Once associated with pITAMs on CD3, ZAP70 becomes activated by Lck or other already CD3 recruited and activated ZAP70 copies (*23*). Downstream signaling is highly amplified through ZAP70-mediated tyrosine phosphorylation of adapter proteins and signaling effectors as well as costimulatory signaling cascades involving Tec family kinases (*24*).

CAR signaling resembles TCR signaling in many but not all aspects, which likely explains reduced sensitivity and long-term CAR T cell fitness. Membrane-proximal costimulatory signaling modules adopted from CD28, 4-1BB, or OX40, which are entirely missing from the TCR/CD3 complex, keep the ITAM-containing CD3ζ-derived signaling module at a greater distance from the plasma membrane with conceivable consequences for Lck accessibility. Furthermore, most of the CARs’ matching TAAs do not support coreceptor-mediated sensitization for lack of a coreceptor-binding site. In addition, the total number of ITAMs available for signaling amounts in monomeric CARs to only 30% and in dimeric CARs to 60% of that of a fully assembled plasma membrane–resident TCR/CD3 complex. Moreover, spatial ITAM arrangement differs, ITAM diversity is reduced, and other CD3 signaling motifs are entirely absent, which may affect not only efficient ZAP70 activation but also the degree of tonic signaling and hyperactivity in the absence of antigens. The rapid recruitment of the E3 ubiquitin ligase c-Cbl to CD3ε’s intracellular proline-rich domain keeps nonspecific TCR-proximal signaling in the absence of antigen at nondetectable levels (*25*). On the other hand, transient TCR-pMHC binding not only triggers TCR/CD3 effectively but also allows for serial triggering of many TCRs by a single antigenic ligand. In contrast, CAR signaling requires stable TAA binding, which limits in turn the overall number of triggered CARs to that of available TAAs.

Sidestepping such principal CAR-associated shortcomings may hence require a radical overhaul of conventional CAR architectures. A rational approach would capitalize on the TCR/CD3 framework as a means to exploit TCR-associated and evolutionary preserved features of sensitized antigen detection and low tonic signaling for improved clinical efficacy. Following this rationale, several groups have fused TAA-targeting single-chain variable fragments (scF_V_s) by various means to the native TCR/CD3 complex. TCR/CD3-based synthetic constructs (TCC) including “T cell antigen coupler,” “antibody T cell receptor,” “T cell receptor fusion constructs” (TRuC), and “synthetic T cell receptor and antigen receptor” (STAR) were all reported to enhance the antitumor response (*26*–*29*). Consistent with these observations, soluble bispecific T cell engagers, which directly link the TCR/CD3 complex to TAAs on target cells, have shown high clinical efficacy against B cell malignancies and uveal melanoma. Now, experimental therapies target colon, gastric, prostate, ovarian, lung, and pancreatic cancers (*30*).

Here, we have applied advanced live-cell imaging to gauge the signaling performance of TCCs in much of its unfolding complexity at the single-cell level and with single-molecule resolution. For quantitative readouts, we confronted T cells with planar glass supported lipid bilayers (SLB), which had been functionalized in experimentally defined densities with the antigen itself as well as costimulatory and adhesion molecules to serve as defined surrogate target cell for antigen recognition. We found antigen detection mediated by TCCs up to 1000 times elevated compared to that conveyed by second-generation CARs. The augmented antigen sensitivity of TCC–T cells matched those of conventional T cells, even in the absence of CD8 coreceptor engagement, and was maintained even toward lower-affinity antigens. Unlike second-generation CARs, TCC designs did not leak antigen-independent downstream signaling, a prerequisite for durable T cell functionality. However, given their superior signaling capacity, TCCs were also found to be more prone to activate T cells in response to residual off-target affinity, which must be tightly controlled for by rational protein engineering before clinical use. Together, our findings highlight fundamental advantages that are associated with the use of TCCs especially when aiming for curative T cell–based immunotherapies targeting tumor entities with low or highly heterogeneous TAA expression.

## RESULTS

### Anti-CD19 second-generation CAR-modified T cells (Tisagenlecleucel) fail to sense antigens in low abundance

**Fig. 1.**
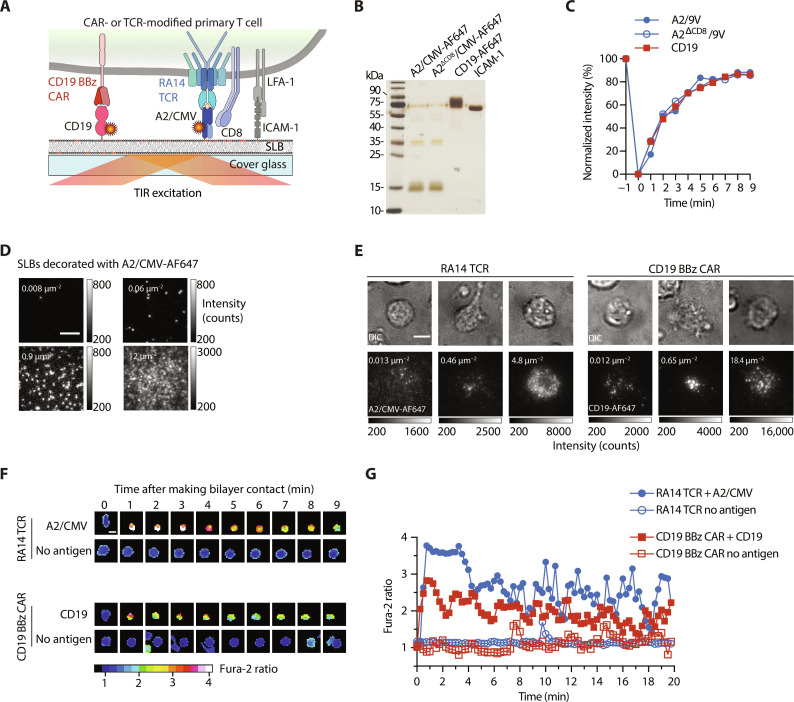
Protein-functionalized SLB-based platform for high-throughput analysis of TCR and synthetic antigen receptor–mediated activation and downstream signaling events. (**A**) Schematic representation of an SLB equipped with fluorescently labeled antigens A2/CMV or CD19, ICAM-1 adhesion molecules for recognition by RA14 TCR T cells or CD19 BBz CAR T cells. Extracellular portions of proteins are extended with a polyhistidine tag (12× His) to interact with 18:1 DGS-Ni-NTA (schematically depicted as red circles) present in the SLB. (**B**) A total of 12.5% reducing SDS–polyacrylamide gel electrophoresis followed by silver staining of the recombinant proteins used for SLB functionalization. (**C**) FRAP was used to determine the mobile fraction of SLB-anchored A2/CMV and CD19. Fluorescence intensities were normalized to the initial intensity values and plotted over time. Data shown are representative of *n* = 3 biological replicates. (**D**) Micrographs of A2/CMV-AF647–decorated SLBs with densities indicated (white). Scale bar, 5 μm. (**E**) Micrographs showing recruitment of A2/CMV-AF647 (by RA14 TCR T cells) and CD19-AF647 (by CD19 BBz CAR T cells) on SLBs featuring ICAM-1 in addition to the respective antigen. Antigen densities indicated (white). Scale bar, 5 μm. (**F**) Antigen-dependent changes in intracellular calcium concentrations as monitored by Fura-2 time-lapse microscopy in a single CD19 BBz CAR T and a single RA14 TCR T cell confronted with SLBs functionalized with ICAM-1 as well as CD19 or A2/CMV in indicated densities. Scale bar, 10 μm. (**G**) Normalized Fura-2 ratio values of T cells shown on the left as a function of time. DIC, differential interference contrast.

To assess T cell antigen sensitivities in a quantitative fashion, we confronted conventional cytomegalovirus (CMV)–specific or CD19-reactive CAR T cells with planar glass SLBs functionalized in defined numbers with antigen and accessory molecules to serve as a surrogate target cell (Fig. 1A) (*31*). SLBs featured the unsaturated lipids 1-palmitoyl-2-oleoyl-*sn*-glycero-3-phosphocholine (POPC; 98%) and also 1,2-dioleoyl-*sn*-glycero-3-[(*N*-(5-amino-1-carboxypentyl)iminodiacetic acid)succinyl] (nickel) (DGS–Ni-NTA; 2%). The latter provided a direct anchor to polyhistidine-tagged recombinant extracellular portions of the (i) antigen, e.g., HLA.A*0201 (A2) loaded with antigenic CMV-derived peptide pp65 (A2/CMV) for recognition by cognate RA14 TCR T cells or CD19 for recognition by CD19-specific CAR T cells, and (ii) the adhesion molecule intercellular adhesion molecule–1 (ICAM-1; Fig. 1, A and B). Fluorescence recovery after photobleaching (FRAP)–based experiments testified to the lateral mobility of SLB-embedded proteins (85% and higher; Fig. 1C). The use of SLBs allowed also for total internal reflection fluorescence (TIRF) microscopy, which involves directing the fluorophore-exciting laser beam at the glass-SLB interface in a critical angle to give rise to total reflection. The resulting evanescent field penetrates into the sample no more than 200 nm keeping fluorescence backgrounds sufficiently low to allow for single fluorophore detection. As shown in Fig. 1 (D and E), the use of SLBs enabled us to precisely fine-tune and quantitate the number of antigens and accessory proteins presented to T cells.

For use in our experiments, we isolated CD8^+^ T cells from peripheral blood mononuclear cells (PBMCs) of healthy donors. To confer specificity toward A2/CMV, we edited the *TRAC* locus via CRISPR-Cas9 to replace the endogenous TCR with the RA14 TCR [for a detailed description, please refer to (*32*) and Materials and Methods]. For initial functional comparison, we lentivirally transduced T cells with the US Food and Drug Administration–approved CD19 BBz CAR (Kymriah). This CAR featured the CD19-reactive FMC63 scF_V_, a CD8α-derived hinge and transmembrane domain, which was followed by an intracellular portion containing a 4-1BB costimulatory as well as the CD3ζ-derived activation domain (figs. S1 and S2, A to D). As shown in Fig. 1E, both TCR and CAR T cells formed stable immunological synapses with protein-functionalized SLBs and engaged their respective antigen as became visible by antigen accumulation.

To further confirm the SLBs’ functional integrity, we monitored the intracellular second messenger calcium in SLB-contacting T cells via ratiometric live-cell imaging with the use of the calcium-sensitive dye Fura-2 AM. The rise in intracellular calcium is essential and precedes all T cell effector functions following antigenic stimulation. As shown in Fig. 1 (F and G), both RA14 TCR T cells and CD19 CAR T cells exhibited a rapid antigen-dependent increase in intracellular calcium levels shortly after contacting SLB functionalized with ICAM-1 and optionally the corresponding antigen (A2/CMV or CD19). After reaching a peak within the first 2 min of SLB contact, the calcium signal plateaued above the baseline levels which had been set in antigen-free control experiments.

**Fig. 2.**
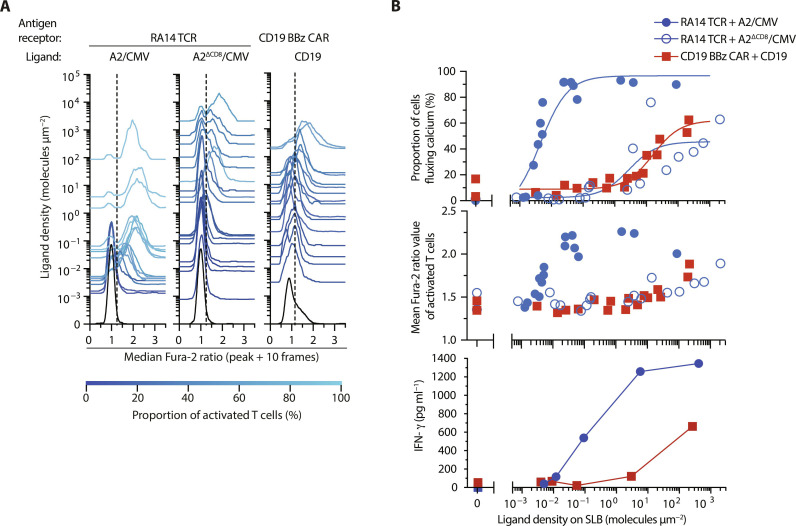
Calcium and downstream signaling response of T cells equipped with CMV-specific RA14 TCR and anti-CD19 second-generation CAR T cells. (**A**) Histograms of calcium response of RA14 TCR T cells facing A2/CMV (left), RA14 TCR T cells confronted with A2^ΔCD8^/CMV (middle), and CD19 BBz CAR T cells exposed to CD19 (right) at indicated densities. Dashed lines indicate Fura-2 ratio thresholds above which T cells were considered activated. (**B**) Top: Calcium response of RA14 TCR T cells and CD19 CAR T cells shown in (A) plotted as dose-response curves. Data were fitted to Eq. 1 as summarized in Table 1. Middle: Mean Fura-2 ratio values [taken from (A)] measured for the activated fraction of CD19 BBz CAR- and RA14 T cells confronted with SLBs featuring CD19, A2/CMV, or A2^ΔCD8^/CMV at indicated densities. Data were pooled from *n* = 2 biological replicates with *n* = 2 donors. Bottom: Quantification of IFN-γ secreted into the media after 72-hour stimulation of RA14 TCR and CD19 BBz CAR T cells with SLB-resident A2/CMV and CD19, respectively. Data are representative of one donor. Statistics: Mean ± SEM of technical duplicates.

To determine the sensitivity of RA14 TCR T cells and CD19 BBz CAR T cells toward antigen, we exposed T cells to SLBs, for which we had titrated the densities of either A2/CMV or CD19, and recorded the temporal dynamics of the ensuing calcium response at the single-cell level. More specifically, we tracked T cells for each condition using a published particle tracking algorithm (*33*). T cells, which had been in contact with the SLB for at least 6.25 min (25 frames), were included in the analysis. For each track, we determined the mean Fura-2 ratio value of 10 15-s intervals (2.5 min) following the maximum Fura-2 ratio value. Cells confronted with antigen-free SLBs served as negative control. Acquired Fura-2 ratio values were normalized to the median of the negative control and plotted as histograms (Fig. 2A) as a means to visualize their distribution as a function of the antigen densities used. Indicative of spurious signaling, a substantial proportion of the CD19 BBz CAR- but not RA14 TCR T cells displayed elevated Fura-2 ratio values in the absence of antigen (Fig. 2A, black histograms intersecting with 0 molecules μm^−2^).

As shown in Fig. 2 (A and B, top), the minimum density required for the activation of 60% of the RA14 TCR T cells amounted to 0.005 molecules μm^−2^. Considering an average synaptic area of 120 μm^2^, we concluded that, at least in the initial stage of contact formation, each T cell encountered on average 0.6 ligands. The fact that a sizable proportion of activated RA14 TCR T cells had fluxed calcium almost immediately after making SLB contact rendered it likely that RA14 TCR T cells can in principle respond already to a single antigenic pMHC.

Indicative of a considerably reduced antigen sensitivity, only 35% of the CD19 BBz CAR T cell population became activated when facing CD19 at a density of 10 molecules μm^−2^. For best comparison, we plotted recorded dose-response curves to calculate the ligand density required for the half-maximal response, hereafter termed the activation threshold (for details, refer to Materials and Methods). As is shown in Table 1, the median effective concentration (EC_50_) antigen density for CD19 BBz CAR T cell activation threshold was more than 3000 times higher than that of RA14 TCR T cells. Furthermore, the average intensity of the calcium flux in the activated T cell population as expressed as Fura-2 ratio and depicted in Fig. 2 (A and B, middle) was substantially higher for RA14 T cells than for CD19 BBz CAR T cells. In line with calcium-based analysis of T cell activation, antigen levels for half maximal interferon-γ (IFN-γ) secretion were about 1000 times higher for CD19 BBz CAR T cells when compared to that of RA14 TCR T cells (Fig. 2B, bottom).

**Table 1.** Fitting parameters of the three-parameter dose-response curve fits. Dose-response curves of receptor-modified T cells were fitted with Eq. 1 to extract the antigen density at half-maximum T cell activation (EC_50_). The 95% confidence intervals (CI) are shown. For STAR_mir_ (CRISPR) targeting A2/4D and STAR_mir_ (lentiviral) targeting A2^ΔCD8^/6T, the fit was constraint to top <101. Bottom, top, and EC_50_ values represent antigen molecules per μm^2^. n.d., not determined.

Receptor construct	Ligand	Bottom	Top	EC_50_	Bottom (95% CI)	Top (95% CI)	EC_50_ (95% CI)
RA14 (CRISPR-Cas9)	A2/CMV	−7.841	96.63	0.004486	−28.93 to 13.25	82.68 to 110.6	0.00111 to 0.007863
	A2^ΔCD8^/CMV	2.377	45.66	2.813	−7.519 to 12.27	31.81 to 59.51	0.000 to 8.277
1G4 TCR (CRISPR-Cas9)	A2/9V	−9.813	98.87	0.007032	−24.67 to 4.447	92.17 to 105.6	0.004563 to 0.01121
	A2^ΔCD8^/9V	−3.2 3	93.01	0.0181	−12.80 to 6.047	88.64 to 97.39	0.01254 to 0.02702
1G4hi TCR (CRISPR-Cas9)	A2/9V	−1.813	86.82	0.03997	−14.37 to 10.74	77.16 to 96.49	0.007062 to 0.07288
	A2^ΔCD8^/9V	1.507	75.48	0.07359	−13.95 to 16.96	62.01 to 88.94	0.000 to 0.1672
STARnat (CRISPR-Cas9)	A2/9V	−8.673	83.75	0.01886	−24.64 to 6.063	75.45 to 92.12	0.01010 to 0.03907
	A2^ΔCD8^/9V	−0.4987	87.6	0.01151	−11.60 to 10.27	82.48 to 92.75	0.006219 to 0.01990
	A2/6T	1.9	87.8	0.03644	−25.33 to 27.38	76.33 to 99.35	0.01293 to 0.1572
	A2^ΔCD8^/6T	4.087	89.07	0.01929	−9.497 to 17.12	82.65 to 95.55	0.009965 to 0.03745
	A2/4D	1.17	97.65	51.48	−2.055 to 4.360	79.61 to 123.4	29.20 to 90.77
	A2^ΔCD8^/4D	7.584	82.3	2.66	−0.07432 to 15.07	75.52 to 89.22	1.071 to 5.814
STARmir (CRISPR-Cas9)	A2/9V	−3.489	84.14	0.047	−12.90 to 5.451	78.95 to 89.38	0.03037 to 0.07628
	A2^ΔCD8^/9V	−6.067	90.15	0.01993	−23.55 to 10.82	82.92 to 97.42	0.01154 to 0.03583
	A2/6T	1.583	85.51	0.06927	−9.178 to 11.57	79.89 to 91.14	0.04043 to 0.1349
	A2^ΔCD8^/6T	−0.3774	94.34	0.01701	−11.63 to 10.71	89.27 to 99.43	0.01088 to 0.02690
	A2/4D	3.027	~101.0	111.2	−0.07006 to 6.112	Hit constraint	71.17 to 143.7
	A2^ΔCD8^/4D	4.568	84.35	1.477	−1.216 to 10.27	79.76 to 89.01	0.8369 to 2.599
T1 BBz CAR (CRISPR-Cas9)	A2/9V	14.84	81.87	0.8818	10.81 to 18.87	76.90 to 86.85	0.3809 to 1.383
	A2^ΔCD8^/9V	6.179	58.99	1.887	2.480 to 9.878	55.10 to 62.87	0.6608 to 3.113
	A2/6T	13.68	74.55	5.097	10.26 to 17.11	69.22 to 79.89	2.582 to 7.612
	A2^ΔCD8^/6T	10.79	52.54	3.221	4.915 to 16.67	44.54 to 60.54	0.000 to 7.204
	A2/4D	12.65	76.78	192.2	10.42 to 14.89	65.56 to 88.00	93.62 to 290.8
	A2^ΔCD8^/4D	n.d.	n.d.	n.d.	n.d	n.d.	n.d.
εTRuC (lentiviral)	A2/9V	−3.639	83.79	0.03264	−17.97 to 10.69	74.38 to 93.21	0.007065 to 0.05822
	A2^ΔCD8^/9V	−3.662	88.24	0.03544	−17.30 to 9.974	78.61 to 97.87	0.005046 to 0.06583
	A2/6T	−1.106	95.7	0.02477	−9.644 to 7.432	89.62 to 101.8	0.01401 to 0.03552
	A2^ΔCD8^/6T	−3.717	92	0.02314	−10.77 to 3.331	87.71 to 96.29	0.01466 to 0.03162
	A2/4D	1.828	99.37	23.32	−4.363 to 8.018	85.77 to 113.0	8.600 to 38.04
	A2^ΔCD8^/4D	0.9009	96.92	5.601	−3.259 to 5.061	89.82 to 104.0	3.285 to 7.916
STARnat (lentiviral)	A2/9V	−2.586	80.4	0.02096	−23.92 to 18.75	57.57 to 103.2	0.000 to 0.06173
	A2^ΔCD8^/9V	−1.961	76.69	0.1229	−17.07 to 13.15	58.83 to 94.56	0.000 to 0.2819
	A2/6T	2.77	100.1	0.1028	−14.63 to 20.17	63.68 to 136.5	0.000 to 0.2317
	A2^ΔCD8^/6T	0.3434	84.83	0.2053	−8.949 to 9.636	72.49 to 97.18	0.05478 to 0.3558
	A2/4D	n.d.	n.d.	n.d.	n.d.	n.d.	n.d.
	A2^ΔCD8^/4D	n.d.	n.d.	n.d.	n.d.	n.d.	n.d.
STARmir (lentiviral)	A2/9V	−1.742	90.94	0.1211	−6.789 to 3.306	84.06 to 97.81	0.06364 to 0.1785
	A2^ΔCD8^/9V	0.6705	81.53	0.1716	−9.062 to 10.40	62.12 to 100.9	0.03136 to 0.3117
	A2/6T	5.297	86.94	0.07669	−7.019 to 17.61	62.84 to 111.1	0.000 to 0.1635
	A2^ΔCD8^/6T	−2.142	~101.0	0.3305	−7.634 to 3.351	Hit constraint	0.000 to 0.7585
	A2/4D	3.257	91.62	0.101	−11.07 to 17.59	64.54 to 118.7	0.000 to 0.2128
	A2^ΔCD8^/4D	−0.2105	84.22	0.1449	−17.43 to 17.00	15.34 to 101.0	0.000 to 0.4527
T1 BBz CAR (lentiviral)	A2/9V	10.45	84.55	3.982	3.588 to 17.32	62.26 to 106.8	0.000 to 8.563
	A2^ΔCD8^/9V	12.34	63.26	4.852	6.662 to 18.01	38.23 to 88.29	0.000 to 10.81
	A2/6T	10.68	56.24	10.81	6.635 to 14.73	42.33 to 70.15	0.000 to 24.85
	A2^ΔCD8^/6T	9.735	61.95	1.72	0.1782 to 19.29	36.75 to 87.15	0.000 to 4.765
	A2/4D	n.d.	n.d.	n.d.	n.d.	n.d.	n.d.
	A2^ΔCD8^/4D	n.d.	n.d.	n.d.	n.d.	n.d.	n.d.
**εTRuC CD19** (lentiviral)	CD19	7.296	64.42	0.3078	−1.061 to 14.77	58.64 to 71.03	0.1119 to 0.9657
**CD19 BBz CAR** (lentiviral)	CD19	8.904	62.19	16.17	5.631 to 12.18	53.09 to 71.28	6.779 to 25.56

To identify reasons underlying the sizable differences in antigen sensitivities between RA14 TCR and CD19 BBz CAR T cells, we investigated the role of the CD8 coreceptor, which interacts with antigen-loaded MHC class I (MHCI) molecules but not with CD19. As pointed out above, CD8 is tethered via its cytoplasmic tail to TCR-proximal Lck, which phosphorylates, upon TCR-pMHC engagement, the cytoplasmic ITAMs of the CD3 chains and, in a following step, ITAM-recruited ZAP70 for further downstream signaling. Figure 2 (A and B, top) demonstrates that CD8 engagement with MHCI was paramount to maintaining the exquisite antigen sensitivity of RA14 TCR T cells, as we noticed a 600-fold decrease in antigen sensitivity when stimulating T cells with A2^ΔCD8^/CMV, a mutant (DT227/228KA) with abrogated CD8 binding. Once activated, RA14 TCR T cells outcompeted CD19 BBz CAR T cells also with regard to the amplitude of the calcium response (Fig. 2B, middle). Of note, higher levels of antigen-dependent calcium signaling in RA14 TCR T cells were also dependent on CD8 coreceptor engagement, as abrogation of the CD8 binding site in A2/CMV muted mean Fura-2 ratios to levels that were comparable with those observed in CD19 BBz CAR T cells.

### CRISPR-Cas9–mediated knock-in boosts surface expression of TCR/CD3-based synthetic antigen receptors

We reasoned that the therapeutic use of synthetic antigen receptors, which had been engineered along the evolved TCR/CD3 architecture, may at least in part give rise to the exquisite sensitivity and fidelity which characterize TCR-based antigen recognition. If true, such properties could in principle be exploited to not only enhance clinical efficacy but also to broaden the spectrum of permissible antigen interaction kinetics in efforts to refine tumor specificity. However, such rationale may be derailed by the previously observed tendency of sensitized TCR-based antigen recognition to depend on CD8 or CD4 coengagement, which is, in most cases, not supported by TAAs targeted by CARs. To address such possibility in a comprehensive manner, we dissected the contribution of CD8-mediated antigen engagement to the recognition of an A2-restricted T cell epitope by T cells, which had been modified with cognate TCRs, second-generation CARs, or TCCs (see below).

**Fig. 3.**
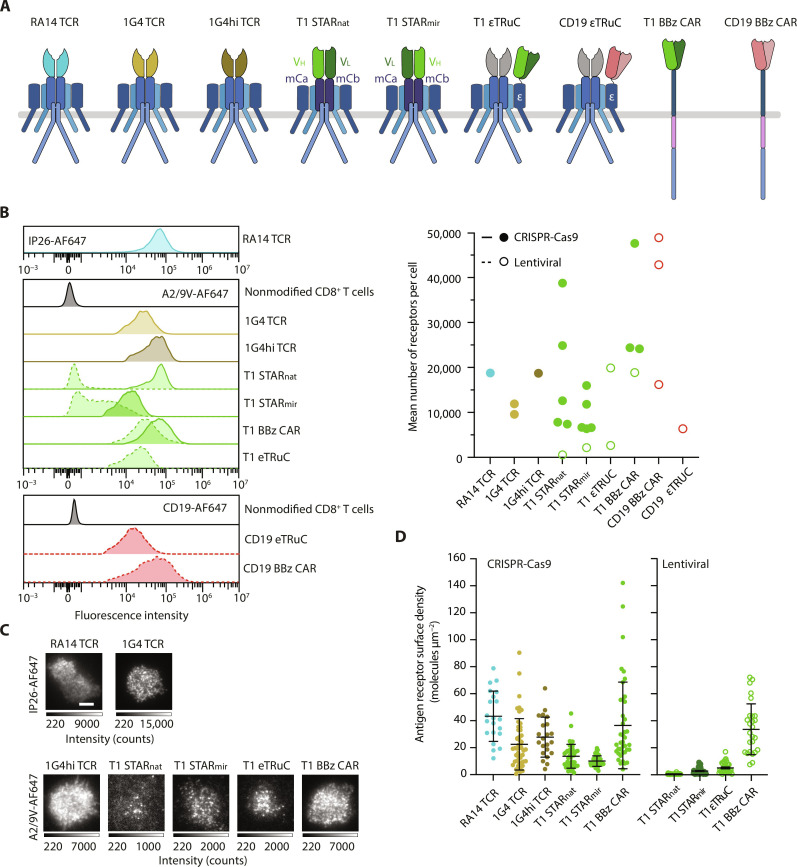
Design and surface expression of synthetic antigen receptor constructs. (**A**) Schematic representation of RA14 TCR, 1G4 TCR, affinity-enhanced c58c61 1G4 TCR (1G4hi TCR), synthetic receptors T1 STAR_nat_ and T1 STAR_mir_ (based on the murine TCRαβ), T1 or FMC63 scF_V_ tethered to CD3ε via a flexible linker (εTRuC), T1-scF_V_ (T1) BBz CAR, and FMC63 scF_V_ (CD19) BBz CAR. (**B**) Left: Histograms depicting flow cytometric surface analysis of CRISPR-Cas9–engineered (solid line) and lentivirally transduced (dashed line) antigen receptor modified T cells as performed with indicated probes (FACS plots, left) to determine the mean number of surface-expressed antigen receptors (dot plot, right). Right: Individual flow cytometric measurements are indicated for receptor modifications involving CRISPR-Cas9–based knock-in (filled circles) and lentiviral transduction (empty circles) of T cells derived from up to four donors. Quantitation of surface receptors was based on label saturation and flow cytometric calibration (fig. S2, C and D). (**C** and **D**) TIRF images of living T cells modified with indicated antigen receptors, stained as indicated with probes under saturating conditions, confronted with ICAM-1–functionalized SLBs and quantitated for respective surface densities. Data represent *n* = 21 to 47 synapses of T cells derived from one donor. Scale bar, 5 μm. Statistics: Mean ± SD.

To be able to quantitate and directly compare the detection capacity of TCR, CAR of TCC-modified T cells, we chose to target the canonical cancer-testis NY-ESO-1 peptide presented in the context of A2 via conventional TCR recognition or with the use of the TCR-like 3M4E5 monoclonal antibody (mAb)–derived T1 scF_V_, serving as the binding module for T1 CARs and T1 TCCs (see below and Fig. 3A) (*34*). The T1 scF_V_ undergoes stable interactions with A2/NY-ESO-1 in a peptide-specific manner, and point mutations within the NY-ESO-1 peptide (e.g., 9V, 6T, and 4D) allowed us to vary receptor antigen binding parameters and assess their role in sensitized ligand recognition [for an overview of T1-scF_V_–binding kinetics with A2/9V, A2/6T, and A2/4D, please refer to fig. S3 (A to C) and table S1]. Using A2/NY-ESO-1 as the model antigen enabled us furthermore to directly compare the functional performance of synthetic antigen receptors with that of the A2/NY-ESO–specific 1G4 TCR and a genetically engineered version thereof, which binds A2/9V with picomolar affinity (c58c61 1G4hi TCR). For direct comparison, we included a second-generation T1-BBz CAR as well as three different T1-TCCs.

As schematically depicted in Fig. 3A, we constructed two STARs, for which we replaced the V_α_ and V_β_ domains of a murine TCR with the V_L_ and V_H_ domains of the T1 scF_V_. STAR natural (T1 STAR_nat_, with V_L_ fused to Cβ and V_H_ to Cα) denotes a version which engages A2/NY-ESO-1 in a natural docking angle, as is observed for the large majority of productive TCR-pMHC interactions (*34*) and which preserves the orientation of CD8 binding with regard to STAR engagement. The second STAR construct termed STAR mirror (T1 STAR_mir_, with V_H_ tethered to Cβ and V_L_ to Cα) binds A2/NY-ESO-1 in the opposite orientation (*34*). As an alternative to STARs, we linked the T1-scF_V_ via a flexible linker to the N terminus of CD3ε to result in a so-termed CD3ε TRuC (ε) after its inclusion into a nascent TCR/CD3 complex.

For ectopic expression, we applied two strategies: (i) orthotopic exchange of the endogenous TCRs via CRISPR-Cas9–mediated knock-in as well as (ii) lentiviral expression. We generated wild-type and high-affinity 1G4 TCR T cells by knocking the α and β subunits of the 1G4 TCR into the *TRAC* locus of human primary CD8^+^ T cells. In an analogous fashion, we introduced both T1 STAR versions and the T1 CAR (fig. S1A) into the *TRAC* locus yet also applied lentiviral expression of these constructs as an alternative and for functional comparison (Fig. 3B). We refrained from knocking-in scF_V_-tethered CD3ε into the genomic CD3ε locus and only resorted to lentiviral transduction to preserve expression of wild-type CD3ε for its inclusion into the nascent TRuC.

The high-affinity antigen interactions between FMC63 and T1 scF_V_s and their corresponding ligands allowed for quantitative surface staining via recombinant monomeric ligands, such as soluble Alexa Fluor 647 (AF647)–conjugated CD19 and A2/9V, respectively. The TCR-reactive mAb IP26 was used to stain surface-accessible RA14 and 1G4 TCRs. In view of picomolar affinities, 1G4hi TCR T cells could also be quantitatively labeled with AF647-conjugated A2/9V. To arrive at absolute numbers of T cell surface–resident receptors, we calibrated recorded mean fluorescence intensities with the use of Quantum molecules of equivalent soluble fluorochrome (MESF; fig. S2, C and D). As shown in Fig. 3B, 1G4 and RA14 T cells expressed as determined with the use of the mAb IP26, on average, between 11,000 and 18,000 TCRs per cell, respectively, i.e., at levels that were similar to those of 1G4hi T cells stained with A2/9V (18,000 TCRs).

Given the empiric nature of their structural design, none of the T1-based antigen receptors used in this study had undergone—unlike TCR/CD3—natural selection with possible consequences for surface expression levels and for the efficacy of T cell antigen recognition. As shown in Fig. 3B, CRISPR-Cas9–mediated knock-in of T1 STAR_nat_, T1 STAR_mir_, and T1 BBz CAR yielded about 18,000, 9000, and 32,000 molecules per T cell, while lentiviral transduction amounted to only 500, 2000, and 18,000 receptor entities per T cell, respectively. In contrast, lentivirally encoded CD19 BBz CAR gave, on average, rise to 36,000 molecules per T cell. In case of lentiviral transduction with the T1-CD3ε fusion construct, average T1-εTRuC surface expression varied, depending on the virus and donor PBMC batch, between 2700 and 20,000 copies per T cell (Fig. 3B).

We also noticed that CRISPR-Cas9–mediated ablation of TCRα and TCRβ genes before lentiviral transduction led to a considerable increase in surface expression of both STAR constructs (fig. S4, A to D). Together, these quantifications support the notion that TCC assembly in the endoplasmic reticulum (ER) and subsequent surface expression is more efficient in the absence of endogenous TCRα and TCRβ subunits. Moreover, placing CAR constructs under the direct transcriptional control of the TCRα promoter boosted expression, in some cases, considerably when compared to randomized lentiviral genome insertion. In accordance with flow cytometric analysis and as shown in Fig. 3 (C and D), direct measurements of surface receptor densities via TIRF microscopy mirrored the flow cytometrically established ranking of expression levels.

### T1-based TCR/CD3-based synthetic receptors confer a superior sensitivity toward ligands featuring a wider range of affinities

**Fig. 4.**
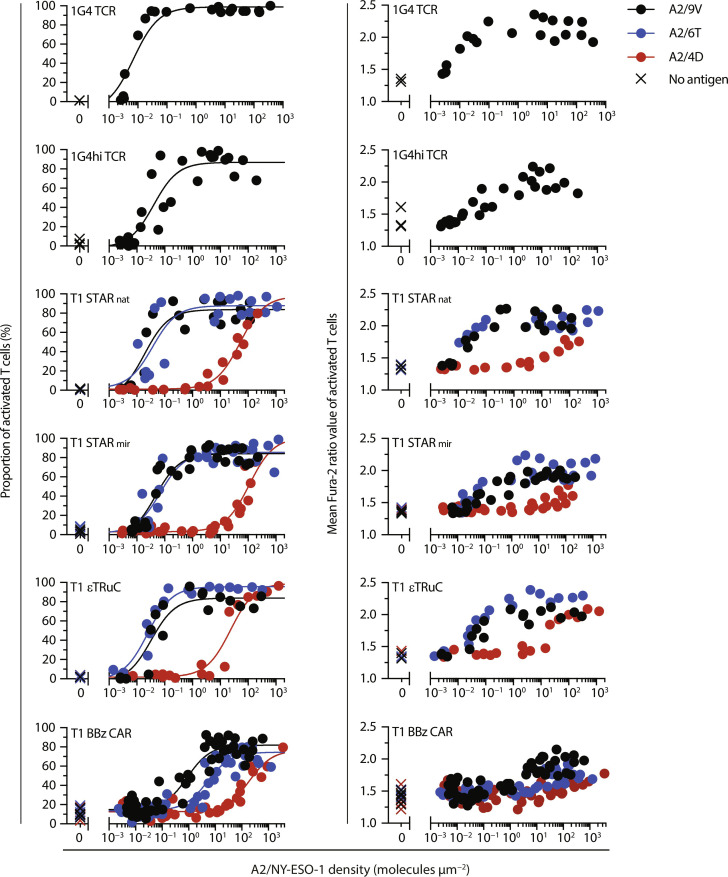
Calcium response of T cells equipped with NY-ESO-1–specific TCR, TCC, or CARs targeting peptide-loaded HLA.A2. T cells modified with a knocked-in 1G4 TCR, 1G4hi TCR, T1 STAR_nat_, T1 STAR_mir_, T1 BBz CAR, or with a lentivirally transduced T1 εTRuC were confronted with SLBs functionalized with A2/9V, A2/6T, and A2/4D at indicated densities. Antigen dose-calcium response (**left**) and mean Fura-2 ratio values of activated T cells (**right**) are shown (refer to fig. S5 for histogram-based analysis). Data were fitted to a three-parameter dose-response curve according to Eq. 1 to extract EC_50_ values and confidence intervals as summarized in Table 1. Data were pooled from *n* = 2 to 3 biological replicates of two to three donors.

**Fig. 5.**
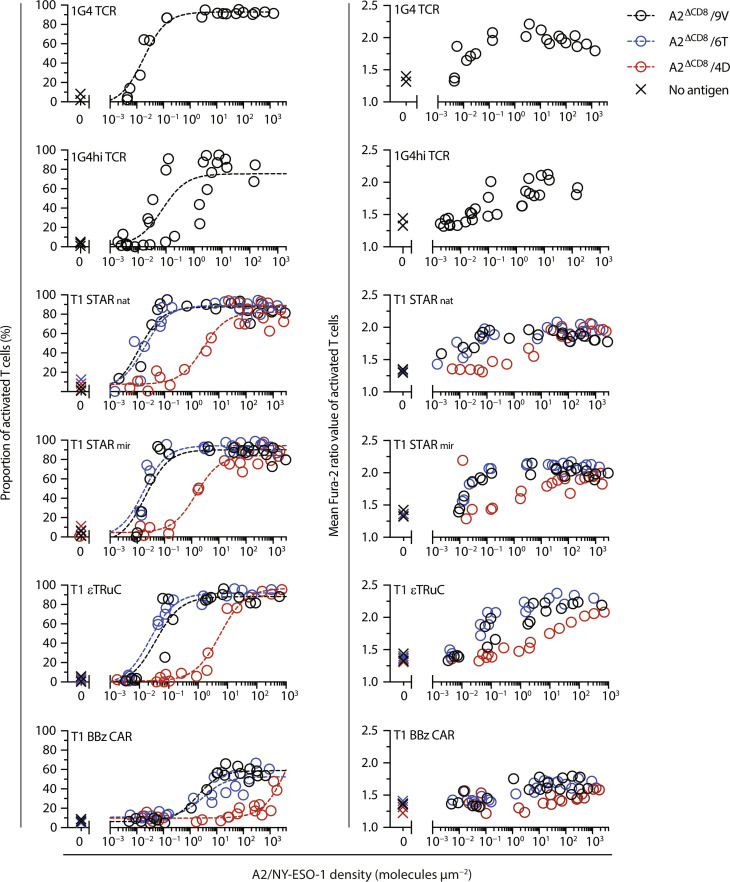
Calcium response of T cells equipped with NY-ESO-1–specific TCR, TCC, or CARs targeting peptide-loaded CD8 binding–deficient HLA.A2^ΔCD8^. T cells modified with a knocked-in 1G4 TCR, 1G4hi TCR, T1 STAR_nat_, T1 STAR_mir_, T1 BBz CAR, or with a lentivirally transduced T1 εTRuC were confronted with SLBs functionalized with A2^ΔCD8^/9V, A2^ΔCD8^/6T, and A2^ΔCD8^/4D at indicated densities. Antigen dose-calcium response (**left**) and mean Fura-2 ratio values of activated T cells (**right**) are shown (refer to fig. S5 for histogram-based analysis). Data were fitted to a three-parameter dose-response curve according to Eq. 1 to extract EC_50_ values and confidence intervals as summarized in Table 1. Data were pooled from *n* = 2 to 3 biological replicates of two to three donors.

We proceeded to assess the sensitivities toward antigen as they are conveyed by TCCs, CARs, and TCRs, the latter serving as benchmark for comparison (Figs. 4 and 5 and fig. S5). To this end, we confronted receptor-modified T cells with SLBs functionalized with ICAM-1 as well as titrated densities of antigens and determined respective antigen thresholds for activation by monitoring changes in intracellular calcium levels. Our analysis encompassed the full spectrum of available antigens, including wild-type and CD8 binding–deficient mutant versions of A2 complexed with high-affinity 9V, intermediate-affinity 6T, or low-affinity 4D variants of the NY-ESO-1 peptide. Given their comparatively higher receptor expression levels (Fig. 3, B to D), we primarily focused our analyses on CRISPR-Cas9–engineered 1G4, 1G4hi, T1 STAR_nat_, T1 STAR_mir_, and T1 BBz CAR T cells. As mentioned above, we applied lentivirally transduced T1 εTRuC T cells to maintain expression of wild-type CD3ε.

**Fig. 6.**
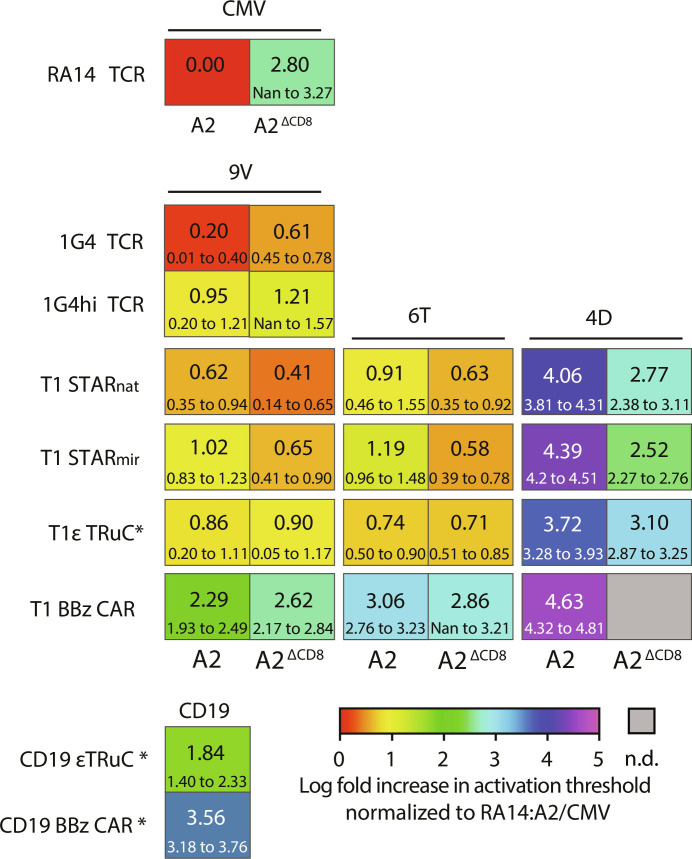
Comparative heatmap of T cell antigen sensitivities conveyed by antigen receptors. Log fold increase in antigen threshold (EC_50_ value) required for T cell activation (normalized to the activation threshold of A2/CMV-confronted RA14 T cells) for indicated receptor constructs and ligands. Numbers in smaller font indicate the bottom and top 95% confidence intervals of the EC_50_ value normalized to the EC_50_ value of the RA14 T cells. Antigen receptor–expressing T cells marked with an asterisk (*) were lentivirally transduced. All other constructs were introduced via CRISPR-Cas9. Nan, not a number; n.d., not determined.

As illustrated by antigen dose-response curves in Fig. 4, represented in Fig. 6, and tabulated in Table 1, 1G4 T cells demonstrated single-molecule sensitivity toward A2/9V, surpassing 1G4hi T cells by a factor of 5.

Among TCC- and CAR-modified T cells, STAR_nat_ T cells exhibited superior antigen sensitivities, when confronted with the high-affinity A2/9V ligand [half-life (*t*_1/2_)_, 37°C_ = 150 s, dissociation constant (*K*_D_) = 19.8 nM]. They required only two to three times higher antigen levels compared to 1G4 T cells and demonstrated a 50-fold higher sensitivity than the trailing T1 BBz CAR (Figs. 4 and 6 and Table 1). Notably, amplitudes of the calcium flux were considerably higher in T1 TCC T cells compared to T1 CAR T cells, especially when T cells were scanning antigens present at lower densities, closely resembling amplitudes observed in antigen-stimulated 1G4 T cells (Fig. 4, right).

We next investigated whether and to what extent antigen receptor binding kinetics differentially affected antigen detection via TCCs and CARs. When faced with the intermediate-affinity A2/6T ligand (*t*_1/2, 37°C_ = 20 s, *K*_D_ = 217 nM; Fig. 4 and fig. S3C), T1 TCC T cells recognized A2/6T with similar if not identical efficiencies compared to the high-affinity A2/9V. They also outperformed T1 CAR T cells decisively with regard to antigen thresholds (by 200-fold) and also in terms of the average calcium signal amplitude. Only when confronted with the low-affinity ligand A2/4D (*t*_1/2, 37°C_ = 2.97 s, *K*_D_ = 2.97 μM), T1 TCC T cells displayed a substantially diminished recognition efficiency, as they required the presence of more than 5000 ligands under the T cell synapse for activation, which was moreover associated with a considerably reduced calcium signal amplitude (Figs. 4 and 6).

As witnessed above, sensitized TCR-mediated antigen detection is often critically dependent on the interaction of the coreceptor CD8 with MHCI molecules. We assessed the involvement of CD8 in antigen recognition by exposing T cells to peptide-loaded, CD8 binding–deficient A2^ΔCD8^ mutant versions. While A2/CMV recognition by RA14 T cells was more than 600 times sensitized by CD8-MHCI binding (Figs. 2B and 6), only a moderate (~2-fold) improvement in A2/9V detection was observed in both 1G4 and 1G4hi T cells. Similar trends were observed with T1 TCC and CAR T cells recognizing various versions of the NY-ESO-1 peptide in the context of A2 or A2^ΔCD8^. Allowing for CD8-MHCI binding to occur promoted antigen sensitivity—if at all—only to a modest extent (maximally sixfold), which still fell within the overlapping confidence intervals of measured antigen sensitivities for T1 TCC- and BBz CAR T cells.

We also investigated the impact of lentiviral transduction on antigen recognition performance of T1-TCCs and T1 BBz CARs. We considered it possible that knock-in–based gene introduction via CRISPR-Cas9 not only elevated antigen receptor expression levels but also altered membrane-proximal and membrane-distal TCR signaling in the absence of endogenous TCR/CD3. Despite reduced antigen receptor expression levels, TCC-expressing T cells exhibited only a moderate (up to 2.5-fold) reduction in sensitivity toward A2/9V compared to those obtained with knocked-in TCCs (fig. S6 and Table 1). Also, the amplitude of the calcium flux was—if at all—only minimally affected by the mode of gene delivery (fig. S6, right). However, T cells expressing knocked-in T1 BBz CAR demonstrated a fivefold improvement in detection performance compared to their lentivirally transduced counterparts.

**Fig. 7.**
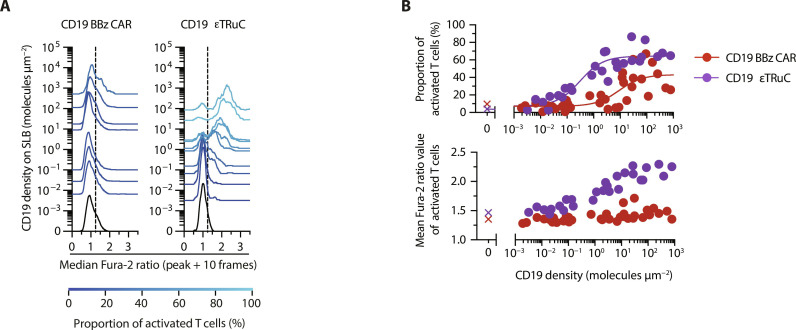
Calcium response of T cells equipped with a CD19-reactive second-generation CAR or an εTRuC. (**A**) Histograms depicting population-wide analysis of the calcium response of CD19 BBz CAR- and CD19 εTRuC T cells confronted with SLBs functionalized with CD19 at indicated densities as well as ICAM-1. Dashed lines indicate the Fura-2 ratio threshold above which T cells were considered activated. (**B**) Top: Rendering of data shown in (A) as antigen dose-response curves. Data were fitted to Eq. 1 as summarized in Table 1 (only εTRUC shown). Bottom: Mean Fura-2 ratio values measured for the activated fraction of CD19 BBz CAR- and CD19 εTRuC T cells [see (A)] confronted with SLBs featuring CD19 at indicated densities. Data were pooled from *n* = 3 biological replicates of three donors.

In line with above findings related to the performance of CD19 BBz CAR T cells, T1 BBz CAR T cells exhibited also elevated calcium signaling in the absence of antigen, regardless of the mode of antigen receptor gene delivery (lentiviral or knock-in). In contrast, T cells modified with 1G4 or 1G4hi TCRs, as well as T1 TCCs, did not show any evidence for this behavior (figs. S5 to S7). Of note, for CD19 εTRuC–modified T cells, we also witnessed the same signature of enhanced antigen sensitivity in the absence of spurious activation (Figs. 6 and 7, A and B). We hence conclude that TCCs maintain the fidelity of activation, a hallmark of conventional T cells, while outperforming conventional second-generation CARs with regard to antigen sensitivity by up to three orders of magnitude.

Of note, as shown in fig. S8 (A and B), IFN-γ release by TCC-modified T cells increased in an antigen density–dependent manner. In contrast and regardless of the mode of genetic transfer, T1 BBz CAR T cells secreted considerable IFN-γ levels already in the absence of antigen, which rose marginally even at higher antigen levels and amounted to only twice to three times the background levels when CAR T cells had been confronted with high antigen densities.

### TCCs but not second-generation CARs promote CTL-mediated target cell killing at low antigen levels

We next investigated the efficacy of synthetic receptors in inducing target cell cytolysis in response to antigen stimulation. Using the T1 system, we incrementally increased antigen densities by titrating the NY-ESO-1 peptide in the target cell culture. We used HLA.A2-expressing K562 cells pulsed with 9V peptide as target cells for cytolysis assays. Initially, we used T cells expressing low levels of T1 BBz CAR, T1 STAR_nat_, T1 STAR_mir_, or T1 εTRuC via lentiviral transduction as effector cells, with 1G4 T cells serving as a reference. Target cell lysis was quantitated after 6, 8, and 24 hours of coculture at a 1:1 ratio of target to effector cells.

**Fig. 8.**
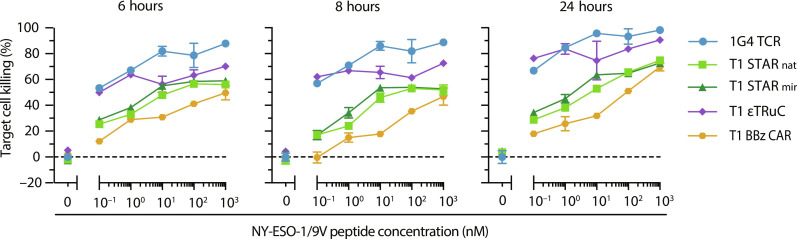
Ex vivo cytotoxic capacity of engineered T cells with low antigen receptor expression levels. 1G4 T cells were engineered via CRISPR-Cas9; all other constructs were lentivirally transduced [refer to Fig. 3 (B to D)]. Effector cells were cocultured for indicated times at a ratio of 1:1 and with HLA.A2-, CD80-, and luciferase-expressing K562 feeder cells, prepulsed at specified concentrations with the 9V NY-ESO-1 peptide derivative. Statistics: Mean ± SEM from technical duplicates of one donor.

As depicted in Fig. 8, 1G4 TCR T cells exhibited the highest degree of killing, particularly at the lowest 9V peptide concentration (0.1 nM) applied for pulsing (40, 65, and 65% killing after 6, 8, and 24 hours, respectively). The cytotoxic response of T1 εTRuC T cells was similarly sensitive (at 0.1 nM 9V peptide: 37, 63, and 67% killing after 6, 8, and 24 hours, respectively), albeit less pronounced at higher 9V peptide concentrations, even after 24 hours of coculture. This reduced response is likely attributed to the substantially higher affinity of T1-A2/9V, which may interfere with synapse dissolution and serial killing as a consequence.

At low antigen densities, T cells engineered with T1 STAR_nat_ (19, 32, and 42% killing after 6, 8, and 24 hours, respectively) and T1 STAR_mir_ (20, 29, and 44% killing after 6, 8, and 24 hours, respectively) exhibited indistinguishable cytolytic responses, which scored, however, substantially lower than those induced by 1G4 TCR- and T1 εTRuC-modified T cells. Target cell lysis increased gradually with rising antigen levels and duration of coculture, saturating at levels comparable to those induced by 1G4 TCR T cells at 9V peptide concentrations of 10 nM and higher.

Consistent with calcium- and IFN-γ–based assays of T cell responsiveness, T1 BBz CAR T cells showed by far the least efficient cytolytic response when confronted with target cells pulsed with the lowest 9V peptide concentration (0.1 nM). Target cell killing was in fact indistinguishable from that of the negative control measured 6 and 8 hours after cell pooling. After 24 hours of coculture, specific cytolysis increased to 6% after subtraction of background activity. Only at the highest antigen dose applied (1 μM), target cell killing approached levels conferred by T cells engineered with the 1G4 TCR or TCR/CD3-based synthetic antigen receptor constructs.

Notably, when using T cells with substantially higher TCC or CAR expression, as achieved through CRISPR-Cas9–mediated receptor knock-in or by selecting high lentiviral T1 εTRuC expressors, we observed a considerable increase in nonspecific target cell lysis in the absence of 9V peptide (fig. S9). This observation can be attributed to residual weak binding of the T1-scF_V_ to endogenously peptide-loaded A2, which is absent on SLBs but abundant on target cell surfaces (*35*). Our findings underscore the need for synthetic fine-tuning of the T1-based system to achieve maximal peptide specificity as a necessity for any clinical application of TCC T cells featuring enhanced antigen detection. In conclusion, TCRs and TCCs outperform conventional second-generation CARs not only with regard to sensitized calcium-based activation and IFN-γ secretion but also target cell killing.

### TCCs convey superior membrane-proximal signal transmission

T cell antigen recognition is intimately tied to synaptic receptor antigen binding events. As outlined above, these become transmitted across the plasma membrane by means of Lck-mediated ITAM phosphorylation which is followed by ZAP70’s recruitment to and activation at phospho-ITAMs for further amplification of downstream signaling. In view of the considerable differences in antigen detection capacities we had observed above, we sought to juxtapose signaling activities immediately downstream of T1 STAR_nat_, T1 STAR_mir_, T1 εTRuC, and the second-generation T1 BBz and CD19 BBz CARs. We included in our analysis RA14 TCR T cells confronted with A2/CMV, serving as a benchmark for single-molecule antigen sensitivity.

**Fig. 9.**
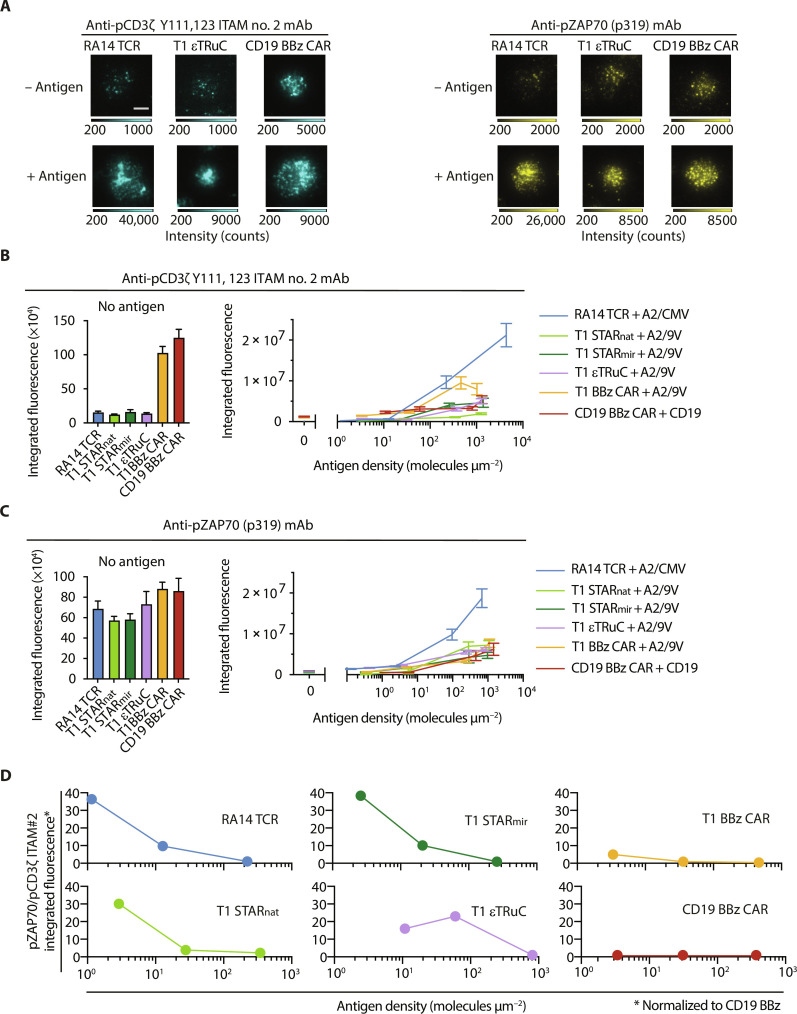
Immunofluorescence-based analysis of synapse-associated CD3ζ pITAM#2 and pZAP70. (**A**) Synaptic immunofluorescence recorded in TIRF mode with the use of indicated antibodies. Shown are synapses of T cells modified with indicated antigen receptors and in contact with SLBs featuring ICAM-1 and, if indicated, their nominal antigen (A2/CMV, A2/9V, and CD19, respectively). (**B**) Synapse-associated integrated fluorescence of CD3ζ Y111, 123 ITAM no. 2 in the absence (left) and presence of antigen (right) as indicated. Data are representative of *n* = 20 to 48 recorded T cell synapses per condition of one donor. Statistics: Mean ± SEM. (**C**) Synapse-associated integrated fluorescence of ZAP70 pY319 in the absence (left) and presence of antigen (right) as indicated. Data are representative of *n* = 21 to 60 recorded T cell synapses per condition of one donor. Statistics: Mean ± SEM. (**D**) Ratios of pZAP70 and pITAM#2 integrated fluorescence intensities [from (B) and (C)] which had been normalized to ratio values measured for CD19 BBz CAR T cells. For analysis, integrated pZAP70 fluorescence values had been extrapolated for antigen densities associated with measured integrated pITAM#2 fluorescence values.

To assess membrane-proximal downstream signaling, we first quantitated ITAM and ZAP70 phosphorylation levels in T cells responding to SLB functionalized with titrated antigen densities. To this end, receptor-modified T cells confronted with stimulatory SLBs were fixed and subjected to membrane permeabilization. Relative levels of CD3ζ ITAM#2 and ZAP70 phosphorylation were then determined by TIRF microscopy with the use of fluorescence-tagged specific mAbs (Fig. 9A, left). We targeted phosphorylated CD3ζ ITAM#2 since the resulting immune fluorescence gave rise to the best signal-to-noise ratio when compared to the fluorescence obtained via CD3ζ pITAM#1- and pITAM#3-specific mAbs (*13*). Levels of activated ZAP70 were monitored with the use of a fluorescence-labeled mAb specific for phosphorylated ZAP70 (pY319) (Fig. 9A, right) (for further details, please refer to Materials and Methods).

We noticed for both T1 BBz– and CD19 BBz–modified T cells anti-CD3ζ pITAM#2 fluorescence signals in the absence of antigenic ligands, which amounted to levels that were up to 10 times increased when compared to those obtained from all other T cell lines analyzed (Fig. 9B, left). These observations are in good agreement with spurious calcium signaling we had recorded in second-generation CAR T cells but not in T cells modified with TCRs or TCCs (Figs. 2, A and B, and 4 and figs. S5 to S7).

For RA14 TCR, T1 STAR_nat_, T1 STAR_mir_, and T1 εTRuC T cells, we failed to detect a measurable increase in CD3ζ ITAM#2 phosphorylation above background levels at antigen densities that were slightly above the thresholds for activation, i.e., sufficient to activate 60 to 90% of the SLB-contacting T cells (Figs. 2 and 4, figs. S5 to S7, and Table 1). We only noticed a small increase in fluorescence signals for T cells facing moderate antigen levels well above antigen thresholds. This implied that the number of triggered antigen receptors required to initiate full T cell activation was substantially lower than the number of antigen receptors that were already partially phosphorylated in the absence of antigen. In contrast, at antigen levels well above thresholds for activation, we noticed a gradual rise in synaptic anti-CD3ζ pITAM#2 fluorescence for all T cell lines analyzed. Yet in view of (i) the seemingly low number of triggered TCR/CD3-based constructs required to promote full T cell activation and also because of (ii) the up to 70-fold differences in ectopic receptor expression levels (Fig. 3, B to D), we reasoned that measured anti-CD3ζ pITAM#2 fluorescence intensities hardly constituted a reliable parameter for meaningful comparisons of antigen receptor–proximal signaling output (see below). We considered them instead to be of analytic value only when viewed in the context of subsequent events in canonical TCR signal transduction, in particular the recruitment and activation of ZAP70 to pITAMs of triggered antigen receptors.

Unlike the levels of synaptic CD3ζ pITAM#2 recorded in the absence of antigen, corresponding synapse-associated pZAP70 levels were only moderately higher in T1 or CD19 BBz CAR T cells than in RA14 TCR, T1 STAR_nat_, T1 STAR_mir_, and T1 εTRuC T cells (Fig. 9C). We hence inferred that the 7- to 10-fold higher CD3ζ pITAM#2 background levels associated with CAR T cells (Fig. 9B) resulted only in a 1.3- to 1.5-fold higher degree of ZAP70 activation. Such behavior would be consistent with a substantially weaker link between ITAM phosphorylation and ZAP70 activation in second-generation CAR T cells yet might also explain elevated calcium levels observed in the absence of antigen.

To gauge membrane-proximal amplification of downstream signaling in a fashion that was no longer compromised by varying levels of ectopic receptor expression, we determined the efficiency with which ZAP70 had become activated as a function of ITAM phosphorylation. For this, we divided synaptic anti-pZAP70 intensity values by anti-pITAM#2 fluorescence intensity values (Fig. 9D). Consistent with their extraordinary antigen sensitivity, RA14 TCR T cells, which benchmarked most efficient activation, exhibited the highest ratio, especially at low antigen densities, followed by T1 STAR_nat_, T1 STAR_mir_-, and T1 εTRuC T cells. Indicative of their limited detection capacities, CD19 and T1 BBz CAR T cells showed by far the lowest pZAP70:pITAM#2 ratios at all antigen densities analyzed.

We hence conclude that canonical progression from ITAM phosphorylation to ZAP70 recruitment and phosphorylation occurs at a considerably higher frequency in ultrasensitive RA14 TCR T cells than in CD19 or T1 BBz CAR T cells, especially when antigen is present in low abundance. Of note, TCC T cells outperformed second-generation CAR T cells at all antigen densities tested.

## DISCUSSION

Empiric CAR designs elicit potent antitumor T cell activity yet often fail to support long-term tumor remission due to (i) an inadequate sensitivity they convey toward TAAs and (ii) also the limited persistence of CAR T cell effector functions. Past efforts to address these shortcomings have rarely questioned the operational fitness of conventional CAR architectures but have instead hinged in large part on modifying or adding CAR modules (*26*–*29*, *36*, *37*). Furthermore, receptor-ligand affinities, CAR stoichiometry, and surface expression have been fine-tuned in previous attempts to boost overall performance.

Here, we deliberately abstained from reesngineering onedimensional CAR constructs. Our antigen receptor designs were instead guided by the rationale to emulate the unmatched fidelity and detection efficacy of conventional T cells through the evolved TCR/CD3 architecture and its coevolved signaling machinery. We reasoned that such hallmarks of antigen detection are at least in part conserved in TCR/CD3-based receptor systems. Despite observing lower surface expression levels of some TCR/CD3-based receptor constructs, such as STAR_nat_ and STAR_mir_, even after their CRISPR-Cas9–mediated knock-in into the TCRα locus, the enhanced functional performance of these constructs often rivaled that of endogenous or ectopically expressed TCR/CD3 complexes, validating our initial assumptions.

Our findings were consistent with previous reports (*28*, *29*, *36*, *37*) yet yielded, in addition, precise activation thresholds to be ranked with respect to the ultimate benchmark of a single antigen per synapse, as set by CMV-specific RA14 TCR and to a slightly lesser extent also by NY-ESO-1–specific 1G4 TCR T cells. By focusing on A2/NY-ESO-1 as a molecular target recognized through (i) TCRs, (ii) TCCs, or (iii) a CAR, we overcame complications arising from the divergent nature of epitopes recognized via TCR- and CAR-modified T cells, as well as sensitization of antigen recognition by coreceptor engagement. In addition, automated image recording and processing facilitated single-cell–based analysis of thousands of cells with single-molecule resolution.

Notably, all TCCs supported high sensitivity toward antigens with high (A2/9V) and intermediate (A2/6T) affinities, outperforming corresponding second-generation CARs in both, antigen thresholds and the quality of downstream calcium signaling. However, all T cells equipped with T1-based synthetic antigen receptors exhibited limitations in detecting A2/4D, a ligand with a T1 scF_V_-binding dynamics resembling that of the large majority of agonist pMHC-TCR interactions. Similarities and differences in ligand responsiveness between TCR- and TCC-modified T cells suggest that they share many but clearly not all mechanisms underlying receptor-triggering and membrane-proximal signaling.

We chose to work with both T1 STAR_nat_ and T1 STAR_mir_ constructs in view of the anisotropic nature of ligand engagement by TCRs, which are embedded for reasons of surface expression and signaling within the evolutionarily conserved architecture of the TCR/CD3 complex (*18*, *38*). This orientation-selective mode of antigen engagement likely reflects specific geometric constraints which also accommodate coreceptor engagement of the MHC and govern the extraordinary TCR/CD3 signaling capacity (*22*, *38*, *39*). Consistent with this view is our observation that T1 STAR_nat_ T cells surpassed T1 STAR_mir_ T cells with regard to the recognition of A2/9V and A2/6T. On the other hand, the rather moderate nature of this effect (2- to 2.5-fold) suggests that despite supporting exquisite antigen sensitivity, productive ligand engagement by TCCs is subjected to comparatively fewer geometrical constraints.

One consideration in our study related to the overall lengths of complexes formed between the antigen receptors—such as CAR, TRuC, STAR, and TCR—and their ligand A2/NY-ESO-1 within the confined space of the immunological synapse. As evidenced in numerous studies (*40*–*43*) and further elaborated by the kinetic segregation model of T cell activation (*44*), excluding the bulky CD45 transmembrane phosphatase from the considerably smaller antigen-engaged receptors through sheer size differences is essential for effective ITAM phosphorylation and sustained downstream signaling. Our choice to work with the T1 system was influenced by the nearly identical length dimensions shared among all applied synthetic constructs and TCRs. This virtually ruled out differences in kinetic segregation as the cause for the substantial variations in antigen sensitivities which we observed among various receptor-modified T cell lines.

Unlike what has been described for the recognition of pMHC class complexes by most CD8^+^ T cells, the sensitizing effect of CD8 coreceptor engagement proved in our hands only minor for T cells modified with TCCs. However, none of the T cell lines modified with TCCs approached the sensitivity benchmark set by RA14 TCR T cells. There is mounting evidence that downstream signaling resulting from more stable TCR-antigen interactions with off-rates similar to those of T1-A2/9V and T1-A2/6T binding is less dependent on CD8-MHCI binding (*45*, *46*). Alternatively, CD8-mediated sensitization may rest on the specific geometric framework, which may be defined by simultaneous binding of pMHCs with CD8 and TCR/CD3 and which may not be fully supported by T1 STAR_nat_, T1 STAR_mir_, and T1 εTRuC.

Of note, mechanisms underlying αβTCR-mediated T cell activation appear to serve in particular the detection of low-affinity pMHC antigens present in low abundance and outnumbered by structurally similar yet nonstimulatory pMHCs. In contrast, the related γδTCRs, B cells, and Fc receptors act all in a coreceptor-independent fashion: Unlike αβTCRs, they appear best suited for the recognition of more abundant ligands which feature higher affinities and demand in turn—to maintain a high fidelity of detection—a lesser degree of discrimination from other proteins (*47*, *48*). The fact that TAA recognition by TCC formats is almost insensitive to CD8 coengagement places them closer to γδTCRs. Functional detachedness from CD8 binding qualifies TCCs for their use in advanced antitumor therapies targeting low or heterogeneously expressed TAAs other than peptide-loaded MHCI molecules.

We cannot fully exclude the possibility that some of the observed differences in antigen sensitivity, particularly between RA14 TCR and TCC-modified T cells, may relate at least in part to inferior TCC expression. After all, lower TCC surface densities may not only affect the rate of receptor removal from the T cell plasma membrane through extracellular vesicles (*49*) followed by clathrin-mediated internalization (*50*). There is well-documented evidence that TCR exocytosis influences serial target cell killing and signal termination (*51*). Recent findings also suggest that the T1-CAR is released in the form of extracellular vesicles, likely due to CAR-proximal intracellular signaling (*52*). It is hence conceivable that low TCC expression negatively affected signaling, in particular in relation to reduced TCC shedding and internalization, restricting in such fashion the sensitivity of TCC-modified T cells to the levels observed.

We also investigated the potential effects of knocking-in TCC into the TRAC locus to avoid competition of STAR and endogenous TCR chains for CD3 subunits during TCC assembly and to prevent STAR:TCR mispairing. Although we observed elevated surface expression levels for knocked-in STAR_nat_ and STAR_mir_ (when compared to lentivirally expression), we noted only marginal improvements in the recognition of high- and medium-affinity ligands (A2/9V and A2/6T, respectively).

Unlike CARs, TCCs did not give rise to antigen-independent signaling when expressed in T cells. In principle, all domains of the CAR can contribute to spurious activation. Replacing the CD28-derived costimulatory domain with that of 4-1BB was reported to reduce tonic signaling (*11*), yet even 20% of T cells modified with 4-1BB–based CARs displayed elevated intracellular calcium levels in the absence of antigen, a behavior we did not observe in T cells engineered with TCCs. Another reason underlying antigen-independent signaling in CAR T cells may involve cluster formation of CAR molecules induced by dimerization or oligomerization of CAR-associated scF_V_. Because of the lack of constant domains and their stabilizing interdomain disulfide bonds, a diminished structural integrity of V_H_ and V_L_ domain may promote in many scF_V_s the formation of interchain V_H_-V_L_ pairing and—as a consequence—daisy-chained CAR entities. These may undergo spontaneous phosphorylation in the absence of any antigen-mediated trigger. Of note, all TCCs used in our study featured a scF_V_-based antigen-targeting domain as well but did not give rise to spurious signaling. Extensive ER quality control during TCR/CD3 assembly (*19*) may be reflective of substantial evolutionary pressure to preclude spurious signaling and to allow in turn for sensitized detection of single antigens. In support of this notion is the observed recruitment of the inhibitory kinase Csk via an intracellular CD3ε motif (*53*). When incorporated into the cytoplasmic tail of a 28zCAR, this short peptide sequence appeared to reduce spurious activation (*53*). Of note, a recent study by Love and colleagues (*54*) invoked an unexpectedly dominant role CD3ζ-ITAMs in the ability of T cells to efficiently discriminate between high- and low-quality TCR ligands, a feature that is entirely absent in conventional CAR constructs.

Large variations in receptor expression levels in our study together with the considerable degree of antigen-independent CD3ζ ITAM background phosphorylation complicated the immunofluorescence-based identification of membrane-proximal mechanisms, which underlie the observed functional differences between TCRs, CARs, and TCCs. Of note, at antigen levels that were close to activation thresholds, we failed to observe in any of the investigated receptor-engineered T cells an increase in ζITAM phosphorylation above background levels. We hence surmise that antigen-induced phosphorylation of only few antigen receptors leads to the recruitment and activation of ZAP70 in numbers that are sufficient for robust T cell activation. For this to occur, we propose that the phosphorylation status of individual antigen-triggered antigen receptors—naturally evolved TCR/CD3 complexes or synthetic receptors—exceeds substantially that of the highly abundant yet only partially phosphorylated receptor entities, as they continue to emerge from random Lck interactions in the absence of antigen.

In response to low antigen densities (10 or fewer antigens per square micrometer), synaptic ITAM phosphorylation scored substantially higher in CAR T cells than in RA14 TCR T cells. However, only RA14 TCR but not CAR T cells showed synaptic ZAP70 phosphorylation above background at these same antigen densities. Ratiometric analyses of anti-pITAM and anti-pZAP70 immunofluorescence intensities imply that antigen-driven progression from ITAM phosphorylation to ZAP70 activation is markedly reduced in CAR T cells. In the absence of direct evidence, we hypothesize that complete phosphorylation of all available ITAMs within a TCR/CD3 complex, a TCC or a CAR, is critical for effective ZAP70 activation and further downstream signaling. This notion is supported by the observation that ZAP phosphorylation depends on stable ZAP70 association with the cytoplasmic tails of CD3, which relies in turn on the dynamic engagement of both ZAP70-SH2 domains with neighboring phospho-tyrosine residues (*55*). Since levels of activated ZAP70 are undetectable for conventional T cells undergoing already robust calcium signaling in response to single antigens, we presume that only few catalytically active ZAP70 molecules are required for T cell activation.

We consider it likely that realizing such degree of functionality will be critical to prevent in a tumor setting the selective outgrowth of antigen escape variants, i.e., by promoting sustained T cell effector function after autologous cell transfer while sustaining exquisite T cell sensitivity toward TAAs (*56*). Our findings justify further in vivo preclinical testing of TCC-modified T cells in murine in vivo cancer models. Recent studies have suggested superior in vivo antitumor activity of CD19 εTRuC (*28*)– and CD19 STAR (*29*, *37*)–modified T cells. Yet, without exact knowledge of target cell–expressed CD19 levels, the extent to which CD19 εTRuC- or CD19 STAR-modified T cells achieve eradication of CD19^low^ tumor cells, as they may emerge under selective pressure following CAR T cell therapy, remains to be identified.

Last but not least, an important consideration concerning the clinical deployment of TCCs arises from our serendipitous observation that T cells with high T1 TCC expression efficiently killed A2-expressing K562 cells in the absence of antigenic peptide (fig. S9). This phenomenon, which likely resulted from residual cross reactivity exerted by T1-scF_V_–based receptors toward abundantly expressed endogenous A2/peptide complexes, demonstrates the potential for off-target cytolysis by T cells engineered with signaling-enhanced antigen receptors and underscores the necessity for further structural refinement and rigorous screening approaches to mitigate such risks before any clinical application.

## MATERIALS AND METHODS

### Constructs for lentiviral transduction and CRISPR-Cas9–mediated knock-ins

HLA.A2/NY.ESO-1–specific lentiviral constructs were generated by Gibson assembly and cloned into lentiviral vector p526. Sequences from the T1 scF_V_ and the CD19-Kymriah CAR construct (*6*, *33*, *38*) were cloned into the lentiviral vector epHIV7. T1 BBz CAR was generated by exchanging the FMC63 scF_V_ by the T1 scF_V_ within the CD19 BBz CAR construct. T1 εTRuC and CD19 εTRuC were generated by tethering the T1 scF_V_ and FMC63 scF_V_ to CD3ε, respectively, via the flexible linker (G_4_S)_3_. The CD19 εTRuC T2A green fluorescent protein (GFP) lentiviral construct was a gift from W. Schamel, University of Freiburg. T1 STAR_nat_ was generated by directly tethering T1-V_H_ to the murine TCRα constant region (UniProt: A0A075B662) and T1-V_L_ to the murine TCRβ constant region (UniProt: A0A075B5J4). T1 STAR_mir_ was generated by directly tethering T1-V_L_ to the murine TCRα constant region and T1-V_H_ to the murine TCRβ constant region. All lentiviral constructs contained a copGFP transduction marker separated by a viral T2A cleavage sequence (*57*).

For CRISPR-Cas9–mediated knock-ins of the RA14 or 1G4 (wild type and c58c61) TCR, double-stranded DNA polymerase chain reaction (PCR) products containing the TCRβ chain, T2A, TCRα chain, a stop codon, and a poly-A flanked by the *TRAC*-specific homology arms were produced via PCR as described (*32*). For STAR_nat_, STAR_mir_, and the T1 BBz CAR, double-stranded DNA PCR products containing the synthetic receptor encoding genes, T2A, copGFP, a stop codon, and a poly-A flanked by the *TRAC*-specific homology arms were produced. Please refer to the Supplementary Materials for DNA sequences encoding the TCRs and NY-ESO-1–specific synthetic receptors.

### Generation of lentiviral and CRISPR-Cas9 specificity–redirected T cells

CD8^+^ T cells were isolated from HLA.A2^−^ donors providing informed consent under the approval of the ethics committee of the Medical University of Vienna (ethics commission number: 2001/2008) using magnetic-activated cell sorting (Miltenyi Biotec, catalog no. 130-096-495). Anti-CD3/CD28/CD2 Immunocult (STEMCELL Technologies, catalog no. 10970) was used for T cell activation. T cells were cultivated at 37°C and in 5% atmospheric CO_2_ in Immunocult XF T cell expansion medium (STEMCELL Technologies, catalog no. 10981) and recombinant interleukin-2 (IL-2; 50 U ml^−1^; Novartis, NDC:0078-0495).

For lentiviral transduction, polybrene (Sigma-Aldrich, catalog no. TR-1003-G) was added to the medium containing the lentivirus on the following day at a concentration of 8 μg ml^−1^ to stably integrate synthetic receptor-encoding genes. On day 7 after transduction, the percentage of transduced cells was determined for each condition by assessing copGFP expression and A2/9V-AF647 staining by flow cytometry. Antigen receptor–expressing cells were enriched via fluorescence-activated cell sorting (FACS; see below).

For CRISPR-Cas9–mediated replacement of the TCR, TCRα chain and TCRβ chain were knocked out 2 days after Immunocult-based activation as described (*45*). Briefly, *TRAC* and *TRBC* were targeted using ribonucleoprotein (RNP) based on the following CRISPR RNA (crRNA) (Integrated DNA Technologies): 5′-GGAGAATGACGAGTGGACCC-3′ for *TRBC* (targeting both *TRBC1* and *TRBC2*) and 5′-AGAGTCTCTCAGCTGGTACA-3′ for *TRAC*. crRNA was incubated with trans-activating RNA (Integrated DNA Technologies, catalog no. 1072533), both at 80 μM and at 95°C for 5 min, and then cooled down to 25°C, after which Cas9 nuclease (Integrated DNA Technologies, catalog no. 108 1059) was added. Cas9-RNP mix was stored on ice before use. CRISPR-Cas9–mediated knock-in of the RA14, 1G4 (wild type and c58c61) TCR, STAR_mir_, STAR_nat_, or T1 CAR was performed as described (*32*). A total of 5 × 10^6^ T cells were electroporated (Amaxa Nucleofector II) in 100-μl electroporation buffer (Lonza, catalog no. LONVPA-1002) in the presence of Cas9-RNP and 2 μg of the respective DNA PCR product. After electroporation, cells were cultured in the presence of IL-2 (180 U ml^−1^) overnight and maintained at IL-2 (50 U ml^−1^) for 7 to 10 days as described above.

Genetically modified T cells were enriched via FACS (Sony SH800) using copGFP expression, anti-TCR mAbs (IP26-AF488 or IP26-AF647, BioLegend, catalog nos. B306711 and B306714), anti-CD8 mAbs (OKT8-APC-eFluor780 or OKT8-AF700, Thermo Fisher Scientific, catalog nos. 47-0086-42 and 56-0086-42), and A2/CMV- or A2/9V-containing streptavidin-phycoerythrin (PE) and streptavidin-allophycocyanin (APC) tetramers (BioLegend, catalog nos. B405203 and B405207). In selected cases, we used monomeric A2/9V-AF647 or CD19-AF647 to stain antigen receptors.

For antigen-dependent expansion, HLA.A2/NY-ESO-1–specific cells were cocultured with HLA.A2- and CD80-expressing K562 cells [in-house modified from American Type Culture Collection (ATCC) cell line CCL-243] added at a ratio of 5:1 to 3:1, which had been irradiated with 80 Gy and pulsed with 10 μM NY-ESO-1/9V peptide. RA14 TCR T cells were cocultured with K562 cells irradiated with 80 Gy and pulsed with 10 μM CMV peptide (NLVPMVATV), and CD19-specific cells were cocultured with irradiated TM-LCL (EBV-transformed lymphoblastoid B-cell line) feeder cells (provided by M. Hudecek, Universitätsklinikum Würzburg). Every second day, half of the medium was exchanged with fresh T cell medium containing recombinant IL-2 (50 U ml^−1^). Experiments were conducted at days 8 to 10 of the expansion protocol and 16 or more hours after medium exchange.

For generation of T1 STAR_nat_ and T1 STAR_mir_ TCRαβ^−^ cells (fig. S4), T cells were first subjected to lentiviral transduction and one round of expansion as described above. Next, T cells were restimulated with Immunocult in preparation for CRISPR-Cas9–mediated TCR knockout as described above.

### Quantitation of cell surface synthetic receptor molecules

Engineered T cells were stained with increasing concentrations of AF647-conjugated antihuman TCR α/β antibody, A2/9V-AF647, or CD19-AF647 to determine the concentration of the staining agent leading to label saturation for CMV-specific RA14 TCR T cells, A2/NY-ESO-1–specific engineered T cells, and CD19-specific CAR T cells. The mean fluorescence intensity (MFI) of each cell population was measured by flow cytometry. The MFI of AF647-conjugated quantitation beads (Quantum MESF; Bangs laboratories, catalog no. 647) was determined to give rise to a calibration curve allowing to standardize fluorescence intensity (MESF). MFI values of cell populations were converted to MESF units according to the manufacturer’s instructions.

### Protein expression and purification

Codon-optimized cDNAs encoding the extracellular domains excluding the leader sequence of HLA.A2 (UniProt: P01892) and beta-2-microglobulin (UniProt: P61769) were cloned into pET-28b and pHN1, respectively. A DNA sequence coding for a polyhistidine tag, which harbored 12 consecutive histidine residues (H_12_), was introduced C-terminally to HLA.A2 within pET28b. HLA.A2-H_12_ and beta-2-microglobulin were expressed in *Escherichia coli* BL21 (DE3, Agilent, catalog no. 200131) as inclusion bodies and refolded in the presence of peptide to give rise to HLA.A2-peptide complexes (*58*, *59*). After completion of the refolding reaction, 300 ml of protein solution was dialyzed three times against 10 liters of 1× phosphate-buffered saline (PBS). Correctly conformed HLA.A2-peptide complexes were purified using Ni^2+^-NTA agarose chromatography (HisTrap excel, 2 × 5 ml columns connected in series; Cytiva, catalog no. 11004528) followed by size exclusion chromatography (Superdex 200 Increase 10/300 GL; Cytiva, catalog no. 28990944). cDNAs encoding the extracellular portions of the human protein ICAM-1 (UniProt: P05362) without the leader sequence were PCR-amplified and cloned into the pAcGP67 of the BaculoGold Baculovirus Expression System (BD Biosciences). This vector was modified so that all proteins featured a C-terminal H_12_-tag. We produced virus using the Baculovirus Expression system according to the manufacturer’s instructions. High-5 cells (BTI-Tn-5B1-4; Thermo Fisher Scientific, catalog no. B85502) were infected with virus particles. The supernatant was collected 4 days after infection at 90% cell viability. After centrifugation and filtration (0.45 μm), it was dialyzed against PBS by tangential flow filtration (Minimate Tangential Flow Filtration System equipped with a 10-kDa T-Series cassettes; Pall Corporation). The supernatant containing the proteins was concentrated eightfold and then diluted eightfold with PBS for a total of three times for buffer exchange. The processed supernatant was concentrated once more eightfold and then subjected to Ni^2+^-NTA agarose chromatography. Protein was eluted with PBS/300 mM imidazole (pH 7.4) and subjected to size exclusion chromatography (Superdex 200 Increase 10/300 GL and Superdex 75 Increase 10/300 GL; Cytiva, catalog no. 29148721) and anion-exchange chromatography (Mono Q 5/50 GL; Cytiva, catalog no. 17-5166-01). The extracellular portion of CD19 (UniProt: P15391; https://www.uniprot.org/uniprot/P15391) was C-terminally fused to a polyhistidine tag containing 10 histidine residues, expressed in human embryonic kidney cells (ATCC no. CRL-1573) and purified via Ni^2+^-NTA agarose affinity chromatography followed by size exclusion chromatography and anion-exchange chromatography (*60*).

All purification steps were performed on an ÄKTA pure chromatography system (Cytiva). For all proteins, the fractions containing properly conformed proteins were identified using SDS–polyacrylamide gel electrophoresis followed by silver staining. Immediately after purification, protein was labeled and/or adjusted to 50% glycerol in 1× PBS or snap-frozen in liquid N_2_ for storage at −80°C.

### Fluorophore conjugation

Two hundred micrograms of HLA.A2-peptide complex in PBS was concentrated to 1 mg ml^−1^ with the use of Amicon Ultra Centrifugal Filters (Cytiva, catalog no. UFC901024), adjusted to pH 8.3 by adding freshly prepared NaHCO_3_ (0.1 M final concentration; Carl Roth GmbH, catalog no. 6885.1) and incubated for 30 min at room temperature, with *N*-hydroxysuccinimidyl ester derivatives of AF647 (Thermo Fisher Scientific, catalog no. A20006) according to the manufacturer’s instructions.

### SLB preparation

Supported lipid bilayers (SLBs) were prepared as described (*61*). Briefly, DGS–Ni-NTA and POPC (both from Avanti Polar Lipids, catalog nos. 790404P-25 mg and 850457C-25 mg) were dissolved in chloroform (Carl Roth GmbH, catalog no. 3313.1) and mixed at a 1:50 molar ratio. Lipids were dried under vacuum overnight in a desiccator, then resuspended in 10 ml of degassed PBS, and sonicated in a water bath sonicator (Q700; Qsonica) under nitrogen at 120 to 170 W for at least 60 min until the suspension had lost turbidity. To remove non-unilamellar vesicles, the lipid suspension was centrifuged for 1 hour at 37,000 rpm (150,000*g*) at room temperature using a Sorvall RC M150GX ultracentrifuge with a S150AT-0121 rotor (Thermo Fisher Scientific). The supernatant was obtained and centrifuged again for 8 hours at 43,000 rpm (288,000*g*) at 4°C using the same rotor and centrifuge. The supernatant was filtered (0.2 μm) (Filtropur S 0.2; Sarstedt) and stored at 4°C for up to 6 months.

Glass slides (22 mm by 64 mm, no. 1.5 borosilicate; Menzel-Gläser) were plasma-cleaned (Zepto, Diener Electronic) for 15 min and attached with the use of dental imprint silicon putty (Picodent twinsil 22, Picodent) to a Lab-Tek 12-well chamber (Thermo Fisher Scientific) from which the original glass bottom had been removed. Slide-exchanged chambers were then incubated with lipid vesicle suspension for at least 10 min, after which they were extensively rinsed with PBS. For functionalization, SLBs were incubated for 60 min with protein-containing PBS and then rinsed twice with 15 ml of PBS to remove unbound protein.

### Surface plasmon resonance for T1 scF_V_ affinity and kinetics

T1 scF_V_s were produced by Absolute Antibody Ltd. Surface plasmon resonance experiments were performed with a Biacore T200 instrument (Cytiva). All experiments were conducted in degassed and filtered HBS-EP (10 mM Hepes, 150 mM NaCl, 3 mM EDTA, 0.005% Tween-20), at 37°C. Chip activation was performed applying 200 μl of sample volume at a flow rate of 10 μl min^−1^ with 600-s contact time for 1-ethyl-3-(3-dimethylaminopropyl)-carbodiimide and *N*-hydroxysuccinimide followed by application of 10 μl of streptavidin in 190 μl of acetate (pH 4.5) and 300-s contact time for ProteON before application of glycine buffer (pH 2.5).

Refolded and biotinylated pMHCs were immobilized on a CM5 S series sensor chip at a flow rate of 10 μl min^−1^. Surface densities were set to roughly 300 or 1000 resonance units (RUs) for kinetic and affinity measurements, respectively. Recombinant CD58 (biotinylated) was used as a negative control in the reference cell at a similar surface density, due to its comparable size. Flow cells were blocked by injecting d-Biotin twice at a concentration of 250 μM for 60 s at a flow rate of 10 μl min^−1^. The system was equilibrated by doing 10 start-up cycles that resembled the parameters for flow rate and contact time for the consecutive sample measurements. Recombinant scF_V_s were injected into the system using the “low consumption rate” at a flow rate of either 1 or 10 μl s^−1^ for either 20 or 180 s. Ten different dilutions were injected consecutively into the system, followed by a dissociation step that lasted for 300 s.

For the determination of binding affinities, RUs were measured at the end of the association phase of a given concentration of the recombinant protein. RUs were plotted against the concentration of the recombinant protein, and curve fitting was done with GraphPad Prism 7.04 with the “One Site – specific binding” function. For the determining of dissociation rates, a “One Phase Decay” function was fit to the data.

### Microscopy setup

Microscopy was conducted using two inverted setups. One setup allowed for TIR-based imaging and was built around an Eclipse Ti-E microscope body (Nikon Instruments) that was equipped with a chromatically corrected 100× TIR objective (CFI SR Apo TIR 100× oil numerical aperture: 1.49; Nikon Instruments), a 647-nm diode laser (OBIS, Coherent) for excitation, and a custom-made Notch filter (Chroma Technology) to block reflected stray light of 647 nm from reaching the camera. The setup was furthermore equipped with an ET700/75 emission bandpass filter (Chroma Technology) present in the emission pathway and a dichroic (QUAD Cube). An iXon Ultra 897 EMCCD camera (Oxford Instruments) was used for data recording. An eight-channel DAQ-board PCI-DDA08/16 (National Instruments) in combination with the microscopy automation and image analysis software MetaMorph (v. 7.8.13.0) (Molecular Devices) were used to program and apply timing protocols and control all hardware components of the microscope components.

A second inverted microscope (DMI4000; Leica Microsystems) was equipped with a 20× objective (HC PL FLUOTAR 20×/0.50 PH2∞/0.17/D, Leica Microsystems) and with a mercury lamp (EL6000; Leica Microsystems) for Fura-2–based calcium recordings. This microscope was equipped with a fast filter wheel containing 340/26 and 387/11 excitation bandpass filters (both Leica Microsystems). Data were recorded using a sCMOS (scientific Complementary Metal–Oxide–Semiconductor) Andor Prime95b (Photometrix). Open-source software μManager was used to program and control all hardware components.

### Measurements of antigen densities on SLBs

SLB antigen density was determined by counting the number of diffraction-limited fluorescent events within a region of interest (ROI) or by dividing the fluorescence intensity value within a ROI by the single-molecule fluorescent intensity value. For bilayers featuring clearly distinguishable diffraction-limited fluorescence events, 30 images were recorded within a ROI of 100 × 100 pixels. The total number of molecules within the ROI of each image was determined using the Fiji Thunderstorm plugin (ImageJ/Fiji) and corrected for pixel size and number of images to determine the antigen density (1 pixel ≙ 0.0256 μm^2^, 100 × 100 pixels = 10,000 pixels ≙ 256 μm^2^). For determining antigen densities of SLBs, the average single-molecule fluorescence intensity value of at least 300 molecules was determined within the ROI using the Fiji Thunderstorm plugin as described above. The average integrated intensity value of ROIs of 10 images was determined and divided by the average single-molecule intensity value to arrive at the number of molecules within a given ROI. Last, this value was corrected for pixel size to determine antigen density. To quantitate low antigen densities giving rise to distinct diffraction-limited fluorescence events, we counted the number of events within a given ROI for at least 30 images and divided the average number by the area of the ROI. Fluorophore densities were lastly converted to antigen densities by factoring in the protein-to-dye ratio determined by spectrophotometry.

### Determination of mobile fraction using FRAP

For determination of the SLB-resident mobile protein fraction, we used fluorescence recovery after photo bleach (FRAP) as described (*61*). A circular aperture was placed in the beam path. We recorded 3 images before bleaching the defined area of the bilayer and another 10 images after the bleach pulse at 1-min time intervals. Fluorescence intensity values recorded after bleaching were normalized against the average of the fluorescence intensity values recorded before photobleaching.

### Calcium imaging

Intracellular changes in Ca^2+^ levels were measured using the ratiometric calcium-sensitive dye Fura-2 AM as described (*62*). A total of 9 × 10^5^ cells were incubated in 0.5-ml imaging buffer [Hanks’ balanced salt solution (Thermo Fisher Scientific, catalog no. 14175129), supplemented with 2 mM CaCl_2_ (Carl Roth GmbH, catalog no. T885.2), 2 mM MgCl_2_ (Sigma-Aldrich, catalog no. 1.05833.0250), and 2% fetal calf serum (Biowest)] supplemented with 5 μM Fura-2 AM (Thermo Fisher Scientific, catalog no. 11524766) for 15 min at 37°C, washed twice with 10 ml of imaging buffer, and resuspended in 135-μl imaging buffer. Cells were kept at room temperature for a maximum of 30 min before starting the experiment. Immediately before imaging, PBS was exchanged for imaging buffer. T cells were pipetted into the imaging buffer and allowed to settle for 30 s after which 510/80-nm emission was recorded while rapidly switching between 340- and 387-nm excitation for 20 min at time intervals of 15 s.

An in-house custom-built MATLAB software was used to track cells in each frame using a published particle tracking (*33*). Tracking parameters were chosen so only single cells in contact with the SLB were included. We used the MATLAB software to create ratio images for each frame. “Methods for automated and accurate analysis of cell signals” was used for population analysis as described (*63*). For each trajectory within a population, the ratio was normalized frame-wise to that of the population median of T cells in contact with antigen-free SLBs. T cell traces were categorized into “activated” and “nonactivated” based on a threshold defined by a receiver operating curve (ROC) between a negative control (ICAM-1, no antigen) and a positive control (ICAM-1, + antigen). T cells were categorized as activated when the Fura-2 intensity value remained 80% of the entire trace above the ROC threshold.

For most TCRs and TCCs, we used the ROC threshold of 1.25. For CAR T cells, we had to lower the ROC threshold to 1.15, because, after normalization, the high background signaling in the negative control yielded in the detection of false negative results in the normalized samples.

The proportion of activated T cells was plotted against initial antigen densities and fitted to a three-parameter dose-response curve (hillslope *n* = 1.0) with Eq. 1 to extract the activation threshold (EC_50_), i.e., the antigen density at half-maximum response

$$Y=\text{Bottom}+\frac{(X^{n}\times \left(\text{Top}-\text{Bottom}\right)}{\left(X^{n}+\text{EC}50^{n}\right)}$$ (1)

The calcium histograms were compiled from the measured population values of the median Fura-2 AM ratio corresponding to the first 10 frames after the peak Fura-2 AM ratio value within the trajectory. The latter was normalized frame-wise to the population median of the negative control, i.e., cells confronted with antigen-free SLBs.

The quality of the calcium response of A2/NY-ESO-1–specific T cells was derived from the mean of the Fura-2 ratio value histograms (median of peak + 10 frames). Only Fura-2 values larger than the activation threshold (1.15 for CAR T cells, 1.25 for all other engineered T cells) were included in the analysis. The mean of the Fura-2 value distribution was approximated by the sum of all data points divided by the number of data points in the histogram. More specifically, this involved (i) multiplying the height of each bar (i.e., probability density value) by the number the bar is representing (i.e., Fura-2 value), (ii) summarizing each of these products, and (iii) dividing the sum of the products by the sum of the heights of the bar (i.e., sum of all probability density values).

### Quantitation of cell surface synthetic receptor molecule density

Engineered T cells were stained with increasing concentrations of AF647-conjugated antihuman αβTCR antibody and A2/9V-AF647 for determination of label saturation concentration of CMV-specific RA14 TCR T cells and A2/NY-ESO-1–specific engineered T cells, respectively. Labeled cells were dropped in imaging buffer on SLBs functionalized with ICAM-1 and allowed to settle for 8 min. Hereafter, images were recorded for at least 20 cells per condition. The average integrated intensity values of cells were determined, divided by the single-molecule intensity, and corrected for pixel size to determine the antigen density in molecules per square micrometer.

### Quantitation of synapse-associated CD3ζ pITAM#2 and pZAP70

Prewarmed T cells were seeded in imaging buffer on SLBs and allowed to settle for 12 min at 37°C. T cells were fixed for 30 min at room temperature in PBS supplemented with 20 mM sodium fluoride (Sigma-Aldrich, catalog no. S1504), 4% formaldehyde (Thermo Fisher Scientific, catalog no. 28908), 0.2% Triton X-100 Surfact-Amps (Thermo Fisher Scientific, catalog no. 28314), and 2 mM Na_3_O_4_V (Sigma-Aldrich, catalog no. S6508-50G). Formaldehyde was then gently washed away with washing buffer consisting of PBS supplemented with 20 mM NaF, 2 mM Na_3_O_4_V, 0.2% Triton X-100, and 3% bovine serum albumin (Applichem GmbH, catalog no. A1391,0500). After three washes, SLBs were incubated for 20 min at room temperature to block nonspecific binding. T cells were incubated overnight at 4°C with either mAb reactive to phospho-CD247 (CD3 zeta) (Tyr^111^ and Tyr^123^) (clone EM-55; Thermo Fisher Scientific, catalog no. MA5-28539) or mAb reactive to ZAP70 phosphorylated at position 319 (clone 1503310; BioLegend, catalog no. 683702). The next day, unbound antibody was washed away with washing buffer, incubated with goat anti-mouse immunoglobulin G (H+L)–AF647 or immunoglobulin G (H+L)–AF555 (Thermo Fisher Scientific, catalog no. A32728 or A-21422) and incubated for 3 hours at room temperature. Unbound antibody was washed away using washing buffer. Cells were stored at 4°C in the dark until acquisition of images. Integrated intensity values were recorded as described earlier with the exception that cells had been fixed.

### Quantitation of IFN-γ secretion after antigenic stimulation of T cells on SLBs

IFN-γ production was quantitated through enzyme-linked immunosorbent assay (ELISA) of supernatants of 50,000 T cells that had been stimulated through antigen-functionalized SLBs harboring different ligand densities. Before stimulation, SLB antigen density was determined after which buffer was exchanged to Immunocult XF T cell expansion medium. Hereafter, T cells were seeded and incubated for 72 hours. The amount of secreted IFN-γ was determined using the ELISA MAX Deluxe Set Human IFN-γ kit (BioLegend, catalog no. B430104) according to the manufacturer’s instructions.

### Killing assay of peptide-pulsed K562

HLA.A2^+^, luciferase firefly-BFP^+^ K562 cells were pulsed with different concentrations of 9V peptide at 37°C for 1 hour. Subsequently, 30,000 T cells were added in Immunocult XF T cell expansion medium and d-luciferin firefly (75 μg ml^−1^; Biosynth, catalog no. L-8220). Cytotoxicity by engineered T cells was determined after coculturing peptide-pulsed K562 cells at an effector to target ratio of 1:1 (30,000 cells each). Cells were cocultured at 37°C under 5% atmospheric CO_2_. K562 treated with 50% dimethyl sulfoxide were used as maximum killing control. K562s cocultured with 1G4 T cells in the absence of antigen were used as spontaneous death control. Relative light units (RLU) indicating K562 viability were measured after 6, 8, and 24 hours on the microplate reader Mithras LB 940 (Berthold) or the Spark Multimode Microplate Reader (Tecan). Specific cell lysis was determined with Eq. 2

$$\begin{array}{c} \%\text{Specific lysis}=100\times \\ \frac{\left(\text{Spontaneous death}\;\text{RLU}-\text{test}\;\text{RLU}\right)}{\left(\text{Spontaneous death}\;\text{RLU}-\text{maximum killing}\;\text{RLU}\right)} \end{array}$$ (2)
