# Supplementary material for: TCR/CD3-based synthetic antigen receptors (TCC) convey superior antigen sensitivity combined with high fidelity of activation
Source: Sci Adv. 2024 Sep 4;10(36):eadj4632. doi: 10.1126/sciadv.adj4632 (PMC11373591; doi:10.1126/sciadv.adj4632)
Supplement: Supplementary file 1 — Figs. S1 to S9 Table S1 Data S1 to S11 [file sciadv.adj4632_sm.pdf]

Science Advances
AAAS

Supplementary Materials for

TCR/CD3-based synthetic antigen receptors (TCC) convey superior antigen sensitivity combined with high fidelity of activation

Vanessa Mühlgrabner *et al.*

Corresponding author: René Platzer, rene.platzer@meduniwien.ac.at;
Johannes B. Huppa, johannes.huppa@meduniwien.ac.at

Sci. Adv. **10**, eadj4632 (2024)
DOI: 10.1126/sciadv.adj4632

This PDF file includes:

Figs. S1 to S9
Table S1
Data S1 to S11

Figure S1

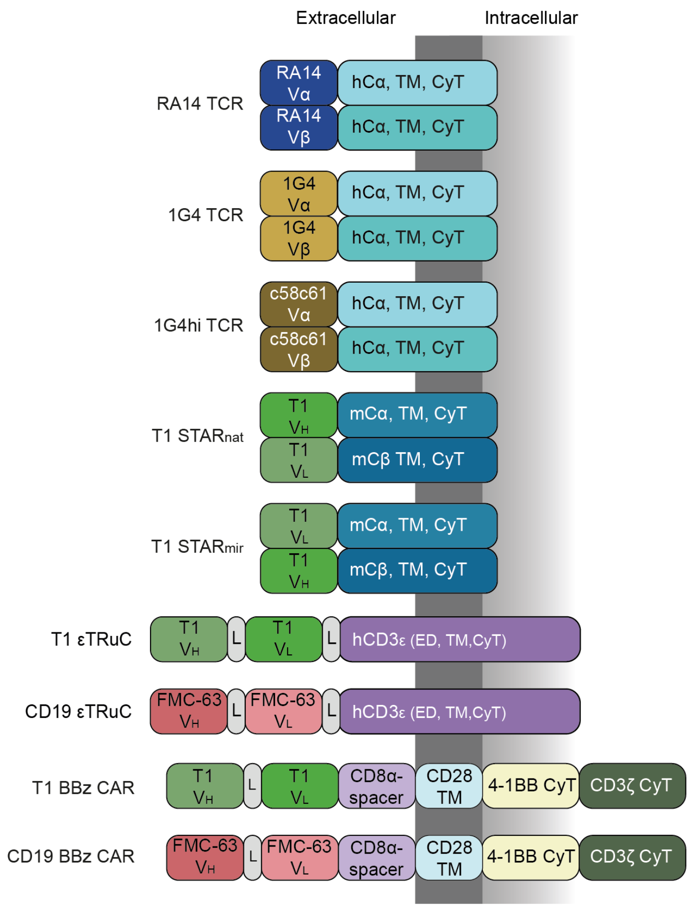

Extracellular
Intracellular
RA14 TCR
RA14 Vα
hCα, TM, CyT
RA14 Vβ
hCα, TM, CyT
1G4 TCR
1G4 Vα
hCα, TM, CyT
1G4 Vβ
hCα, TM, CyT
1G4hi TCR
c58c61 Vα
hCα, TM, CyT
c58c61 Vβ
hCα, TM, CyT
T1 STARnat
T1 VH
mCα, TM, CyT
T1 VL
mCβ TM, CyT
T1 STARmir
T1 VL
mCα, TM, CyT
T1 VH
mCβ, TM, CyT
T1 εTRuC
T1 VH
L
T1 VL
L
hCD3ε (ED, TM,CyT)
CD19 εTRuC
FMC-63 VH
L
FMC-63 VL
L
hCD3ε (ED, TM,CyT)
T1 BBz CAR
T1 VH
L
T1 VL
CD8α-spacer
CD28 TM
4-1BB CyT
CD3ζ CyT
CD19 BBz CAR
FMC-63 VH
L
FMC-63 VL
CD8α-spacer
CD28 TM
4-1BB CyT
CD3ζ CyT

Figure S1: Schematic illustration of TCRs, TCCs and CARs employed in this study. V_H = variable domain of the heavy chain, V_L= variable domain of the light chain, L = linker, Vα = variable domain of TCRα, Vβ = variable domain of TCRβ, hCα(β) = constant domain of human TCRα(β), mCα(β) = constant domain of murine TCRα(β), TM = transmembrane domain, CyT = cytoplasmic tail

Figure S2

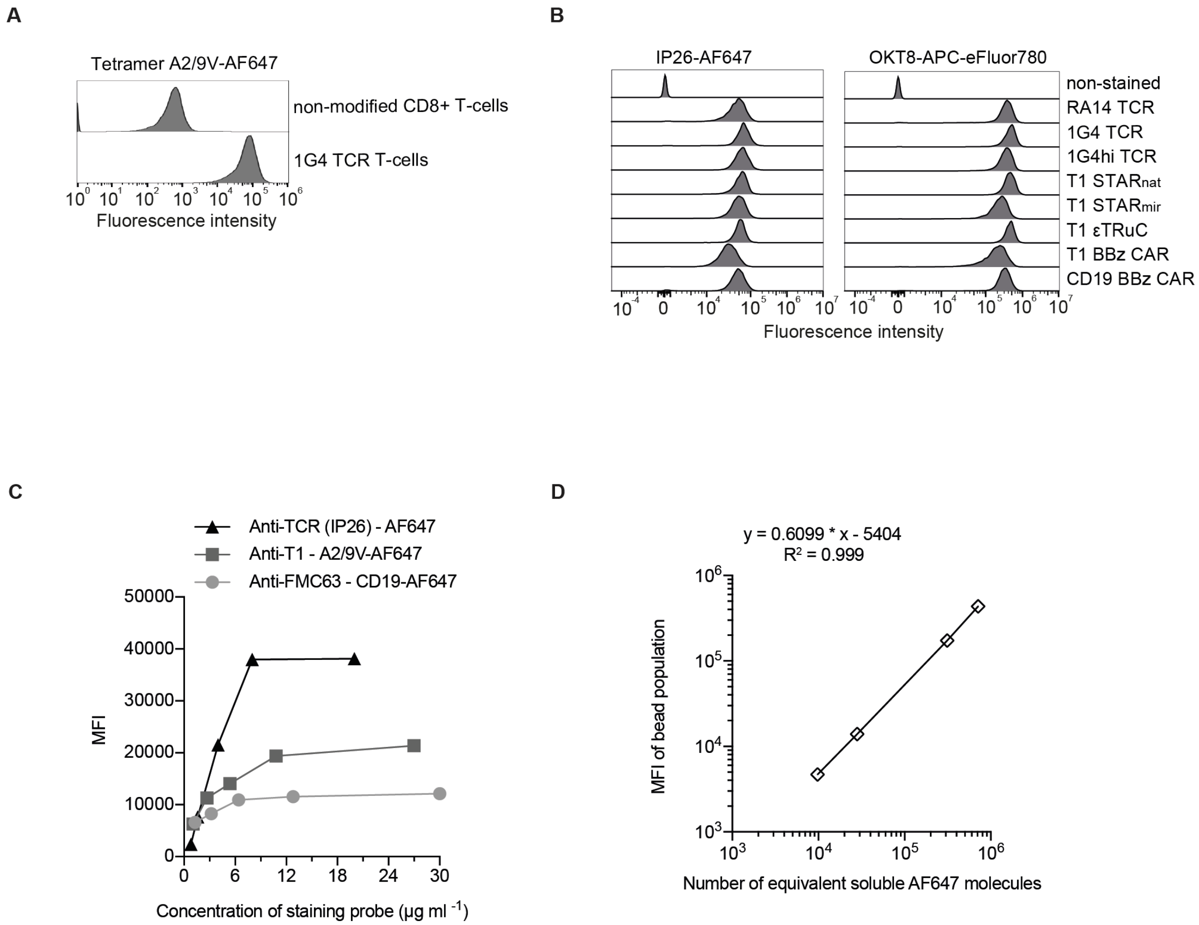
A
Tetramer A2/9V-AF647
non-modified CD8+ T-cells
1G4 TCR T-cells
Fluorescence intensity
B
IP26-AF647
OKT8-APC-eFluor780
non-stained
RA14 TCR
1G4 TCR
1G4hi TCR
T1 STARnat
T1 STARmir
T1 εTRuC
T1 BBz CAR
CD19 BBz CAR
Fluorescence intensity
C
Anti-TCR (IP26) - AF647
Anti-T1 - A2/9V-AF647
Anti-FMC63 - CD19-AF647
MFI
Concentration of staining probe (µg ml -1)
D
y = 0.6099 * x - 5404
R² = 0.999
MFI of bead population
Number of equivalent soluble AF647 molecules

Figure S2: Flow cytometric analysis of antigen receptor surface expression. (**A**) Surface staining of 1G4 TCRs via A2/9V-AF647 tetramers. (**B**) Engineered T-cells were labeled with TCR-reactive IP26-AF647 (left panel) and CD8-specific OKT8-APC-eFluor780 (right panel). (**C**) RA14 TCR T-cells, T1 BBz CAR T-cells and CD19 BBz CAR T-cells were stained as indicated with A2/CMV-AF647, A2/9V-AF647 and CD19-AF647 to determine probe concentrations required for label saturation. (**D**) Calibration curve of AF647 intensity values determined with the use of Quantum™ AF647 MESF beads.

Figure S3

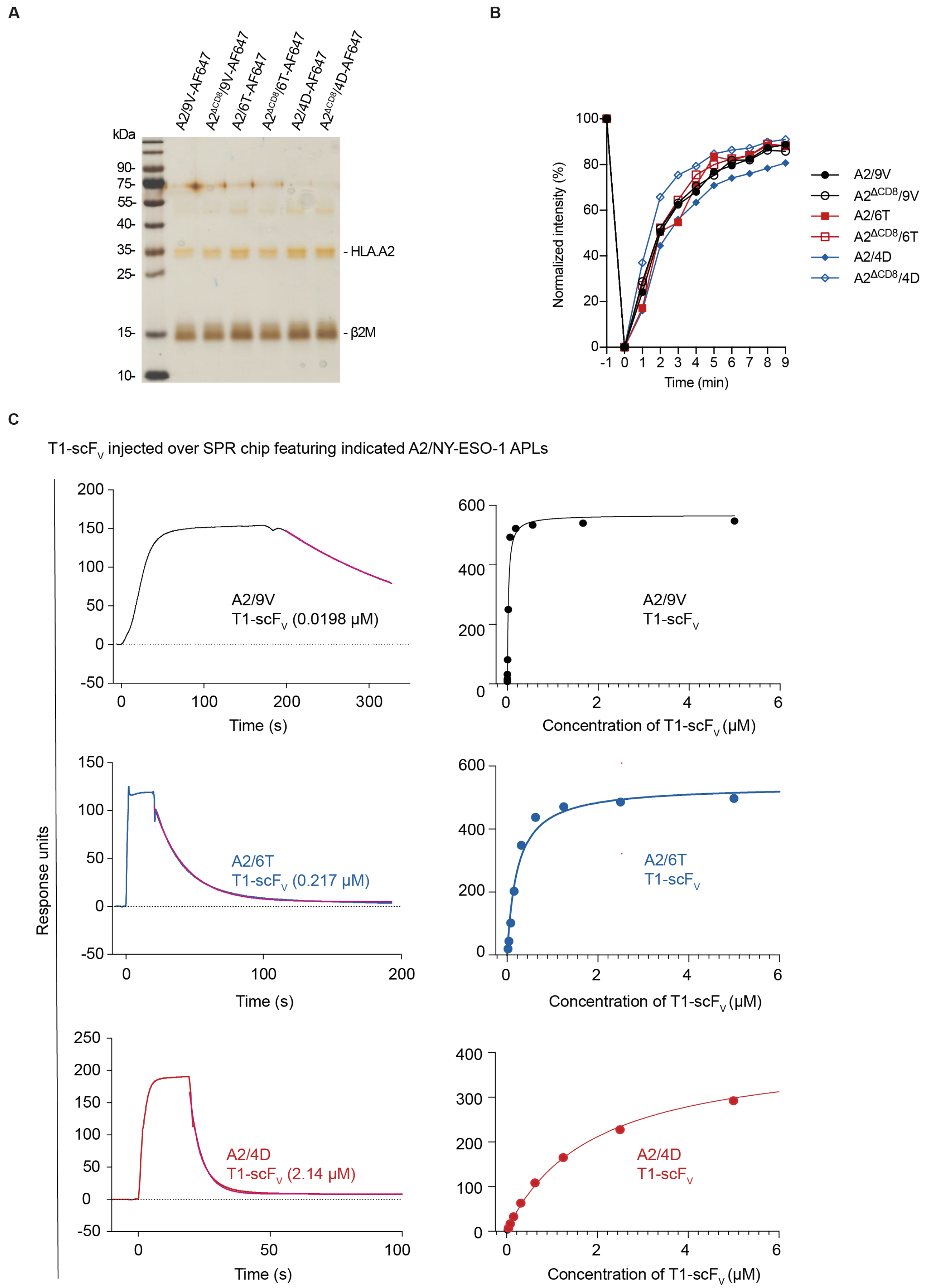
A
B
A2/9V-AF647
A2ΔCD8/9V-AF647
A2/6T-AF647
A2ΔCD8/6T-AF647
A2/4D-AF647
A2ΔCD8/4D-AF647
kDa
90-
75-
55-
40-
35-
25-
15-
10-
- HLA.A2
- β2M
Normalized intensity (%)
Time (min)
A2/9V
A2ΔCD8/9V
A2/6T
A2ΔCD8/6T
A2/4D
A2ΔCD8/4D
C
T1-scFV injected over SPR chip featuring indicated A2/NY-ESO-1 APLs
Response units
A2/9V
T1-scFV (0.0198 μM)
Time (s)
A2/9V
T1-scFV
Concentration of T1-scFV (μM)
A2/6T
T1-scFV (0.217 μM)
Time (s)
A2/6T
T1-scFV
Concentration of T1-scFV (μM)
A2/4D
T1-scFV (2.14 μM)
Time (s)
A2/4D
T1-scFV
Concentration of T1-scFV (μM)

Figure S3: SPR-based analysis and production of A2/NY-ESO-1 variants for SLB functionalization. (**A**) Analysis of apparent molecular weight via 12.5 % reducing SDS PAGE of all HLA-A2/NY-ESO-1 variants employed for SLB functionalization. (**B**) Fluorescence Recovery After Photobleaching (FRAP) was employed to determine the immobile fraction of SLB-anchored A2/NY-ESO-1 variants. Fluorescence intensities were normalized to the initial intensity values and plotted versus time. Data are representative of n=3 biological replicates. (**C**) SPR-based analysis of the interaction kinetics between the T1-scFV (solute) and HLA.A2 complexed with the altered peptide ligands (APLs) 9V-, 6T- and 4D-NY-ESO-1 as indicated (on the chip). Data are representative for one SPR measurement. Results are summarized in **table S1.**

Figure S4

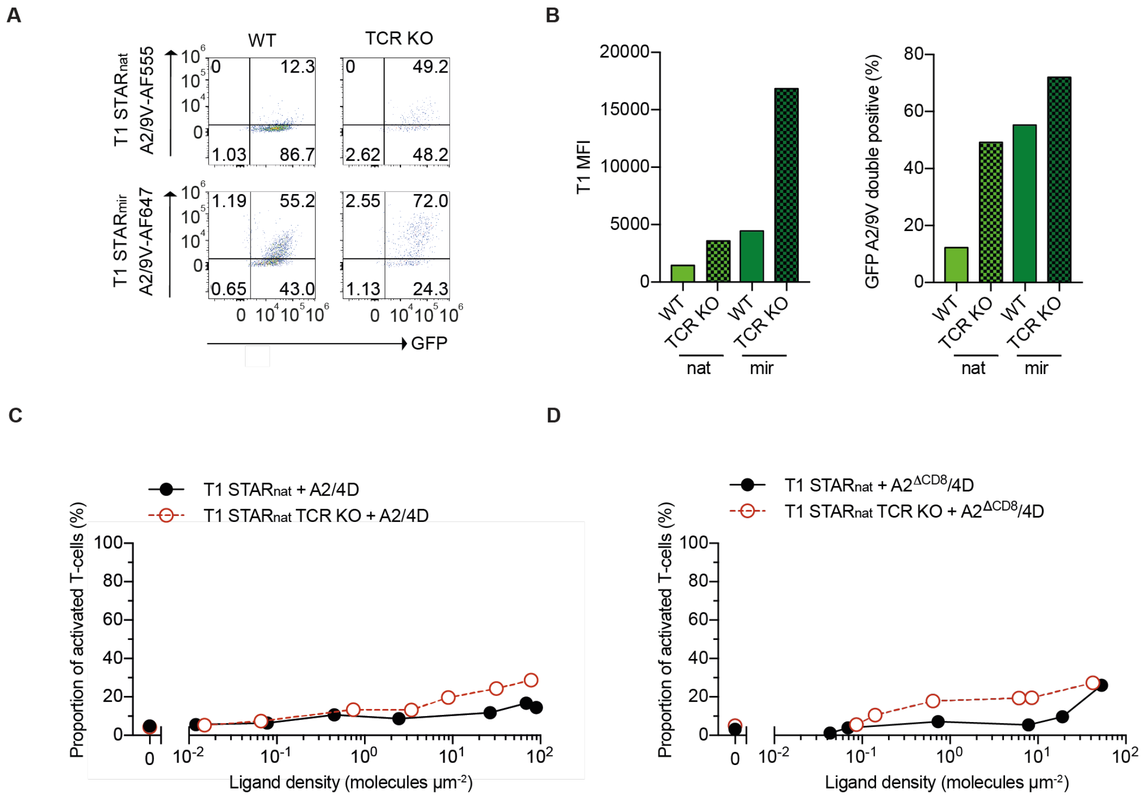
A
WT
TCR KO
T1 STARnat
A2/9V-AF555
T1 STARmir
A2/9V-AF647
12.3
86.7
1.03
49.2
48.2
2.62
1.19
55.2
0.65
43.0
2.55
72.0
1.13
24.3
GFP
B
T1 MFI
GFP A2/9V double positive (%)
WT
TCR KO
nat
mir
C
T1 STARnat + A2/4D
T1 STARnat TCR KO + A2/4D
Proportion of activated T-cells (%)
Ligand density (molecules μm-2)
D
T1 STARnat + A2ΔCD8/4D
T1 STARnat TCR KO + A2ΔCD8/4D
Proportion of activated T-cells (%)
Ligand density (molecules μm-2)

Figure S4: Surface expression of lentivirally introduced T1 $STAR_{nat}$ and T1 $STAR_{mir}$ improves substantially after CRISPR/Cas9-mediated genetic ablation of endogenous TCRαβ, but does not majorly affect the sensitivity of T1 $STAR_{nat}$ T-cells towards the A2/4D low affinity ligand. (**A**, **B**) Flow cytometric analysis of surface expression in TCR^{+} (WT) and TCR^{-} (KO) T-cells expressing T1 $STAR_{nat}$ and T1 $STAR_{mir}$ with the use of A2/9V-AF555 and A2/9V-AF647, respectively. (**C**, **D**) T1 $STAR_{nat}$ T-cell calcium response towards SLBs featuring ICAM-1 and A2/4D or $A2^{\Delta CD8}$/4D at indicated densities. Data are representative of one donor.

Figure S5

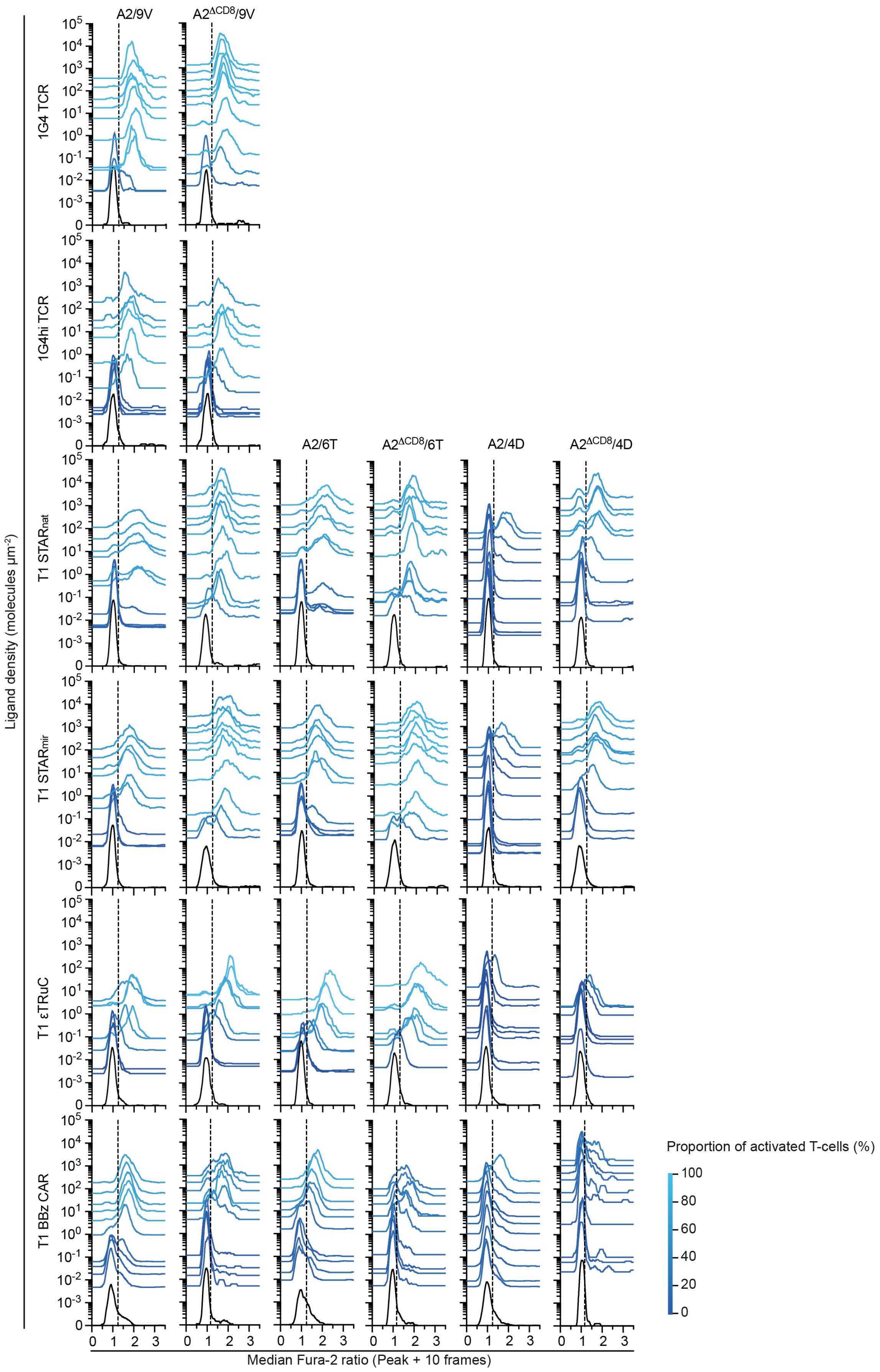

A2/9V
A2$^{\Delta CD8}$/9V
A2/6T
A2$^{\Delta CD8}$/6T
A2/4D
A2$^{\Delta CD8}$/4D
1G4 TCR
1G4hi TCR
T1 STAR$_{nat}$
T1 STAR$_{mir}$
T1 εTRuC
T1 BBz CAR
Ligand density (molecules µm^{-2})
Median Fura-2 ratio (Peak + 10 frames)
Proportion of activated T-cells (%)

Figure S5: Assessment of antigen sensitivity conveyed by NY-ESO-1-specific antigen receptor constructs. Population-based analysis of the calcium response of A2/NY-ESO-1-specific cells (one experiment shown) confronted with indicated A2/NY-ESO-1 variants at indicated densities (data refers to **Fig. 4** and **Fig. 5**). A2/NY-ESO-1-specific constructs were introduced by means of lentiviral transduction (T1 εTRuC) or CRISPR/Cas9-mediated knock in (1G4 TCR, 1G4hi TCR, T1 $STAR_{nat}$, T1 $STAR_{mir}$, T1 BBz CAR). Dashed lines indicate Fura-2 ratio thresholds above which cells were considered activated (i.e., 1.25 for 1G4 TCR T-cells, 1G4hi TCR T-cells, T1 $STAR_{nat}$ T-cells, T1 $STAR_{mir}$ T-cells, T1 εTRuC T-cells and 1.15 for T1 BBz CAR T-cells).

Figure S6

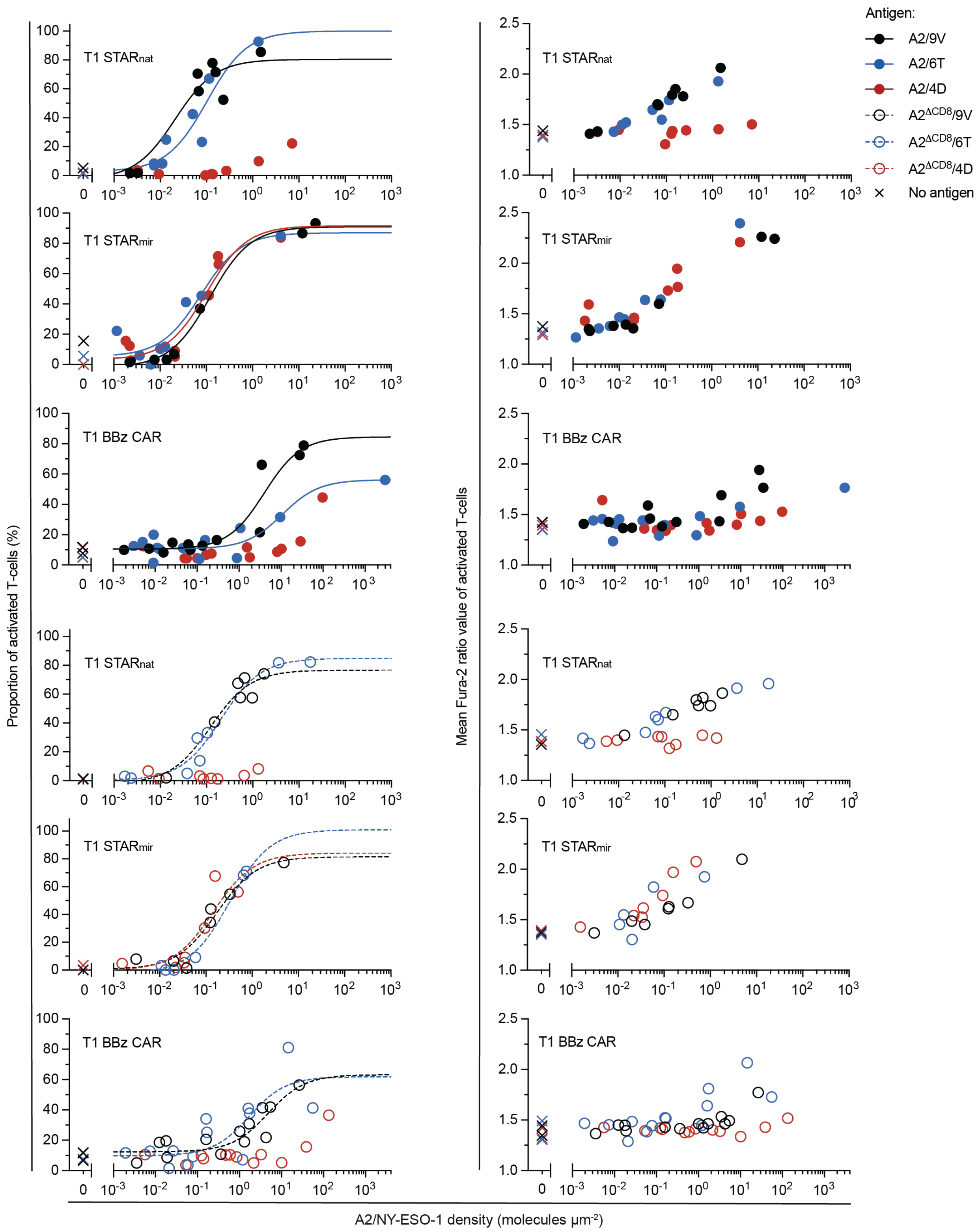

Antigen:
A2/9V
A2/6T
A2/4D
A2ΔCD8/9V
A2ΔCD8/6T
A2ΔCD8/4D
No antigen
T1 STARnat
T1 STARmir
T1 BBz CAR
T1 STARnat
T1 STARmir
T1 BBz CAR
Proportion of activated T-cells (%)
Mean Fura-2 ratio value of activated T-cells
A2/NY-ESO-1 density (molecules μm-2)

Figure S6: Assessment of calcium signaling of T-cells equipped with NY-ESO-1-specific TCCs and CARs. (**A**) Calcium dose-response of lentivirally transduced T1 $STAR_{nat}$ T-cells, T1 $STAR_{mir}$ T-cells and T1 BBz CAR T-cells which had been confronted with SLBs functionalized with A2/9V, A2/6T, and A2/4D at indicated densities. Data are representative of n=1-2 experiments with one to two donors. Data were fitted to a three-parameter dose response curve (equation (1)) to extract EC50 values and 95% confidence intervals as summarized in **Table 1.**

Figure S7

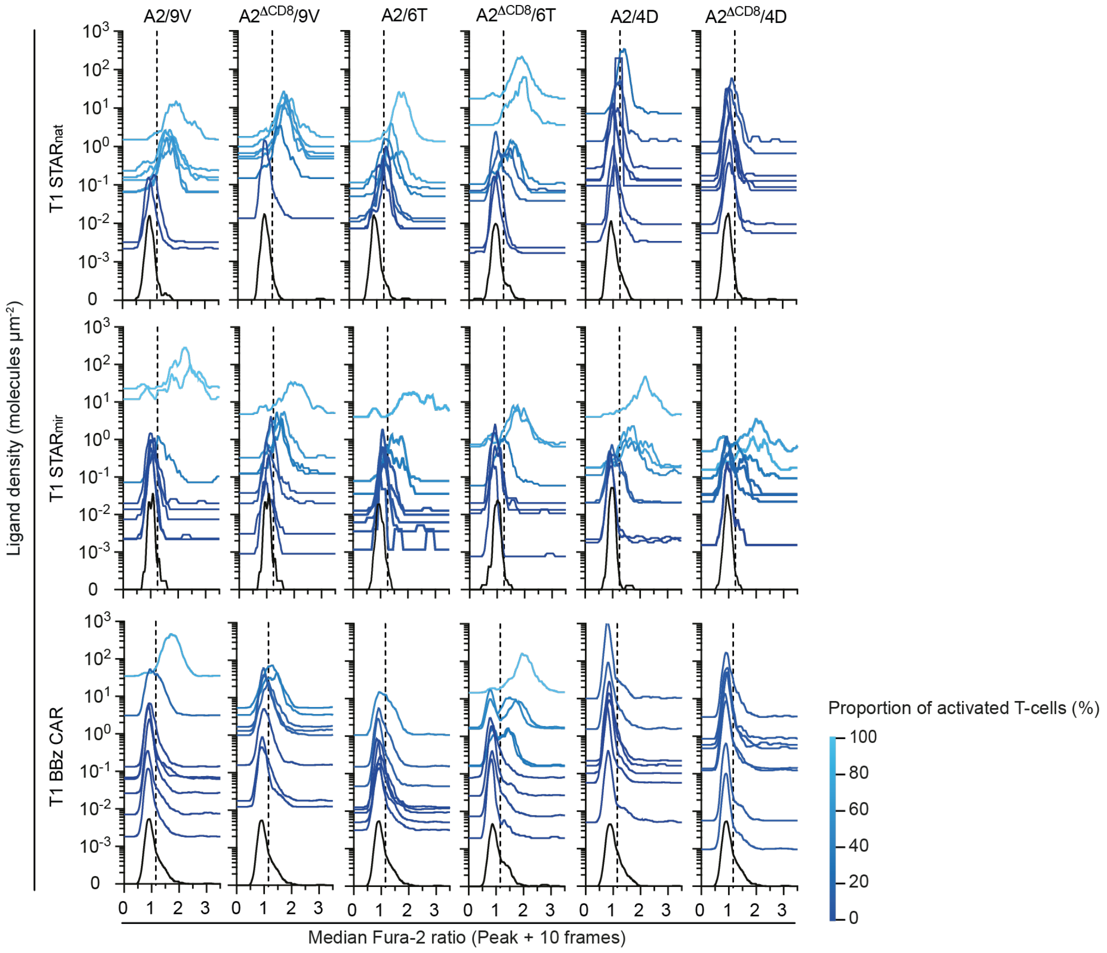
A2/9V
A2^ΔCD8/9V
A2/6T
A2^ΔCD8/6T
A2/4D
A2^ΔCD8/4D
T1 STARnat
T1 STARmir
T1 BBz CAR
Ligand density (molecules µm-2)
Median Fura-2 ratio (Peak + 10 frames)
Proportion of activated T-cells (%)
100
80
60
40
20
0

Figure S7: Assessment of antigen sensitivity conveyed by lentivirally transduced NY-ESO-1-specific antigen receptor constructs. Population-based analysis of the calcium response of A2/NY-ESO-1-specific cells (of one donor) confronted with indicated A2/NY-ESO-1 variants at indicated densities (refer to **fig. S6**). Dashed lines indicate Fura-2 ratio threshold above which cells were considered activated (i.e., 1.25 for T1 $STAR_{nat}$ T-cells, T1 $STAR_{mir}$ T-cells and 1.15 for T1 BBz CAR T-cells).

Figure S8

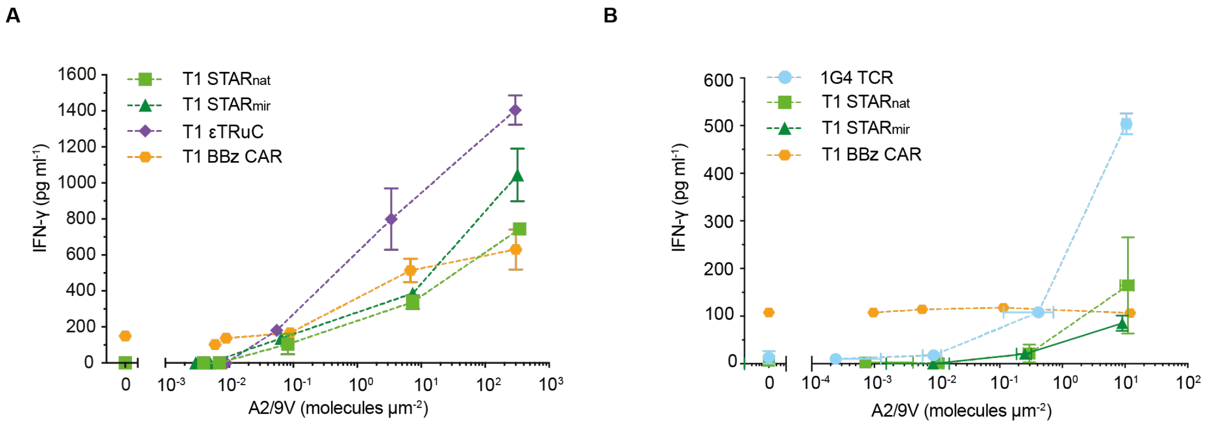
A
T1 STARnat
T1 STARmir
T1 εTRuC
T1 BBz CAR
IFN-γ (pg ml-1)
0
200
400
600
800
1000
1200
1400
1600
0
10-3
10-2
10-1
100
101
102
103
A2/9V (molecules µm-2)
B
1G4 TCR
T1 STARnat
T1 STARmir
T1 BBz CAR
IFN-γ (pg ml-1)
0
100
200
300
400
500
600
0
10-4
10-3
10-2
10-1
100
101
102
A2/9V (molecules µm-2)

Figure S8: IFN-γ secretion by engineered T-cells responding to SLBs decorated with ICAM-1 and antigen at indicated densities. IFN-γ secreted into the media of engineered T-cells stimulated via SLB-resident A2/9V present at indicated densities. A2/NY-ESO-1-specific constructs were introduced by means of lentiviral transduction (**A**) or CRISPR/Cas9-mediated knock in (**B**). Data were pooled from n=1 (A) and n=2 (B) biological replicates each containing n=2 technical duplicates. Statistics: Mean and s.e.m. of n=1-2 biological replicates.

Figure S9

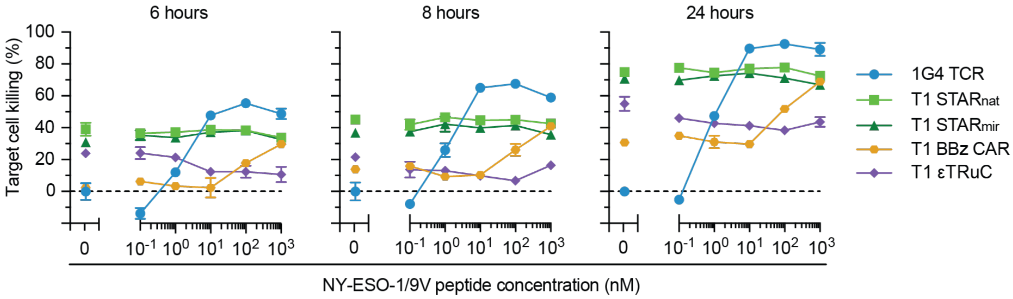
6 hours
8 hours
24 hours
Target cell killing (%)
100
80
60
40
20
0
0
10-1 10^0 10^1 10^2 10^3
NY-ESO-1/9V peptide concentration (nM)
1G4 TCR
T1 STARnat
T1 STARmir
T1 BBz CAR
T1 εTRuC

Figure S9: *Ex vivo* cytotoxic capacity of engineered T-cells with high antigen receptor expression levels. A2/NY-ESO-1-specific constructs (refer to **Fig. 3B-D**) were introduced by means of lentiviral transduction (T1 εTRuC, high expressors) or CRISPR/Cas9-mediated knock in (1G4 TCR, T1 $STAR_{nat}$, T1 $STAR_{mir}$, T1 BBz CAR). Indicated effector cells were cocultured at a ratio of 1:1 and for indicated times with HLA-A2-, CD80-, and luciferase-expressing K562 feeder cells, which had been pre-pulsed with the NY-ESO-1 peptide derivative 9V at indicated concentrations. Statistics: Mean ± s.e.m. from technical duplicates of one donor. Data are representative for n=2 biological replicates.

Table S1

Analyte	Ligand name	Peptide	Ka ($mol^{-1} s^{-1}$)	Kd (s^{-1})	KD (M)	Half-life (s)	Temp
RA14 TCR	A2/CMV	NLVPMVATV	9.60E+04	8.00E-01	8.30E-06	0.87	37 °C
1G4 TCR	A2/9V	SLLMWITQV	5.34E+04	7.80E-02	1.46E-06	8.89	37 °C
1G4hi TCR	A2/9V	SLLMWITQV	1.17E+06	8.26E-05	7.07E-11	8391.61	37 °C
	A2/6T	SLLMWTTQV	8.64E+05	7.14E-02	8.27E-08	9.71	37 °C
T1 scFv	A2/9V	SLLMWITQV	2.32E+05	4.60E-03	1.98E-08	150.68	37 °C
	A2/6T	SLLMWTTQV	1.64E+05	3.56E-02	2.17E-07	19.47	37 °C
	A2/4D	SLLDWITQV	1.09E+05	2.33E-01	2.14E-06	2.97	37 °C
FMC63 scFv	CD19		2.10E+05	6.80E-05	3.00E-08	10193.34	25 °C

Table S1. Binding constants for indicated receptor-ligand pairs at indicated temperatures as determined by surface plasmon resonance.

SUPPLEMENTARY MATERIAL:

Gene and protein sequences of constructs employed in this study

Data S1: c58c61 1G4hi TCR T2A copGFP (GenBank: PP746584)

Nucleotide sequence

```
   1 atggcttgtg gaagcgccac aatgtctatc ggcctgctgt gttgtgccgc tctgtctctg
  61 ctttgggccg gacctgttaa tgccggcgtg acccagacac ctaagttcca ggtgctgaaa
 121 accggccaga gcatgaccct gcagtgcgcc caggatatga accacgagta catgagctgg
 181 tacagacagg accctggcat gggcctgaga ctgatccact actctgtggc catccagacc
 241 accgacagag gcgaagtgcc caacggctac aacgtgtcca gaagcaccat cgaggacttc
 301 ccactgagac tgctgtctgc cgctcctagc cagaccagcg tgtacttttg tgccagcagc
 361 tacctgggca acaccggcga gctgtttttt ggcgagggca gcagactgac cgtgctggaa
 421 gatctgaaga acgtgttccc acctgaggtg gccgtgttcg agccttctga ggccgagatc
 481 agccacacac agaaagccac actcgtgtgt ctggccaccg gcttctatcc cgatcacgtg
 541 gaactgtctt ggtgggtcaa cggcaaagag gtgcacagcg gcgtcagcac agatccccag
 601 cctctgaaag aacagcccgc tctgaacgac agccggtact gtctgtcctc cagactgaga
 661 gtgtccgcca ccttctggca gaaccccaga aaccacttca gatgccaggt gcagttctac
 721 ggcctgagcg agaacgatga gtggacccag gatagagcca agcctgtgac acagatcgtg
 781 tctgccgaag cctggggcag agccgattgt ggctttacca gcgagagcta ccagcagggc
 841 gttctgtctg ccaccatcct gtacgagatc ctgctgggca aagccactct gtacgccgtg
 901 ctggtgtctg ccctggtgct gatggccatg gtcaagcgga aggacttttc tagaggcgcc
 961 gctgccggac cttctggacc tgatcctgcc accaatttca gcctgctgaa acaggccggc
1021 gacgtggaag agaaccccgg acctatggaa acactgctgg gcctgctgat cctgtggctg
1081 caactgcaat gggtgtcctc caagcaagaa gtgactcaga tcccagccgc tctgagtgtg
1141 cctgagggcg aaaacctggt cctgaactgc agcttcaccg acagcgccat ctacaacctg
1201 cagtggttca gacaagatcc cggcaaggga ctgacaagcc tgctgctgat taccccttgg
1261 cagagagagc agacctccgg cagactgaat gccagcctgg ataagagcag cggccggtct
1321 acactgtata tcgccgcttc tcagcctggc gatagcgcca catatctgtg tgccgtcaga
1381 cccctgctgg acggcacata tatccctacc tttggcagag gcaccagcct gatcgtgcac
1441 ccctacattc agaaccccga tcctgccgtg tatcagctga gagacagcaa gtccagcgac
1501 aagagcgtgt gcctgttcac cgacttcgac tcccagacca atgtgtccca gagcaaggac
1561 agcgacgtgt acatcaccga caagacagtg ctggacatgc ggagcatgga cttcaagagc
1621 aacagcgccg tggcctggtc caacaagagc gatttcgcct gcgccaacgc cttcaacaac
1681 agcattatcc ccgaggacac attcttccca agtcctgaga gcagctgcga cgtgaagctg
1741 gtggaaaaga gcttcgagac agacaccaac ctgaacttcc agaacctgag cgtgatcggc
1801 ttccggattc tgctgctcaa ggtggccggc ttcaacctgc tgatgaccct gagactctgg
1861 tccagcgaat tcgaaggatc cgcggccgct gagggcagag gaagtcttct aacatgcggt
1921 gacgtggagg agaatcccgg cccttccgga atggagagcg acgagagcgg cctgcccgcc
1981 atggagatcg agtgccgcat caccggcacc ctgaacggcg tggagttcga gctggtgggc
```

2041 ggcggagagg gcaccccaa gcagggccgc atgaccaaca agatgaagag caccaaaggc
2101 gccctgacct tcagccccta cctgctgagc cacgtgatgg gctacggctt ctaccacttc
2161 ggcacctacc ccagcggcta cgagaacccc ttcctgcacg ccatcaacaa cggcggctac
2221 accaacaccc gcatcgagaa gtacgaggac ggcggcgtgc tgcacgtgag cttcagctac
2281 cgctacgagg ccggccgcgt gatcggcgac ttcaaggtgg tgggcaccgg cttccccgag
2341 gacagcgtga tcttcaccga caagatcatc cgcagcaacg ccaccgtgga gcacctgcac
2401 cccatgggcg ataacgtgct ggtgggcagc ttcgcccgca ccttcagcct gcgcgacggc
2461 ggctactaca gcttcgtggt ggacagccac atgcacttca agagcgccat ccaccccagc
2521 atcctgcaga acgggggccc catgttcgcc ttccgccgcg tggaggagct gcacagcaac
2581 accgagctgg gcatcgtgga gtaccagcac gccttcaaga cccccatcgc cttcgccaga
2641 tcccgcgctc agtcgtccaa ttctgccgtg gacggcaccg ccggacccgg ctccaccgga
2701 tctcgctag

Protein sequence:

SP TCRβ P2A SP TCRα T2A GFP

1 11 21 31 41 51
MACGSATMSI GLLCCAALSL LWAGPVNAGV TQTPKFQVLK TGQSMTLQCA QDMNHEYMSW
61 71 81 91 101 111
YRQDPGMGLR LIHYSVAIQT TDRGEVPNGY NVSRSTIEDF PLRLLSAAPS QTSVYFCASS
121 131 141 151 161 171
YLGNTGELFF GEGSRLTVLE DLKNVFPPEV AVFEPSEAEI SHTQKATLVC LATGFYPDHV
181 191 201 211 221 231
ELSWWVNGKE VHSGVSTDPQ PLKEQPALND SRYCLSSRLR VSATFWQNPR NHFRCQVQFY
241 251 261 271 281 291
GLSENDEWTQ DRAKPVTQIV SAEAWGRADC GFTSESYQQG VLSATILYEI LLGKATLYAV
301 311 321 331 341 351
LVSALVLMAM VKRKDFSRGA AAGPSGPDPA TNFSLLKQAG DVEENPGPME TLLGLLILWL
361 371 381 391 401 411
QLQWVSSKQE VTQIPAALSV PEGENLVLNC SFTDSAIYNL QWFRQDPGKG LTSLLLITPW
421 431 441 451 461 471
QREQTSGRLN ASLDKSSGRS TLYIAASQPG DSATYLCAVR PLLDGTYIPT FGRGTSLIVH
481 491 501 511 521 531
PYIQNPDPAV YQLRDSKSSD KSVCLFTDFD SQTNVSQSKD SDVYITDKTV LDMRSMDFKS
541 551 561 571 581 591
NSAVAWSNKS DFACANAFNN SIIPEDTFFP SPESSCDVKL VEKSFETDTN LNFQNLSVIG
601 611 621 631 641 651
FRILLLLKVAG FNLLMTLRLW SSEFEGSAAA EGRGSLLTCG DVEENPGPSG MESDESGLPA
661 671 681 691 701 711
MEIECRITGT LNGVEFELVG GGEGTPKQGR MTNKMKSTKG ALTFSPYLLS HVMGYGFYHF

721	731	741	751	761	771
GTYPSGYENP	FLHAINNGGY	TNTRIEKYED	GGVLHVSFSY	RYEAGRVIGD	FKVVGTGFPE
781	791	801	811	821	831
DSVIFTDKII	RSNATVEHLH	PMGDNVLVGS	FARTFSLRDG	GYYSFVVDSH	MHFKSAIHPS
841	851	861	871	881	891
ILQNGGPMFA	FRRVEELHSN	TELGIVEYQH	AFKTPIAFAR	SRAQSSNSAV	DGTAGPGSTG
901					
SR					

Data S2: T1 STAR$_{nat}$ T2A copGFP (GenBank: PP746585)

Nucleotide sequence

```
   1 atgggcagct ggaccctgtg ttgcgtgtcc ctgtgcatcc tggtggctaa gcacacccag
  61 agcgagctga cccagcccag aagcgtgtcc ggcagccctg gccagagcgt gaccatcagc
 121 tgtaccggca ccgaaagaga tgtgggcggc tacaactacg tgtcctggta tcagcagcac
 181 cccggcaagg cccctaagct gatcatccac gacgtgatcg agagaagcag cggcgtgccc
 241 gacagattca gcggcagcaa gagcggcaac accgccagcc tgaccatctc tggcctccag
 301 gccgaggacg aggccgacta ctactgctgg agcttcgccg gcggatacta cgtgttcggc
 361 accggcaccg acgtgaccgt ggaggatctg agaaatgtga ctccacccaa ggtctccttg
 421 tttgagccat caaaagcaga gattgcaaac aaacaaaagg ctaccctcgt gtgcttggcc
 481 aggggcttct tccctgacca cgtggagctg agctggtggg tgaatggcaa ggaggtccac
 541 agtggggtct gcacggaccc tcaggcctac aaggagagca attatagcta ctgcctgagc
 601 agccgcctga gggtctctgc taccttctgg cacaatcctc gaaaccactt ccgctgccaa
 661 gtgcagttcc atgggctttc agaggaggac aagtggccag agggctcacc caaacctgtc
 721 acacagaaca tcagtgcaga ggcctggggc cgagcagact gtggaatcac ttcagcatcc
 781 tatcatcagg gggttctgtc tgcaaccatc ctctatgaga tcctactggg gaaggccacc
 841 ctatatgctg tgctggtcag tggcctggtg ctgatggcca tggtcaagaa aaaaaattcc
 901 ggcagcggcg agggcagagg aagtctgcta acatgcggtg acgtcgagga gaatcctgga
 961 cctatgatga agtccctgag ggtgctgctg gtgatcctct ggctgcagct gagctgggtg
1021 tggtcccagg aggtgcagct gctggagtct ggcggcggac tggtgcagcc tggcggcagc
1081 ctgagactga gctgtgccgc cagcggcttc accttcagca cctaccagat gagctgggtg
1141 cggcaggccc ctggcaaggg cctggagtgg gtgtccggca tcgtgtccag cggcggcagc
1201 accgcctacg ccgacagcgt gaagggcagg ttcaccatca gccgggacaa cagcaagaac
1261 accctgtacc tgcagatgaa cagcctgaga gccgaggaca ccgccgtgta ctactgtgcc
1321 ggcgagctgc tgccctacta cggcatggat gtgtggggcc agggcaccac cgtgacagtg
1381 agcatccaga acccagaacc tgctgtgtac cagttaaaag atcctcggtc tcaggacagc
1441 accctctgcc tgttcaccga ctttgactcc caaatcaatg tgccgaaaac catggaatct
1501 ggaacgttca tcactgacaa atgcgtgctg gacatgaaag ctatggattc caagagcaat
1561 ggggccattg cctggagcaa ccagacaagc ttcacctgcc aagatatctt caaagagacc
1621 aacgccacct accccagttc agacgttccc tgtgatgcca cgttgactga gaaaagcttt
1681 gaaacagata tgaacctaaa ctttcaaaac ctgtcagtta tgggactccg aatcctcctg
1741 ctgaaagtag ccggatttaa cctgctcatg acgctgaggc tgtggtccag tgaattcgaa
1801 ggatccgcgg ccgctgaggg cagaggaagt cttctaacat gcggtgacgt ggaggagaat
1861 cccggccctt ccggaatgga gagcgacgag agcggcctgc ccgccatgga gatcgagtgc
1921 cgcatcaccg gcaccctgaa cggcgtggag ttcgagctgg tgggcggcgg agagggcacc
1981 cccaagcagg gccgcatgac caacaagatg aagagcacca aaggcgccct gaccttcagc
2041 ccctacctgc tgagccacgt gatgggctac ggcttctacc acttcggcac ctaccccagc
2101 ggctacgaga acccctteet gcacgccatc aacaacggcg gctacaccaa cacccgcatc
2161 gagaagtacg aggacggcgg cgtgctgcac gtgagcttca gctaccgcta cgaggccggc
```

2221 cgcgtgatcg gcgacttcaa ggtggtgggc accggcttcc ccgaggacag cgtgatcttc
2281 accgacaaga tcatccgcag caacgccacc gtggagcacc tgcaccccat gggcgataac
2341 gtgctggtgg gcagcttcgc ccgcaccttc agcctgcgcg acggcggcta ctacagcttc
2401 gtggtggaca gccacatgca cttcaagagc gccatccacc ccagcatcct gcagaacggg
2461 ggccccatgt tcgccttccg ccgcgtggag gagctgcaca gcaacaccga gctgggcatc
2521 gtggagtacc agcacgcctt caagaccccc atcgccttcg ccagatcccg cgctcagtcg
2581 tccaattctg ccgtggacgg caccgccgga cccggctcca ccggatctcg ctag

Protein sequence

SP T1 V_L mCβ T2A SP T1 V_H mCα T2A GFP

1	11	21	31	41	51
MGSWTLCCVS	LCILVAKHTQ	SELTQPRSVS	GSPGQSVTIS	CTGTERDVGG	YNYVSWYQQH
61	71	81	91	101	111
PGKAPKLIIH	DVIERSSGVP	DRFSGSKSGN	TASLTISGLQ	AEDEADYYCW	SFAGGYYVFG
121	131	141	151	161	171
TGTDVTVEDL	RNVTPPKVSL	FEPSKAEIAN	KQKATLVCLA	RGFFPDHVEL	SWWVNGKEVH
181	191	201	211	221	231
SGVCTDPQAY	KESNYSYCLS	SRLRVSATFW	HNPRNHFRCQ	VQFHGLSEED	KWPEGSPKPV
241	251	261	271	281	291
TQNISAEAWG	RADCGITSAS	YHQGVLSATI	LYEILLGKAT	LYAVLVSGLV	LMAMVKKKNS
301	311	321	331	341	351
GSGEGRGSLL	TCGDVEENPG	PMMKSLRVLL	VILWLQLSWV	WSQEVQLLES	GGGLVQPGGS
361	371	381	391	401	411
LRLSCAASGF	TFSTYQMSWV	RQAPGKGLEW	VSGIVSSGGS	TAYADSVKGR	FTISRDNSKN
421	431	442	451	461	471
TLYLQMNSLR	AEDTAVYYCA	GELLPYYGMD	VWGQGTTVTV	SIQNPEPAVY	QLKDPRSQDS
481	491	501	511	521	531
TLCLFTDFDS	QINVPKTMES	GTFITDKCVL	DMKAMDSKSN	GAIAWSNQTS	FTCQDIFKET
541	551	561	571	581	591
NATYPSSDVP	CDATLTEKSF	ETDMNLNFQN	LSVMGLRILL	LKVAGFNLLM	TLRLWSSEFE
601	611	621	631	641	651
GSAAAEGRGS	LLTCGDVEEN	PGPSGMESDE	SGLPAMEIEC	RITGTLNGVE	FELVGGGEGT
661	671	681	691	701	711
PKQGRMTNKM	KSTKGALTFS	PYLLSHVMGY	GFYHFGTYPS	GYENPFLHAI	NNGGYTNTRI
721	731	741	751	761	771
EKYEDGGVLH	VSFSYRYEAG	RVIGDFKVVG	TGFPEDSVIF	TDKIIRSNAT	VEHLHPMGDN
781	791	801	811	821	831
VLVGSFARTF	SLRDGGYYSF	VVDSHMHFKS	AIHPSILQNG	GPMFAFRRVE	ELHSNTELGI
841	851	861	871 877		

VEYQHAFKTP IAFARSRAQS SNSAVDGTAG PGSTGSR

Data S3: T1 $STAR_{mir}$ T2A copGFP (GenBank: PP746586)

Nucleotide sequence

```
   1 atgggcagct ggaccctgtg ttgcgtgtcc ctgtgcatcc tggtggctaa gcacaccgag
  61 gtgcagctgc tggagtctgg cggcggactg gtgcagcctg gcggcagcct gagactgagc
 121 tgtgccgcca gcggcttcac cttcagcacc taccagatga gctgggtgcg gcaggcccct
 181 ggcaagggcc tggagtgggt gtccggcatc gtgtccagcg gcggcagcac cgcctacgcc
 241 gacagcgtga agggcaggtt caccatcagc cgggacaaca gcaagaacac cctgtacctg
 301 cagatgaaca gcctgagagc cgaggacacc gccgtgtact actgtgccgg cgagctgctg
 361 ccctactacg gcatggatgt gtggggccag ggcaccaccg tgacagtgag cgaggatctg
 421 agaaatgtga ctccacccaa ggtctccttg tttgagccat caaaagcaga gattgcaaac
 481 aaacaaaagg ctaccctcgt gtgcttggcc aggggcttct tccctgacca cgtggagctg
 541 agctggtggg tgaatggcaa ggaggtccac agtggggtct gcacggaccc tcaggcctac
 601 aaggagagca attatagcta ctgcctgagc agccgcctga gggtctctgc taccttctgg
 661 cacaatcctc gaaaccactt ccgctgccaa gtgcagttcc atgggctttc agaggaggac
 721 aagtggccag agggctcacc caaacctgtc acacagaaca tcagtgcaga ggcctggggc
 781 cgagcagact gtggaatcac ttcagcatcc tatcatcagg gggttctgtc tgcaaccatc
 841 ctctatgaga tcctactggg gaaggccacc ctatatgctg tgctggtcag tggcctggtg
 901 ctgatggcca tggtcaagaa aaaaaattcc ggcagcggcg agggcagagg aagtctgcta
 961 acatgcggtg acgtcgagga gaatcctgga cctatgatga agtccctgag ggtgctgctg
1021 gtgatcctct ggctgcagct gagctgggtg tggtcccagc agagcgagct gacccagccc
1081 agaagcgtgt ccggcagccc tggccagagc gtgaccatca gctgtaccgg caccgaaaga
1141 gatgtgggcg gctacaacta cgtgtcctgg tatcagcagc accccggcaa ggcccctaag
1201 ctgatcatcc acgacgtgat cgagagaagc agcggcgtgc ccgacagatt cagcggcagc
1261 aagagcggca acaccgccag cctgaccatc tctggcctcc aggccgagga cgaggccgac
1321 tactactgct ggagcttcgc cggcggatac tacgtgttcg gcaccggcac cgacgtgacc
1381 gtgatccaga acccagaacc tgctgtgtac cagttaaaag atcctcggtc tcaggacagc
1441 accctctgcc tgttcaccga ctttgactcc caaatcaatg tgccgaaaac catggaatct
1501 ggaacgttca tcactgacaa atgcgtgctg gacatgaaag ctatggattc caagagcaat
1561 ggggccattg cctggagcaa ccagacaagc ttcacctgcc aagatatctt caaagagacc
1621 aacgccacct accccagttc agacgttccc tgtgatgcca cgttgactga gaaaagcttt
1681 gaaacagata tgaacctaaa ctttcaaaac ctgtcagtta tgggactccg aatcctcctg
1741 ctgaaagtag ccggatttaa cctgctcatg acgctgaggc tgtggtccag tgaattcgaa
1801 ggatccgcgg ccgctgaggg cagaggaagt cttctaacat gcggtgacgt ggaggagaat
1861 cccggccctt ccggaatgga gagcgacgag agcggcctgc ccgccatgga gatcgagtgc
1921 cgcatcaccg gcaccctgaa cggcgtggag ttcgagctgg tgggcggcgg agagggcacc
1981 cccaagcagg gccgcatgac caacaagatg aagagcacca aaggcgccct gaccttcagc
2041 ccctacctgc tgagccacgt gatgggctac ggcttctacc acttcggcac ctaccccagc
2101 ggctacgaga acccctteet gcacgccatc aacaacggcg gctacaccaa cacccgcatc
2161 gagaagtacg aggacggcgg cgtgctgcac gtgagcttca gctaccgcta cgaggccggc
```

2221 cgcgtgatcg gcgacttcaa ggtggtgggc accggcttcc ccgaggacag cgtgatcttc
2281 accgacaaga tcatccgcag caacgccacc gtggagcacc tgcaccccat gggcgataac
2341 gtgctggtgg gcagcttcgc ccgcaccttc agcctgcgcg acggcggcta ctacagcttc
2401 gtggtggaca gccacatgca cttcaagagc gccatccacc ccagcatcct gcagaacggg
2461 ggccccatgt tcgccttccg ccgcgtggag gagctgcaca gcaacaccga gctgggcatc
2521 gtggagtacc agcacgcctt caagaccccc atcgccttcg ccagatcccg cgctcagtcg
2581 tccaattctg ccgtggacgg caccgccgga cccggctcca ccggatctcg ctag

Protein sequence:

SP T1 V_H mCβ T2A SP T1 V_L mCα T2A GFP

1 11 21 31 41 51
MGSWTLCCVS LCILVAKHTE VQLLESGGGL VQPGGSLRLS CAASGFTFST YQMSWVRQAP
61 71 81 91 101 111
GKGLEWVSGI VSSGGSTAYA DSVKGRFTIS RDNSKNTLYL QMNSLRAEDT AVYYCAGELL
121 131 141 151 161 171
PYYGMDVWGQ GTTVTVSEDL RNVTPPKVSL FEPSKAEIAN KQKATLVCLA RGFFPDHVEL
181 191 201 211 221 231
SWWVNGKEVH SGVCTDPQAY KESNYSYCLS SRLRVSATFW HNPRNHFRCQ VQFHGLSEED
241 251 261 271 281 291
KWPEGSPKPV TQNISAEAWG RADCGITSAS YHQGVLSATI LYEILLGKAT LYAVLVSGLV
301 311 321 331 341 351
LMAMVKKKNS GSGEGRGSLL TCGDVEENPG PMMKSLRVLL VILWLQLSWV WSQQSELTQP
361 371 381 391 401 411
RSVSGSPGQS VTISCTGTER DVGGYNYVSW YQQHPGKAPK LIIHDVIERS SGVPDRFSGS
421 431 442 451 461 471
KSGNTASLTI SGLQAEDEAD YYCWSFAGGY YVFGTGTDVT VIQNPEPAVY QLKDPRSQDS
481 491 501 511 521 531
TLCLFTDFDS QINVPKTMES GTFITDKCVL DMKAMDSKSN GAIAWSNQTS FTCQDIFKET
541 551 561 571 581 591
NATYPSSDVP CDATLTEKSF ETDMNLNFQN LSVMGLRILL LKVAGFNLLM TLRLWSSEFE
601 611 621 631 641 651
GSAAAEGRGS LLTCGDVEEN PGPSGMESDE SGLPAMEIEC RITGTLNGVE FELVGGGEGT
661 671 681 691 701 711
PKQGRMTNKM KSTKGALTFS PYLLSHVMGY GFYHFGTYPS GYENPFLHAI NNGGYTNTRI
721 731 741 751 761 771
EKYEDGGVLH VSFSYRYEAG RVIGDFKVVG TGFPEDSVIF TDKIIRSNAT VEHLHPMGDN
781 791 801 811 821 831
VLVGSFARTF SLRDGGYYSF VVDSHMHFKS AIHPSILQNG GPMFAFRRVE ELHSNTELGI
841 851 861 871 877

VEYQHAFKTP IAFARSRAQS SNSAVDGTAG PGSTGSR

Data S4: T1 εTRuC T2A copGFP (GenBank: PP746587)

Nucleotide sequence

```
   1 atgctgctgc tggtcacctc tctgctgctg tgcgagctgc cccaccccgc ctttctgctg
  61 atccccatgg cggaggtgca gctgctggag tctggcggcg gactggtgca gcctggcggc
 121 agcctgagac tgagctgtgc cgccagcggc ttcaccttca gcacctacca gatgagctgg
 181 gtgcggcagg cccctggcaa gggcctggag tgggtgtccg gcatcgtgtc cagcggcggc
 241 agcaccgcct acgccgacag cgtgaagggc aggttcacca tcagccggga caacagcaag
 301 aacaccctgt acctgcagat gaacagcctg agagccgagg acaccgccgt gtactactgt
 361 gccggcgagc tgctgcccta ctacggcatg gatgtgtggg gccagggcac caccgtgaca
 421 gtgagcagcg ccaagaccac ccccaagctg gaggagggcg agttcagcga ggccagagtg
 481 cagagcgagc tgacccagcc cagaagcgtg tccggcagcc ctggccagag cgtgaccatc
 541 agctgtaccg gcaccgaaag agatgtgggc ggctacaact acgtgtcctg gtatcagcag
 601 caccccggca aggcccctaa gctgatcatc cacgacgtga tcgagagaag cagcggcgtg
 661 cccgacagat tcagcggcag caagagcggc aacaccgcca gcctgaccat ctctggcctc
 721 caggccgagg acgaggccga ctactactgc tggagcttcg ccggcggata ctacgtgttc
 781 ggcaccggca ccgacgtgac cgtgctgggc cagcccaagg ccaaccccac aggaggcggt
 841 ggttctggtg gcggaggaag tggcggcgga ggatccctcg aggatggcaa cgaggaaatg
 901 ggcggcatca cccagacccc ttacaaggtg tccatcagcg gcaccaccgt gatcctgacc
 961 tgccctcagt accccggctc cgagatcctg tggcagcata acgacaagaa catcggcggc
1021 gacgaggacg ataagaatat cggctccgat gaggaccacc tgagcctgaa agagttcagc
1081 gagctggaac agagcggcta ctacgtgtgc taccccagag gcagcaagcc cgaggacgcc
1141 aacttctacc tgtacctgcg ggccagagtg tgcgagaact gcatggaaat ggacgtgatg
1201 agcgtggcca ccatcgtgat cgtggacatc tgcatcaccg gcggactgct gctcctcgtg
1261 tactactggt ccaagaaccg gaaggccaag gccaagcctg tgacaagagg tgctggtgcc
1321 ggcggaaggc agcggggcca gaacaaagaa agacctcctc ccgtgcccaa ccccgactac
1381 gagcccatca gaaagggaca gcgggacctg tacagcggcc tgaaccagcg gagaatcgaa
1441 ttcgaaggat ccgcggccgc tgagggcaga ggaagtcttc taacatgcgg tgacgtggag
1501 gagaatcccg gcccttccgg aatggagagc gacgagagcg gcctgcccgc catggagatc
1561 gagtgccgca tcaccggcac cctgaacggc gtggagttcg agctggtggg cggcggagag
1621 ggcaccccca agcagggccg catgaccaac aagatgaaga gcaccaaagg cgccctgacc
1681 ttcagcccct acctgctgag ccacgtgatg ggctacggct tctaccactt cggcacctac
1741 cccagcggct acgagaaccc cttcctgcac gccatcaaca acggcggcta caccaacacc
1801 cgcatcgaga agtacgagga cggcggcgtg ctgcacgtga gcttcagcta ccgctacgag
1861 gccggccgcg tgatcggcga cttcaaggtg gtgggcaccg gcttccccga ggacagcgtg
1921 atcttcaccg acaagatcat ccgcagcaac gccaccgtgg agcacctgca ccccatgggc
1981 gataacgtgc tggtgggcag cttcgcccgc accttcagcc tgcgcgacgg cggctactac
2041 agcttcgtgg tggacagcca catgcacttc aagagcgcca tccaccccag catcctgcag
2101 aacgggggcc ccatgttcgc cttccgccgc gtggaggagc tgcacagcaa caccgagctg
2161 ggcatcgtgg agtaccagca cgccttcaag acccccatcg ccttcgccag atcccgcgct
```

2221 cagtcgtcca attctgccgt ggacggcacc gccggacccg gctccaccgg atctcgctag

Protein sequence:

SP T1 scF$_V$ linker CD3ε T2A GFP

1 11 21 31 41 51

MLLLVTSLLL CELPHPAFLL IPMAEVQLLE SGGGLVQPGG SLRLSCAASG FTFSTYQMSW

61 71 81 91 101 111

VRQAPGKGLE WVSGIVSSGG STAYADSVKG RFTISRDNSK NTLYLQMNSL RAEDTAVYYC

121 131 141 151 161 171

AGELLPYYGM DVWGQGTTVT VSSAKTTPKL EEGEFSEARV QSELTQPRSV SGSPGQSVTI

181 191 201 211 221 231

SCTGTERDVG GYNYVSWYQQ HPGKAPKLII HDVIERSSGV PDRFSGSKSG NTASLTISGL

241 251 261 271 281 291

QAEDEADYYC WSFAGGYYVF GTGTDVTVLG QPKANPTGGG GSGGGGSGGG GSLEDGNEEM

301 311 321 331 341 351

GGITQTPYKV SISGTTVILT CPQYPGSEIL WQHNDKNIGG DEDDKNIGSD EDHLSLKEFS

361 371 381 391 401 411

ELEQSGYYVC YPRGSKPEDA NFYLYLRARV CENCMEMDVM SVATIVIVDI CITGGLLLLV

421 431 442 451 461 471

YYWSKNRKAK AKPVTRGAGA GGRQRGQNKE RPPPVPNPDY EPIRKGQRDL YSGLNQRRIE

481 491 501 511 521 531

FEGSAAAEGR GSLLTCGDVE ENPGPSGMES DESGLPAMEI ECRITGTLNG VEFELVGGGE

541 551 561 571 581 591

GTPKQGRMTN KMKSTKGALT FSPYLLSHVM GYGFYHFGTY PSGYENPFLH AINNGGYTNT

601 611 621 631 641 651

RIEKYEDGGV LHVSFSYRYE AGRVIGDFKV VGTGFPEDSV IFTDKIIRSN ATVEHLHPMG

661 671 681 691 701 711

DNVLVGSFAR TFSLRDGGYY SFVVDSHMHF KSAIHPSILQ NGGPMFAFRR VEELHSNTEL

721 731 741 751 759

GIVEYQHAFK TPIAFARSRA QSSNSAVDGT AGPGSTGSR

Data S5: T1 BBz CAR T2A copGFP (GenBank: PP746588)

Nucleotide sequence

```
       1 atgcttctcc tggtgacaag ccttctgctc tgtgagttac cacacccagc attcctcctg
      61 atcccaatgg cggaggtgca gctgctggag tctggcggcg gactggtgca gcctggcggc
     121 agcctgagac tgagctgtgc cgccagcggc ttcaccttca gcacctacca gatgagctgg
     181 gtgcggcagg cccctggcaa gggcctggag tgggtgtccg gcatcgtgtc cagcggcggc
     241 agcaccgcct acgccgacag cgtgaagggc aggttcacca tcagccggga caacagcaag
     301 aacaccctgt acctgcagat gaacagcctg agagccgagg acaccgccgt gtactactgt
     361 gccggcgagc tgctgcccta ctacggcatg gatgtgtggg gccagggcac caccgtgaca
     421 gtgagcagcg ccaagaccac ccccaagctg gaggagggcg agttcagcga ggccagagtg
     481 cagagcgagc tgacccagcc cagaagcgtg tccggcagcc ctggccagag cgtgaccatc
     541 agctgtaccg gcaccgaaag agatgtgggc ggctacaact acgtgtcctg gtatcagcag
     601 caccccggca aggcccctaa gctgatcatc cacgacgtga tcgagagaag cagcggcgtg
     661 cccgacagat tcagcggcag caagagcggc aacaccgcca gcctgaccat ctctggcctc
     721 caggccgagg acgaggccga ctactactgc tggagcttcg ccggcggata ctacgtgttc
     781 ggcaccggca ccgacgtgac cgtgctgggc cagcccaagg ccaaccccac actcgagaat
     841 tggtcacatc ctcaatttga aaaaggtgga ggcggttcac ccgggacaac cacccctgcc
     901 cctagacctc ccaccccagc cccaacaatt gccagccagc ctctgtctct gcggcccgaa
     961 gcttgtagac ctgctgccgg cggagccgtg cacaccagag gactggattt cgcctgcgac
    1021 atctacatct gggcccctct ggccggcaca tgtggcgtgc tgctcctcag cctggtcatc
    1081 accctgtact gcaagcgggg cagaaagaaa ctgctctaca tcttcaagca gcccttcatg
    1141 cggcccgtgc agaccacaca ggaagaggac ggctgctcct gcagattccc cgaggaagaa
    1201 gaaggcggct gcgagctgag agtgaagttc agcagatccg ccgacgcccc tgcctaccag
    1261 cagggacaga accagctgta caacgagctg aacctgggca gacgggaaga gtacgacgtg
    1321 ctggacaagc ggagaggcag agatcccgag atgggcggca agcccagacg gaagaatccc
    1381 caggaaggcc tgtataacga actgcagaaa gacaagatgg ccgaggccta cagcgagatc
    1441 ggaatgaagg gcgagcggag aagaggcaag ggccacgatg gcctgtacca gggcctgagc
    1501 accgccacca aggacaccta cgatgccctg cacatgcagg ccctgccacc cagagaattc
    1561 gaaggatccg cggccgctga gggcagagga agtcttctaa catgcggtga cgtggaggag
    1621 aatcccggcc cttccggaat ggagagcgac gagagcggcc tgcccgccat ggagatcgag
    1681 tgccgcatca ccggcaccct gaacggcgtg gagttcgagc tggtgggcgg cggagagggc
    1741 acccccaagc agggccgcat gaccaacaag atgaagagca ccaaaggcgc cctgaccttc
    1801 agcccctacc tgctgagcca cgtgatgggc tacggcttct accacttcgg cacctacccc
    1861 agcggctacg agaacccctt cctgcacgcc atcaacaacg gcggctacac caacacccgc
    1921 atcgagaagt acgaggacgg cggcgtgctg cacgtgagct tcagctaccg ctacgaggcc
    1981 ggccgcgtga tcggcgactt caaggtggtg ggcaccggct tccccgagga cagcgtgatc
    2041 ttcaccgaca agatcatccg cagcaacgcc accgtggagc acctgcaccc catgggcgat
    2101 aacgtgctgg tgggcagctt cgcccgcacc ttcagcctgc gcgacggcgg ctactacagc
    2161 ttcgtggtgg acagccacat gcacttcaag agcgccatcc accccagcat cctgcagaac
```

2221 gggggcccca tgttcgcctt ccgccgcgtg gaggagctgc acagcaacac cgagctgggc

2281 atcgtggagt accagcacgc cttcaagacc cccatcgcct tcgccagatc ccgcgctcag

2341 tcgtccaatt ctgccgtgga cggcaccgcc ggacccggct ccaccggatc tcgctag

Protein sequence

SP T1 scF$_V$ CD8 stalk/CD28 TM/4-1BB CyT CD3ζ CyT T2A GFP

1 11 21 31 41 51

MLLLVTSLLL CELPHPAFLL IPMAEVQLLE SGGGLVQPGG SLRLSCAASG FTFSTYQMSW

61 71 81 91 101 111

VRQAPGKGLE WVSGIVSSGG STAYADSVKG RFTISRDNSK NTLYLQMNSL RAEDTAVYYC

121 131 141 151 161 171

AGELLPYYGM DVWGQGTTVT VSSAKTTPKL EEGEFSEARV QSELTQPRSV SGSPGQSVTI

181 191 201 211 221 231

SCTGTERDVG GYNYVSWYQQ HPGKAPKLII HDVIERSSGV PDRFSGSKSG NTASLTISGL

241 251 261 271 281 291

QAEDEADYYC WSFAGGYYVF GTGTDVTVLG QPKANPTLEN WSHPQFEKGG GGSPGTTTPA

301 311 321 331 341 351

PRPPTPAPTI ASQPLSLRPE ACRPAAGGAV HTRGLDFACD IYIWAPLAGT CGVLLLSLVI

361 371 381 391 401 411

TLYCKRGRKK LLYIFKQPFM RPVQTTQEED GCSCRFPEEE EGGCELRVKF SRSADAPAYQ

421 431 442 451 461 471

QGQNQLYNEL NLGRREEYDV LDKRRGRDPE MGGKPRRKNP QEGLYNELQK DKMAEAYSEI

481 491 501 511 521 531

GMKGERRRGK GHDGLYQGLS TATKDTYDAL HMQALPPREF EGSAAAEGRG SLLTCGDVEE

541 551 561 571 581 591

NPGPSGMESD ESGLPAMEIE CRITGTLNGV EFELVGGGEG TPKQGRMTNK MKSTKGALTF

601 611 621 631 641 651

SPYLLSHVMG YGFYHFGTYP SGYENPFLHA INNGGYTNTR IEKYEDGGVL HVSFSYRYEA

661 671 681 691 701 711

GRVIGDFKVV GTGFPEDSVI FTDKIIRSNA TVEHLHPMGD NVLVGSFART FSLRDGGYYS

721 731 741 751 761 771

FVVDSHMHFK SAIHPSILQN GGPMFAFRRV EELHSNTELG IVEYQHAFKT PIAFARSRAQ

781 791 798

SSNSAVDGTA GPGSTGSR

Data S6: 1G4wt TCR CRISPR construct (GenBank: PP746589)

Nucleotide sequence

```
   1 ctgcctttac tctgccagag ttatattgct ggggttttga agaagatcct attaaataaa
  61 agaataagca gtattattaa gtagccctgc atttcaggtt tccttgagtg gcaggccagg
 121 cctggccgtg aacgttcact gaaatcatgg cctcttggcc aagattgata gcttgtgcct
 181 gtccctgagt cccagtccat cacgagcagc tggtttctaa gatgctattt cccgtataaa
 241 gcatgagacc gtgacttgcc agccccacag agccccgccc ttgtccatca ctggcatctg
 301 gactccagcc tgggttgggg caaagaggga aatgagatca tgtcctaacc ctgatcctct
 361 tgtcccacag atatccagaa ccctgaccct gccgtgggca gcggcgccac caacttcagc
 421 ctgctgaagc aggccggcga cgtggaagag aaccccgggc ccatgagcat cggcctcctg
 481 tgctgtgcag ccttgtctct cctgtgggca ggtccagtga atgctggtgt cactcagacc
 541 ccaaaattcc aggtcctgaa gacaggacag agcatgacac tgcagtgtgc ccaggatatg
 601 aaccatgaat acatgtcctg gtatcgacaa gacccaggca tggggctgag gctgattcat
 661 tactcagttg gtgctggtat cactgaccaa ggagaagtcc ccaatggcta caatgtctcc
 721 agatcaacca cagaggattt cccgctcagg ctgctgtcgg ctgctccctc ccagacatct
 781 gtgtacttct gtgccagcag ttacgtcggg aacaccgggg agctgttttt tggagaaggc
 841 tctaggctga ccgtactgga ggacctgaaa aacgtgttcc cacccgaggt cgctgtgttt
 901 gagccatcag aagcagagat ctcccacacc caaaaggcca cactggtgtg cctggccaca
 961 ggcttctacc ccgaccacgt ggagctgagc tggtgggtga atgggaagga ggtgcacagt
1021 ggggtcagca cagacccgca gcccctcaag gagcagcccg ccctcaatga ctccagatac
1081 tgcctgagca gccgcctgag ggtctcggcc accttctggc agaacccccg caaccacttc
1141 cgctgtcaag tccagttcta cgggctctcg gagaatgacg agtggaccca agatagggcc
1201 aaacctgtca cccagatcgt cagcgccgag gcctggggta gagcagactg tggcttcacc
1261 tccgagtctt accagcaagg ggtcctgtct gccaccatcc tctatgagat cttgctaggg
1321 aaggccacct tgtatgccgt gctggtcagt gccctcgtgc tgatggccat ggtcaagaga
1381 aaggattcca gaggcggcag cggcgagggc agaggaagtc tgctaacatg cggtgacgtc
1441 gaggagaatc ctggacctat ggagaccctc ttgggcctgc ttatcctttg gctgcagctg
1501 caatgggtga gcagcaaaca ggaggtgaca cagattcctg cagctctgag tgtcccagaa
1561 ggagaaaact tggttctcaa ctgcagtttc actgatagcg ctatttacaa cctccagtgg
1621 tttaggcagg accctgggaa aggtctcaca tctctgttgc ttattcagtc aagtcagaga
1681 gagcaaacaa gtggaagact taatgcctcg ctggataaat catcaggacg tagtacttta
1741 tacattgcag cttctcagcc tggtgactca gccacctacc tctgtgctgt gaggcccaca
1801 tcaggaggaa gctacatacc tacatttgga agaggaacca gccttattgt tcatccgtat
1861 atccagaacc ctgaccctgc agtgtatcaa ttgcgcgata gcaagtctag cgataaatcc
1921 gtgtgtctgt ttactgactt cgactcacag acgaacgtca gtcagagcaa agacagcgac
1981 gtttacatta ccgataagac agtccttgat atgagatcca tggattttaa aagtaattct
2041 gcggttgctt ggtcaaataa gtccgatttc gcctgcgcca atgcttttaa taattccatc
2101 atccctgagg atacgttctt tccgtctcct gaaagttcct gtgatgtcaa gctggtcgag
2161 aaaagctttg aaacagatac gaacctaaac tttcaaaacc tgtcagtgat tgggttccga
```

2221 atcctcctcc tgaaagtggc cgggtttaat ctgctcatga cgctgcggct gtggtccagc

2281 tgactagagc tcgctgatca gcctcgactg tgccttctag ttgccagcca tctgttgttt

2341 gcccctcccc cgtgccttcc ttgaccctgg aaggtgccac tcccactgtc ctttcctaat

2401 aaaatgagga aattgcatcg cattgtctga gtaggtgtca ttctattctg gggggtgggg

2461 tggggcagga cagcaagggg gaggattggg aagagaatag caggcatgct ggggatacca

2521 gctgagagac tctaaatcca gtgacaagtc tgtctgccta ttcaccgatt ttgattctca

2581 aacaaatgtg tcacaaagta aggattctga tgtgtatatc acagacaaaa ctgtgctaga

2641 catgaggtct atggacttca agagcaacag tgctgtggcc tggagcaaca aatctgactt

2701 tgcatgtgca aacgccttca acaacagcat tattccagaa gacaccttct tccccagccc

2761 aggtaagggc agctttggtg ccttcgcagg ctgtttcctt gcttcaggaa tggccaaggt

2821 tctgcccaga gctctggtca atgatg

Protein sequence

SP TCRβ T2A SP TCRα

1 11 21 31 41 51

MSIGLLCCAA LSLLWAGPVN AGVTQTPKFQ VLKTGQSMTL QCAQDMNHEY MSWYRQDPGM

61 71 81 91 101 111

GLRLIHYSVG AGITDQGEVP NGYNVSRSTT EDFPLRLLSA APSQTSVYFC ASSYVGNTGE

121 131 141 151 161 171

LFFGEGSRLT VLEDLKNVFP PEVAVFEPSE AEISHTQKAT LVCLATGFYP DHVELSWWVN

181 191 201 211 221 231

GKEVHSGVST DPQPLKEQPA LNDSRYCLSS RLRVSATFWQ NPRNHFRCQV QFYGLSENDE

241 251 261 271 281 291

WTQDRAKPVT QIVSAEAWGR ADCGFTSESY QQGVLSATIL YEILLGKATL YAVLVSALVL

301 311 321 331 341 351

MAMVKRKDSR GGSGEGRGSL LTCGDVEENP GPMETLLGLL ILWLQLQWVS SKQEVTQIPA

361 371 381 391 401 411

ALSVPEGENL VLNCSFTDSA IYNLQWFRQD PGKGLTSLLL IQSSQREQTS GRLNASLDKS

421 431 442 451 461 471

SGRSTLYIAA SQPGDSATYL CAVRPTSGGS YIPTFGRGTS LIVHPYIQNP DPAVYQLRDS

481 491 501 511 521 531

KSSDKSVCLF TDFDSQTNVS QSKDSDVYIT DKTVLDMRSM DFKSNSAVAW SNKSDFACAN

541 551 561 571 581 591

AFNNSIIPED TFFPSPESSC DVKLVEKSFE TDTNLNFQNL SVIGFRILLL KVAGFNLLMT

601 607

LRLWSS*

Data S7: c58c61 1G4hi TCR CRISPR construct (GenBank: PP746590)

Nucleotide sequence

```
       1 ctgcctttac tctgccagag ttatattgct ggggttttga agaagatcct attaaataaa
      61 agaataagca gtattattaa gtagccctgc atttcaggtt tccttgagtg gcaggccagg
     121 cctggccgtg aacgttcact gaaatcatgg cctcttggcc aagattgata gcttgtgcct
     181 gtccctgagt cccagtccat cacgagcagc tggtttctaa gatgctattt cccgtataaa
     241 gcatgagacc gtgacttgcc agccccacag agccccgccc ttgtccatca ctggcatctg
     301 gactccagcc tgggttgggg caaagaggga aatgagatca tgtcctaacc ctgatcctct
     361 tgtcccacag atatccagaa ccctgaccct gccgtgggca gcggcgccac caacttcagc
     421 ctgctgaagc aggccggcga cgtggaagag aaccccgggc ccatgtctat cggcctgctg
     481 tgttgtgccg ctctgtctct gctttgggcc ggacctgtta atgccggcgt gacccagaca
     541 cctaagttcc aggtgctgaa aaccggccag agcatgaccc tgcagtgcgc ccaggatatg
     601 aaccacgagt acatgagctg gtacagacag gaccctggca tgggcctgag actgatccac
     661 tactctgtgg ccatccagac caccgacaga ggcgaagtgc ccaacggcta caacgtgtcc
     721 agaagcacca tcgaggactt cccactgaga ctgctgtctg ccgctcctag ccagaccagc
     781 gtgtactttt gtgccagcag ctacctgggc aacaccggcg agctgttttt tggcgagggc
     841 agcagactga ccgtgctgga ggacctgaaa aacgtgttcc cacccgaggt cgctgtgttt
     901 gagccatcag aagcagagat ctcccacacc caaaaggcca cactggtgtg cctggccaca
     961 ggcttctacc ccgaccacgt ggagctgagc tggtgggtga atgggaagga ggtgcacagt
    1021 ggggtcagca cagacccgca gcccctcaag gagcagcccg ccctcaatga ctccagatac
    1081 tgcctgagca gccgcctgag ggtctcggcc accttctggc agaacccccg caaccacttc
    1141 cgctgtcaag tccagttcta cgggctctcg gagaatgacg agtggaccca agatagggcc
    1201 aaacctgtca cccagatcgt cagcgccgag gcctggggta gagcagactg tggcttcacc
    1261 tccgagtctt accagcaagg ggtcctgtct gccaccatcc tctatgagat cttgctaggg
    1321 aaggccacct tgtatgccgt gctggtcagt gccctcgtgc tgatggccat ggtcaagaga
    1381 aaggattcca gaggcggcag cggcgagggc agaggaagtc tgctaacatg cggtgacgtc
    1441 gaggagaatc ctggacctat ggaaacactg ctgggcctgc tgatcctgtg gctgcaactg
    1501 caatgggtgt cctccaagca agaagtgact cagatcccag ccgctctgag tgtgcctgag
    1561 ggcgaaaacc tggtcctgaa ctgcagcttc accgacagcg ccatctacaa cctgcagtgg
    1621 ttcagacaag atcccggcaa gggactgaca agcctgctgc tgattacccc ttggcagaga
    1681 gagcagacct ccggcagact gaatgccagc ctggataaga gcagcggccg gtctacactg
    1741 tatatcgccg cttctcagcc tggcgatagc gccacatatc tgtgtgccgt cagacccctg
    1801 ctggacggca catatatccc tacctttggc agaggcacca gcctgatcgt gcacccctac
    1861 atccagaacc ctgaccctgc agtgtatcaa ttgcgcgata gcaagtctag cgataaatcc
    1921 gtgtgtctgt ttactgactt cgactcacag acgaacgtca gtcagagcaa agacagcgac
    1981 gtttacatta ccgataagac agtccttgat atgagatcca tggattttaa aagtaattct
    2041 gcggttgctt ggtcaaataa gtccgatttc gcctgcgcca atgcttttaa taattccatc
    2101 atccctgagg atacgttctt tccgtctcct gaaagttcct gtgatgtcaa gctggtcgag
    2161 aaaagctttg aaacagatac gaacctaaac tttcaaaacc tgtcagtgat tgggttccga
```

2221 atcctcctcc tgaaagtggc cgggtttaat ctgctcatga cgctgcggct gtggtccagc
2281 tgactagagc tcgctgatca gcctcgactg tgccttctag ttgccagcca tctgttgttt
2341 gcccctcccc cgtgccttcc ttgaccctgg aaggtgccac tcccactgtc ctttcctaat
2401 aaaatgagga aattgcatcg cattgtctga gtaggtgtca ttctattctg gggggtgggg
2461 tggggcagga cagcaagggg gaggattggg aagagaatag caggcatgct ggggatacca
2521 gctgagagac tctaaatcca gtgacaagtc tgtctgccta ttcaccgatt ttgattctca
2581 aacaaatgtg tcacaaagta aggattctga tgtgtatatc acagacaaaa ctgtgctaga
2641 catgaggtct atggacttca agagcaacag tgctgtggcc tggagcaaca aatctgactt
2701 tgcatgtgca aacgccttca acaacagcat tattccagaa gacaccttct tccccagccc
2761 aggtaagggc agctttggtg ccttcgcagg ctgtttcctt gcttcaggaa tggccaaggt
2821 tctgcccaga gctctggtca atgatg

Protein sequence

SP TCRβ T2A SP TCRα

1 11 21 31 41 51
MSIGLLCCAA LSLLWAGPVN AGVTQTPKFQ VLKTGQSMTL QCAQDMNHEY MSWYRQDPGM
61 71 81 91 101 111
GLRLIHYSVA IQTTDRGEVP NGYNVSRSTI EDFPLRLLSA APSQTSVYFC ASSYLGNTGE
121 131 141 151 161 171
LFFGEGSRLT VLEDLKNVFP PEVAVFEPSE AEISHTQKAT LVCLATGFYP DHVELSWWVN
181 191 201 211 221 231
GKEVHSGVST DPQPLKEQPA LNDSRYCLSS RLRVSATFWQ NPRNHFRCQV QFYGLSENDE
241 251 261 271 281 291
WTQDRAKPVT QIVSAEAWGR ADCGFTSESY QQGVLSATIL YEILLGKATL YAVLVSALVL
301 311 321 331 341 351
MAMVKRKDSR GGSGEGRGSL LTCGDVEENP GPMETLLGLL ILWLQLQWVS SKQEVTQIPA
361 371 381 391 401 411
ALSVPEGENL VLNCSFTDSA IYNLQWFRQD PGKGLTSLLL ITPWQREQTS GRLNASLDKS
421 431 442 451 461 471
SGRSTLYIAA SQPGDSATYL CAVRPLLDGT YIPTFGRGTS LIVHPYIQNP DPAVYQLRDS
481 491 501 511 521 531
KSSDKSVCLF TDFDSQTNVS QSKDSDVYIT DKTVLDMRSM DFKSNSAVAW SNKSDFACAN
541 551 561 571 581 591
AFNNSIIPED TFFPSPESSC DVKLVEKSFE TDTNLNFQNL SVIGFRILLL KVAGFNLLMT
601 607
LRLWSS*

Data S8: RA14 TCR CRISPR construct (GenBank: PP746591)

Nucleotide sequence

```
        1 ctgcctttac tctgccagag ttatattgct ggggttttga agaagatcct attaaataaa
       61 agaataagca gtattattaa gtagccctgc atttcaggtt tccttgagtg gcaggccagg
      121 cctggccgtg aacgttcact gaaatcatgg cctcttggcc aagattgata gcttgtgcct
      181 gtccctgagt cccagtccat cacgagcagc tggtttctaa gatgctattt cccgtataaa
      241 gcatgagacc gtgacttgcc agccccacag agccccgccc ttgtccatca ctggcatctg
      301 gactccagcc tgggttgggg caaagaggga aatgagatca tgtcctaacc ctgatcctct
      361 tgtcccacag atatccagaa ccctgaccct gccgtgggca gcggcgccac caacttcagc
      421 ctgctgaagc aggccggcga cgtggaagag aaccccgggc ccatgagcat cggcctcctg
      481 tgctgtgcag ccttgtctct cctgtgggca ggtccagtga atgctggtgt cactcagacc
      541 ccaaaattcc aggtcctgaa gacaggacag agcatgacac tgcagtgtgc ccaggatatg
      601 aaccatgaat acatgtcctg gtatcgacaa gacccaggca tggggctgag gctgattcat
      661 tactcagttg gtgctggtat cactgaccaa ggagaagtcc ccaatggcta caatgtctcc
      721 agatcaacca cagaggattt cccgctcagg ctgctgtcgg ctgctccctc ccagacatct
      781 gtgtacttct gtgccagcag tcccgtgaca gggggcatct atggctacac cttcggttcg
      841 gggaccaggt taaccgttgt agaggacctg aaaaacgtgt tcccacccga ggtcgctgtg
      901 tttgagccat cagaagcaga gatctcccac acccaaaagg ccacactggt gtgcctggcc
      961 acaggcttct accccgacca cgtggagctg agctggtggg tgaatgggaa ggaggtgcac
     1021 agtggggtca gcacagaccc gcagcccctc aaggagcagc ccgccctcaa tgactccaga
     1081 tactgcctga gcagccgcct gagggtctcg gccaccttct ggcagaaccc ccgcaaccac
     1141 ttccgctgtc aagtccagtt ctacgggctc tcggagaatg acgagtggac ccaagatagg
     1201 gccaaacctg tcacccagat cgtcagcgcc gaggcctggg gtagagcaga ctgtggcttc
     1261 acctccgagt cttaccagca aggggtcctg tctgccacca tcctctatga gatcttgcta
     1321 gggaaggcca ccttgtatgc cgtgctggtc agtgccctcg tgctgatggc catggtcaag
     1381 agaaaggatt ccagaggcgg cagcggcgag ggcagaggaa gtctgctaac atgcggtgac
     1441 gtcgaggaga atcctggacc tatggagaag aatcctttgg cagccccatt actaatcctc
     1501 tggtttcatc ttgactgcgt gagcagcata ctgaacgtgg aacaaagtcc tcagtcactg
     1561 catgttcagg agggagacag caccaatttc acctgcagct tcccttccag caatttttat
     1621 gccttacact ggtacagatg ggaaactgca aaaagccccg aggccttgtt tgtaatgact
     1681 ttaaatgggg atgaaaagaa gaaaggacga ataagtgcca ctcttaatac caaggagggt
     1741 tacagctatt tgtacatcaa aggttctcag cctgaagact cagccacata cctctgtgcc
     1801 cgcaacaccg gtaaccagtt ctattttggg acagggacaa gtttgacggt cattccaaat
     1861 atccagaacc ctgaccctgc agtgtatcaa ttgcgcgata gcaagtctag cgataaatcc
     1921 gtgtgtctgt ttactgactt cgactcacag acgaacgtca gtcagagcaa agacagcgac
     1981 gtttacatta ccgataagac agtccttgat atgagatcca tggattttaa aagtaattct
     2041 gcggttgctt ggtcaaataa gtccgatttc gcctgcgcca atgcttttaa taattccatc
     2101 atccctgagg atacgttctt tccgtctcct gaaagttcct gtgatgtcaa gctggtcgag
     2161 aaaagctttg aaacagatac gaacctaaac tttcaaaacc tgtcagtgat tgggttccga
```

2221 atcctcctcc tgaaagtggc cgggtttaat ctgctcatga cgctgcggct gtggtccagc
2281 tgactagagc tcgctgatca gcctcgactg tgccttctag ttgccagcca tctgttgttt
2341 gcccctcccc cgtgccttcc ttgaccctgg aaggtgccac tcccactgtc ctttcctaat
2401 aaaatgagga aattgcatcg cattgtctga gtaggtgtca ttctattctg gggggtgggg
2461 tggggcagga cagcaagggg gaggattggg aagagaatag caggcatgct ggggatacca
2521 gctgagagac tctaaatcca gtgacaagtc tgtctgccta ttcaccgatt ttgattctca
2581 aacaaatgtg tcacaaagta aggattctga tgtgtatatc acagacaaaa ctgtgctaga
2641 catgaggtct atggacttca agagcaacag tgctgtggcc tggagcaaca aatctgactt
2701 tgcatgtgca aacgccttca acaacagcat tattccagaa gacaccttct tccccagccc
2761 aggtaagggc agctttggtg ccttcgcagg ctgtttcctt gcttcaggaa tggccaaggt
2821 tctgcccaga gctctggtca atgatg

Protein sequence

SP TCRβ T2A SP TCRα

1 11 21 31 41 51
MSIGLLCCAA LSLLWAGPVN AGVTQTPKFQ VLKTGQSMTL QCAQDMNHEY MSWYRQDPGM
61 71 81 91 101 111
GLRLIHYSVG AGITDQGEVP NGYNVSRSTT EDFPLRLLSA APSQTSVYFC ASSPVTGGIY
121 131 141 151 161 171
GYTFGSGTRL TVVEDLKNVF PPEVAVFEPS EAEISHTQKA TLVCLATGFY PDHVELSWWV
181 191 201 211 221 231
NGKEVHSGVS TDPQPLKEQP ALNDSRYCLS SRLRVSATFW QNPRNHFRCQ VQFYGLSEND
241 251 261 271 281 291
EWTQDRAKPV TQIVSAEAWG RADCGFTSES YQQGVLSATI LYEILLGKAT LYAVLVSALV
301 311 321 331 341 351
LMAMVKRKDS RGGSGEGRGS LLTCGDVEEN PGPMEKNPLA APLLILWFHL DCVSSILNVE
361 371 381 391 401 411
QSPQSLHVQE GDSTNFTCSF PSSNFYALHW YRWETAKSPE ALFVMTLNGD EKKKGRISAT
421 431 442 451 461 471
LNTKEGYSYL YIKGSQPEDS ATYLCARNTG NQFYFGTGTS LTVIPNIQNP DPAVYQLRDS
481 491 501 511 521 531
KSSDKSVCLF TDFDSQTNVS QSKDSDVYIT DKTVLDMRSM DFKSNSAVAW SNKSDFACAN
541 551 561 571 581 591
AFNNSIIPED TFFPSPESSC DVKLVEKSFE TDTNLNFQNL SVIGFRILLL KVAGFNLLMT
601 607
LRLWSS*

Data S9: T1 STAR$_{nat}$ T2A copGFP CRISPR construct (GenBank: PP746592)

Nucleotide sequence

```
        1 ctgcctttac tctgccagag ttatattgct ggggttttga agaagatcct attaaataaa
       61 agaataagca gtattattaa gtagccctgc atttcaggtt tccttgagtg gcaggccagg
      121 cctggccgtg aacgttcact gaaatcatgg cctcttggcc aagattgata gcttgtgcct
      181 gtccctgagt cccagtccat cacgagcagc tggtttctaa gatgctattt cccgtataaa
      241 gcatgagacc gtgacttgcc agccccacag agccccgccc ttgtccatca ctggcatctg
      301 gactccagcc tgggttgggg caaagaggga aatgagatca tgtcctaacc ctgatcctct
      361 tgtcccacag atatccagaa ccctgaccct gccgtgggca gcggcgccac caacttcagc
      421 ctgctgaagc aggccggcga cgtggaagag aaccccgggc ccatgggcag ctggaccctg
      481 tgttgcgtgt ccctgtgcat cctggtggct aagcacaccc agagcgagct gacccagccc
      541 agaagcgtgt ccggcagccc tggccagagc gtgaccatca gctgtaccgg caccgaaaga
      601 gatgtgggcg gctacaacta cgtgtcctgg tatcagcagc accccggcaa ggcccctaag
      661 ctgatcatcc acgacgtgat cgagagaagc agcggcgtgc ccgacagatt cagcggcagc
      721 aagagcggca acaccgccag cctgaccatc tctggcctcc aggccgagga cgaggccgac
      781 tactactgct ggagcttcgc cggcggatac tacgtgttcg gcaccggcac cgacgtgacc
      841 gtggaggatc tgagaaatgt gactccaccc aaggtctcct tgtttgagcc atcaaaagca
      901 gagattgcaa acaaacaaaa ggctaccctc gtgtgcttgg ccaggggctt cttccctgac
      961 cacgtggagc tgagctggtg ggtgaatggc aaggaggtcc acagtggggt ctgcacggac
     1021 cctcaggcct acaaggagag caattatagc tactgcctga gcagccgcct gagggtctct
     1081 gctaccttct ggcacaatcc tcgaaaccac ttccgctgcc aagtgcagtt ccatgggctt
     1141 tcagaggagg acaagtggcc agagggctca cccaaacctg tcacacagaa catcagtgca
     1201 gaggcctggg gccgagcaga ctgtggaatc acttcagcat cctatcatca gggggttctg
     1261 tctgcaacca tcctctatga gatcctactg gggaaggcca ccctatatgc tgtgctggtc
     1321 agtggcctgg tgctgatggc catggtcaag aaaaaaaatt ccggcagcgg cgagggcaga
     1381 ggaagtctgc taacatgcgg tgacgtcgag gagaatcctg gacctatgat gaagtccctg
     1441 agggtgctgc tggtgatcct ctggctgcag ctgagctggg tgtggtccca ggaggtgcag
     1501 ctgctggagt ctggcggcgg actggtgcag cctggcggca gcctgagact gagctgtgcc
     1561 gccagcggct tcaccttcag cacctaccag atgagctggg tgcggcaggc ccctggcaag
     1621 ggcctggagt gggtgtccgg catcgtgtcc agcggcggca gcaccgccta cgccgacagc
     1681 gtgaagggca ggttcaccat cagccgggac aacagcaaga acaccctgta cctgcagatg
     1741 aacagcctga gagccgagga caccgccgtg tactactgtg ccggcgagct gctgccctac
     1801 tacggcatgg atgtgtgggg ccagggcacc accgtgacag tgagcatcca gaacccagaa
     1861 cctgctgtgt accagttaaa agatcctcgg tctcaggaca gcaccctctg cctgttcacc
     1921 gactttgact cccaaatcaa tgtgccgaaa accatggaat ctggaacgtt catcactgac
     1981 aaatgcgtgc tggacatgaa agctatggat tccaagagca atggggccat tgcctggagc
     2041 aaccagacaa gcttcacctg ccaagatatc ttcaaagaga ccaacgccac ctaccccagt
     2101 tcagacgttc cctgtgatgc cacgttgact gagaaaagct ttgaaacaga tatgaaccta
     2161 aactttcaaa acctgtcagt tatgggactc cgaatcctcc tgctgaaagt agccggattt
```

2221 aacctgctca tgacgctgag gctgtggtcc agtggcagcg gcgagggcag aggaagtctt
2281 ctaacatgcg gtgacgtgga ggagaatccc ggcccttccg gaatggagag cgacgagagc
2341 ggcctgcccg ccatggagat cgagtgccgc atcaccggca ccctgaacgg cgtggagttc
2401 gagctggtgg gcggcggaga gggcaccccc aagcagggcc gcatgaccaa caagatgaag
2461 agcaccaaag gcgccctgac cttcagcccc tacctgctga gccacgtgat gggctacggc
2521 ttctaccact tcggcaccta ccccagcggc tacgagaacc ccttcctgca cgccatcaac
2581 aacggcggct acaccaacac ccgcatcgag aagtacgagg acggcggcgt gctgcacgtg
2641 agcttcagct accgctacga ggccggccgc gtgatcggcg acttcaaggt ggtgggcacc
2701 ggcttccccg aggacagcgt gatcttcacc gacaagatca tccgcagcaa cgccaccgtg
2761 gagcacctgc accccatggg cgataacgtg ctggtgggca gcttcgcccg caccttcagc
2821 ctgcgcgacg gcggctacta cagcttcgtg gtggacagcc acatgcactt caagagcgcc
2881 atccacccca gcatcctgca gaacgggggc cccatgttcg ccttccgccg cgtggaggag
2941 ctgcacagca acaccgagct gggcatcgtg gagtaccagc acgccttcaa gacccccatc
3001 gccttcgcca gatcccgcgc tcagtcgtcc aattctgccg tggacggcac cgccggaccc
3061 ggctccaccg gatctcgcta gctagagctc gctgatcagc ctcgactgtg ccttctagtt
3121 gccagccatc tgttgtttgc ccctcccccg tgccttcctt gaccctggaa ggtgccactc
3181 ccactgtcct ttcctaataa aatgaggaaa ttgcatcgca ttgtctgagt aggtgtcatt
3241 ctattctggg gggtggggtg gggcaggaca gcaaggggga ggattgggaa gagaatagca
3301 ggcatgctgg ggataccagc tgagagactc taaatccagt gacaagtctg tctgcctatt
3361 caccgatttt gattctcaaa caaatgtgtc acaaagtaag gattctgatg tgtatatcac
3421 agacaaaact gtgctagaca tgaggtctat ggacttcaag agcaacagtg ctgtggcctg
3481 gagcaacaaa tctgactttg catgtgcaaa cgccttcaac aacagcatta ttccagaaga
3541 caccttcttc cccagcccag gtaagggcag ctttggtgcc ttcgcaggct gtttccttgc
3601 ttcaggaatg gccaaggttc tgcccagagc tctggtcaat gatg

Data S10: T1 STAR$_{mir}$ T2A copGFP CRISPR construct (GenBank: PP746593)

Nucleotide sequence

```
        1 ctgcctttac tctgccagag ttatattgct ggggttttga agaagatcct attaaataaa
       61 agaataagca gtattattaa gtagccctgc atttcaggtt tccttgagtg gcaggccagg
      121 cctggccgtg aacgttcact gaaatcatgg cctcttggcc aagattgata gcttgtgcct
      181 gtccctgagt cccagtccat cacgagcagc tggtttctaa gatgctattt cccgtataaa
      241 gcatgagacc gtgacttgcc agccccacag agccccgccc ttgtccatca ctggcatctg
      301 gactccagcc tgggttgggg caaagaggga aatgagatca tgtcctaacc ctgatcctct
      361 tgtcccacag atatccagaa ccctgaccct gccgtgggca gcggcgccac caacttcagc
      421 ctgctgaagc aggccggcga cgtggaagag aaccccgggc ccatgggcag ctggaccctg
      481 tgttgcgtgt ccctgtgcat cctggtggct aagcacaccg aggtgcagct gctggagtct
      541 ggcggcggac tggtgcagcc tggcggcagc ctgagactga gctgtgccgc cagcggcttc
      601 accttcagca cctaccagat gagctgggtg cggcaggccc ctggcaaggg cctggagtgg
      661 gtgtccggca tcgtgtccag cggcggcagc accgcctacg ccgacagcgt gaagggcagg
      721 ttcaccatca gccgggacaa cagcaagaac accctgtacc tgcagatgaa cagcctgaga
      781 gccgaggaca ccgccgtgta ctactgtgcc ggcgagctgc tgccctacta cggcatggat
      841 gtgtggggcc agggcaccac cgtgacagtg agcgaggatc tgagaaatgt gactccaccc
      901 aaggtctcct tgtttgagcc atcaaaagca gagattgcaa acaaacaaaa ggctaccctc
      961 gtgtgcttgg ccaggggctt cttccctgac cacgtggagc tgagctggtg ggtgaatggc
     1021 aaggaggtcc acagtggggt ctgcacggac cctcaggcct acaaggagag caattatagc
     1081 tactgcctga gcagccgcct gagggtctct gctaccttct ggcacaatcc tcgaaaccac
     1141 ttccgctgcc aagtgcagtt ccatgggctt tcagaggagg acaagtggcc agagggctca
     1201 cccaaacctg tcacacagaa catcagtgca gaggcctggg gccgagcaga ctgtggaatc
     1261 acttcagcat cctatcatca gggggttctg tctgcaacca tcctctatga gatcctactg
     1321 gggaaggcca ccctatatgc tgtgctggtc agtggcctgg tgctgatggc catggtcaag
     1381 aaaaaaaatt ccggcagcgg cgagggcaga ggaagtctgc taacatgcgg tgacgtcgag
     1441 gagaatcctg gacctatgat gaagtccctg agggtgctgc tggtgatcct ctggctgcag
     1501 ctgagctggg tgtggtccca gcagagcgag ctgacccagc ccagaagcgt gtccggcagc
     1561 cctggccaga gcgtgaccat cagctgtacc ggcaccgaaa gagatgtggg cggctacaac
     1621 tacgtgtcct ggtatcagca gcaccccggc aaggccccta agctgatcat ccacgacgtg
     1681 atcgagagaa gcagcggcgt gcccgacaga ttcagcggca gcaagagcgg caacaccgcc
     1741 agcctgacca tctctggcct ccaggccgag gacgaggccg actactactg ctggagcttc
     1801 gccggcggat actacgtgtt cggcaccggc accgacgtga ccgtgatcca gaacccagaa
     1861 cctgctgtgt accagttaaa agatcctcgg tctcaggaca gcaccctctg cctgttcacc
     1921 gactttgact cccaaatcaa tgtgccgaaa accatggaat ctggaacgtt catcactgac
     1981 aaatgcgtgc tggacatgaa agctatggat tccaagagca atggggccat tgcctggagc
     2041 aaccagacaa gcttcacctg ccaagatatc ttcaaagaga ccaacgccac ctaccccagt
     2101 tcagacgttc cctgtgatgc cacgttgact gagaaaagct ttgaaacaga tatgaaccta
```

2161 aactttcaaa acctgtcagt tatgggactc cgaatcctcc tgctgaaagt agccggattt
2221 aacctgctca tgacgctgag gctgtggtcc agtggcagcg gcgagggcag aggaagtctt
2281 ctaacatgcg gtgacgtgga ggagaatccc ggcccttccg gaatggagag cgacgagagc
2341 ggcctgcccg ccatggagat cgagtgccgc atcaccggca ccctgaacgg cgtggagttc
2401 gagctggtgg gcggcggaga gggcaccccc aagcagggcc gcatgaccaa caagatgaag
2461 agcaccaaag gcgccctgac cttcagcccc tacctgctga gccacgtgat gggctacggc
2521 ttctaccact tcggcaccta ccccagcggc tacgagaacc ccttcctgca cgccatcaac
2581 aacggcggct acaccaacac ccgcatcgag aagtacgagg acggcggcgt gctgcacgtg
2641 agcttcagct accgctacga ggccggccgc gtgatcggcg acttcaaggt ggtgggcacc
2701 ggcttccccg aggacagcgt gatcttcacc gacaagatca tccgcagcaa cgccaccgtg
2761 gagcacctgc accccatggg cgataacgtg ctggtgggca gcttcgcccg caccttcagc
2821 ctgcgcgacg gcggctacta cagcttcgtg gtggacagcc acatgcactt caagagcgcc
2881 atccacccca gcatcctgca gaacgggggc cccatgttcg ccttccgccg cgtggaggag
2941 ctgcacagca acaccgagct gggcatcgtg gagtaccagc acgccttcaa gaccccccatc
3001 gccttcgcca gatcccgcgc tcagtcgtcc aattctgccg tggacggcac cgccggaccc
3061 ggctccaccg gatctcgcta gctagagctc gctgatcagc ctcgactgtg ccttctagtt
3121 gccagccatc tgttgtttgc ccctcccccg tgccttcctt gaccctggaa ggtgccactc
3181 ccactgtcct ttcctaataa aatgaggaaa ttgcatcgca ttgtctgagt aggtgtcatt
3241 ctattctggg gggtggggtg gggcaggaca gcaaggggga ggattgggaa gagaatagca
3301 ggcatgctgg ggataccagc tgagagactc taaatccagt gacaagtctg tctgcctatt
3361 caccgatttt gattctcaaa caaatgtgtc acaaagtaag gattctgatg tgtatatcac
3421 agacaaaact gtgctagaca tgaggtctat ggacttcaag agcaacagtg ctgtggcctg
3481 gagcaacaaa tctgactttg catgtgcaaa cgccttcaac aacagcatta ttccagaaga
3541 caccttcttc cccagcccag gtaagggcag ctttggtgcc ttcgcaggct gtttccttgc
3601 ttcaggaatg gccaaggttc tgcccagagc tctggtcaat gatg

Data S11: T1 BBz CAR T2A copGFP CRISPR construct (GenBank: PP746594)

Nucleotide sequence

```
   1 ctgcctttac tctgccagag ttatattgct ggggttttga agaagatcct attaaataaa
  61 agaataagca gtattattaa gtagccctgc atttcaggtt tccttgagtg gcaggccagg
 121 cctggccgtg aacgttcact gaaatcatgg cctcttggcc aagattgata gcttgtgcct
 181 gtccctgagt cccagtccat cacgagcagc tggtttctaa gatgctattt cccgtataaa
 241 gcatgagacc gtgacttgcc agccccacag agccccgccc ttgtccatca ctggcatctg
 301 gactccagcc tgggttgggg caaagaggga aatgagatca tgtcctaacc ctgatcctct
 361 tgtcccacag atatccagaa ccctgaccct gccgtgggca gcggcgccac caacttcagc
 421 ctgctgaagc aggccggcga cgtggaagag aaccccgggc ccatgcttct cctggtgaca
 481 agccttctgc tctgtgagtt accacaccca gcattcctcc tgatcccaat ggcggaggtg
 541 cagctgctgg agtctggcgg cggactggtg cagcctggcg gcagcctgag actgagctgt
 601 gccgccagcg gcttcacctt cagcacctac cagatgagct gggtgcggca ggcccctggc
 661 aagggcctgg agtgggtgtc cggcatcgtg tccagcggcg gcagcaccgc ctacgccgac
 721 agcgtgaagg gcaggttcac catcagccgg gacaacagca agaacaccct gtacctgcag
 781 atgaacagcc tgagagccga ggacaccgcc gtgtactact gtgccggcga gctgctgccc
 841 tactacggca tggatgtgtg gggccagggc accaccgtga cagtgagcag cgccaagacc
 901 acccccaagc tggaggaggg cgagttcagc gaggccagag tgcagagcga gctgacccag
 961 cccagaagcg tgtccggcag ccctggccag agcgtgacca tcagctgtac cggcaccgaa
1021 agagatgtgg gcggctacaa ctacgtgtcc tggtatcagc agcaccccgg caaggcccct
1081 aagctgatca tccacgacgt gatcgagaga agcagcggcg tgcccgacag attcagcggc
1141 agcaagagcg gcaacaccgc cagcctgacc atctctggcc tccaggccga ggacgaggcc
1201 gactactact gctggagctt cgccggcgga tactacgtgt tcggcaccgg caccgacgtg
1261 accgtgctgg gccagcccaa ggccaacccc acactcgaga attggtcaca tcctcaattt
1321 gaaaaaggtg gaggcggttc acccgggaca accacccctg cccctagacc tcccacccca
1381 gccccaacaa ttgccagcca gcctctgtct ctgcggcccg aagcttgtag acctgctgcc
1441 ggcggagccg tgcacaccag aggactggat ttcgcctgcg acatctacat ctgggcccct
1501 ctggccggca catgtggcgt gctgctcctc agcctggtca tcaccctgta ctgcaagcgg
1561 ggcagaaaga aactgctcta catcttcaag cagcccttca tgcggcccgt gcagaccaca
1621 caggaagagg acggctgctc ctgcagattc cccgaggaag aagaaggcgg ctgcgagctg
1681 agagtgaagt tcagcagatc cgccgacgcc cctgcctacc agcagggaca gaaccagctg
1741 tacaacgagc tgaacctggg cagacgggaa gagtacgacg tgctggacaa gcggagaggc
1801 agagatcccg agatgggcgg caagcccaga cggaagaatc cccaggaagg cctgtataac
1861 gaactgcaga aagacaagat ggccgaggcc tacagcgaga tcggaatgaa gggcgagcgg
1921 agaagaggca agggccacga tggcctgtac cagggcctga gcaccgccac caaggacacc
1981 tacgatgccc tgcacatgca ggccctgcca cccagaggct cgggcgaggg cagaggaagt
2041 cttctaacat gcggtgacgt ggaggagaat cccggccctt ccggaatgga gagcgacgag
2101 agcggcctgc ccgccatgga gatcgagtgc cgcatcaccg gcaccctgaa cggcgtggag
```

2161 ttcgagctgg tgggcggcgg agagggcacc cccaagcagg gccgcatgac caacaagatg
2221 aagagcacca aaggcgccct gaccttcagc ccctacctgc tgagccacgt gatgggctac
2281 ggcttctacc acttcggcac ctaccccagc ggctacgaga accccttcct gcacgccatc
2341 aacaacggcg gctacaccaa cacccgcatc gagaagtacg aggacggcgg cgtgctgcac
2401 gtgagcttca gctaccgcta cgaggccggc cgcgtgatcg gcgacttcaa ggtggtgggc
2461 accggcttcc ccgaggacag cgtgatcttc accgacaaga tcatccgcag caacgccacc
2521 gtggagcacc tgcaccccat gggcgataac gtgctggtgg gcagcttcgc ccgcaccttc
2581 agcctgcgcg acggcggcta ctacagcttc gtggtggaca gccacatgca cttcaagagc
2641 gccatccacc ccagcatcct gcagaacggg ggccccatgt tcgccttccg ccgcgtggag
2701 gagctgcaca gcaacaccga gctgggcatc gtggagtacc agcacgcctt caagaccccc
2761 atcgccttcg ccagatcccg cgctcagtcg tccaattctg ccgtggacgg caccgccgga
2821 cccggctcca ccggatctcg ctagctagag ctcgctgatc agcctcgact gtgccttcta
2881 gttgccagcc atctgttgtt tgcccctccc ccgtgccttc cttgaccctg gaaggtgcca
2941 ctcccactgt cctttcctaa taaaatgagg aaattgcatc gcattgtctg agtaggtgtc
3001 attctattct ggggggtggg gtggggcagg acagcaaggg ggaggattgg gaagagaata
3061 gcaggcatgc tggggatacc agctgagaga ctctaaatcc agtgacaagt ctgtctgcct
3121 attcaccgat tttgattctc aaacaaatgt gtcacaaagt aaggattctg atgtgtatat
3181 cacagacaaa actgtgctag acatgaggtc tatggacttc aagagcaaca gtgctgtggc
3241 ctggagcaac aaatctgact ttgcatgtgc aaacgccttc aacaacagca ttattccaga
3301 agacaccttc ttccccagcc caggtaaggg cagctttggt gccttcgcag gctgtttcct
3361 tgcttcagga atggccaagg ttctgcccag agctctggtc aatgatg
